# Antifungal effects of a 1,3,4-thiadiazole derivative determined by cytochemical and vibrational spectroscopic studies

**DOI:** 10.1371/journal.pone.0222775

**Published:** 2019-09-30

**Authors:** Barbara Chudzik, Katarzyna Bonio, Wojciech Dabrowski, Daniel Pietrzak, Andrzej Niewiadomy, Alina Olender, Bożena Pawlikowska-Pawlęga, Mariusz Gagoś

**Affiliations:** 1 Department of Cell Biology, Institute of Biology and Biochemistry, Faculty of Biology and Biotechnology, Maria Curie-Skłodowska University, Lublin, Poland; 2 Department of Anaesthesiology and Intensive Therapy, Medical University of Lublin, Lublin, Poland; 3 Institute of Industrial Organic Chemistry, Warsaw, Poland; 4 Department of Chemistry, University of Life Sciences in Lublin, Lublin, Poland; 5 Chair and Department of Medical Microbiology, Medical University of Lublin, Lublin, Poland; 6 Department of Comparative Anatomy and Anthropology, Institute of Biology and Biochemistry, Faculty of Biology and Biotechnology, Maria Curie-Skłodowska University, Lublin, Poland; University of New South Wales, AUSTRALIA

## Abstract

Compounds belonging to the group of 5-substituted 4-(1,3,4-thiadiazol-2-yl) benzene-1,3-diols exhibit a broad spectrum of biological activity, including antibacterial, antifungal, and anticancer properties. The mechanism of the antifungal activity of compounds from this group has not been described to date. Among the large group of 5-substituted 4-(1,3,4-thiadiazol-2-yl) benzene-1,3-diol derivatives, the compound 4-(5-methyl-1,3,4-thiadiazole-2-yl) benzene-1,3-diol, abbreviated as C1, was revealed to be one of the most active agents against pathogenic fungi, simultaneously with the lowest toxicity to human cells. The C1 compound is a potent antifungal agent against different *Candida* species, including isolates resistant to azoles, and molds, with MIC_100_ values ranging from 8 to 96 μg/ml. The antifungal activity of the C1 compound involves disruption of the cell wall biogenesis, as evidenced by the inability of cells treated with C1 to maintain their characteristic cell shape, increase in size, form giant cells and flocculate. C1-treated cells were also unable to withstand internal turgor pressure causing protoplast material to leak out, exhibited reduced osmotic resistance and formed buds that were not covered with chitin. Disturbances in the chitin septum in the neck region of budding cells was observed, as well as an uneven distribution of chitin and β(1→3) glucan, and increased sensitivity to substances interacting with wall polymerization. The ATR-FTIR spectral shifts in cell walls extracted from *C*. *albicans* cells treated with the C1 compound suggested weakened interactions between the molecules of β(1→3) glucans and β(1→6) glucans, which may be the cause of impaired cell wall integrity. Significant spectral changes in the C1-treated cells were also observed in bands characteristic for chitin. The C1 compound did not affect the ergosterol content in *Candida* cells. Given the low cytotoxicity of the C1 compound to normal human dermal fibroblasts (NHDF), it is possible to use this compound as a therapeutic agent in the treatment of surface and gastrointestinal tract mycoses.

## Introduction

The incidence and lethality rates of fungal infections have increased dramatically over the recent years. In the United States, the number of deaths caused by invasive fungal infections rose by 320% within the last 17 years [1]. This phenomenon has been primarily associated with the constantly increasing number of transplant recipients receiving immunosuppression or patients treated for autoimmune diseases [2, 3]. Additionally, the development of invasive systemic mycoses is promoted by debilitation of the immune system by tumour chemotherapy, intensive antibiotic treatment, and primary or secondary immunodeficiency [4]. Superficial mycoses of the skin, nails, and mucous membranes are a common problem, which is especially frequent in children and asthma patients treated with steroid inhalations. In recent years, multi-drug resistant strains are increasingly being isolated in biological samples acquired from patients, and therefore a fungal infection can be associated with high mortality. A disturbing trend is the increasing incidence of infections with *Candida* species other than *albicans*, which often exhibit reduced sensitivity to polyenes and resistance to azoles [5–7]. These strains are also often resistant to echinocandins, which are the last-line of drugs in the treatment of invasive candidiasis [8]. According to data from the European Center for Disease Prevention and Control (ECDC), the resistance of *Candida* species, especially *C*. *glabrata* and *C*. *parapsilosis*, to echinocandins consequently increased from 4% in 2004 to 8% in 2014. This phenomenon is particularly worrying due to the high natural level of resistance of *Candida* species to azoles [5, 7, 9, 10]. The arsenal of potential antifungal drugs is very limited in comparison with antibacterial agents, because fungi are eukaryotic organisms and have few metabolic pathways differing from those in animal cells. The primary limitation of antifungal drugs that are currently used in clinical practice is their low selectivity and high toxicity as well as the constantly increasing number of resistant pathogens. Many authors note that the development of new antifungals is a key and indispensable task [4, 7, 10]. To address these needs, investigations of the antifungal activity of a new and poorly explored group of 5-substituted 4-(1,3,4-thiadiazol-2-yl) benzene-1,3-diols have been undertaken in our research team. Synthetic compounds from the group of 1,3,4-thiadiazole derivatives exhibit a broad spectrum of biological activity such as antifungal, antibacterial, antiviral, anticancer, anti-inflammatory, neuroprotective, or antihypertensive properties, which have been described in some review articles [11, 12]. The antifungal activity of 1,3,4-thiadiazole derivatives is poorly understood. Many authors have determined the values of minimal inhibitory concentrations (MICs) for newly synthesized derivatives; however, the cytotoxicity of these new synthetic compounds to human cells is rarely investigated simultaneously. To the best of our knowledge, there are no literature reports on the mechanism of the antifungal activity of compounds from the group of 1,3,4-thiadiazoles and their interactions with other antifungal agents.

**Fig 1:**
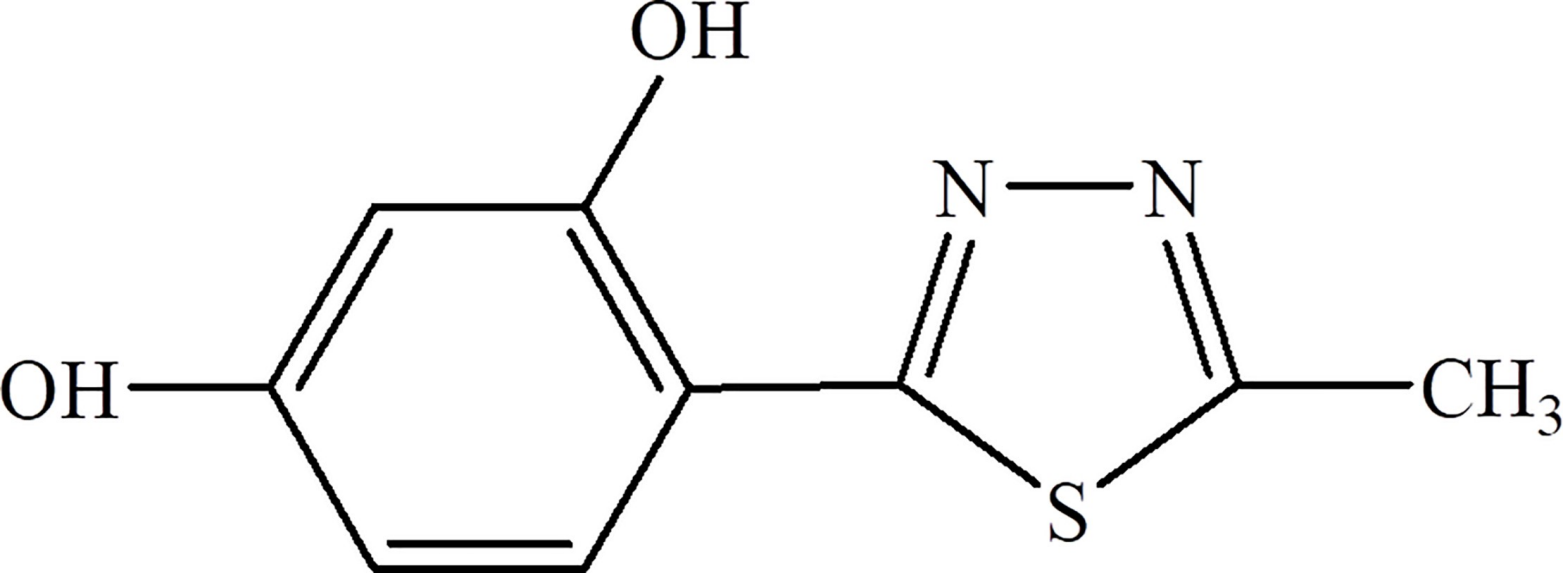
Formula of 4-(5-methyl-1,3,4-thiadiazole-2-yl) benzene-1,3-diol (the C1 compound).

The synthesis of compounds belonging to the group of 5-substituted 4-(1,3,4-thiadiazol-2-yl) benzene-1,3-diols has already been described in different publications [13–16]. The antifungal activity of these compounds was previously referred to as the average values of MICs against several species of *Candida* only, for which they range from a few dozen to several hundred μg/ml [14], and in relation to phytopathogenic fungi [16]. In our research team, a large group of 5-substituted 4-(1,3,4-thiadiazol-2-yl) benzene-1,3-diol derivatives was tested for high antifungal efficacy and low cytotoxicity to human cells. The compound 4-(5-methyl-1,3,4-thiadiazole-2-yl) benzene-1,3-diol, abbreviated as C1, was revealed to be one of the most active agents against fungal cells with the lowest toxicity to human cells. This compound has a relatively simple structure, in which the heterocyclic ring of 1,3,4-thiadiazole in the 2-position is substituted by benzene-1,3-diol and in the 5-position by a methyl group (Fig 1). In the present work, the antifungal activity of the C1 compound against different fungal species, including clinical isolates that are resistant to antifungals used in standard treatment, was studied in detail. In addition, the most likely antifungal activity mechanism of this compound against the *Candida* species is reported for the first time.

## Materials and methods

This study was approved by the Institutional Review Board and the Bioethics Committee of Medical University at Lublin, Poland. The synthesis of 4-(5-methyl-1,3,4-thiadiazole-2-yl) benzene-1,3-diol (C1) was previously described by Matysiak et al. [15].

### Isolation, identification, and susceptibility testing of pathogenic fungi

The isolates of pathogenic fungi were obtained from patients treated in the First Clinic of Intensive Care at the Medical University of Lublin, Poland. The isolation was carried out during routine microbiological screening and surveillance of hospitalized patients. Samples were transported in accordance with the intensive care unit (ICU) protocol (ZSZ procedure no: PM SE/010, 3rd edition, "Transport and collection of diagnostic materials for clinical trials"). Solid medium Sabouraud 2 Chloramphenicol agar (BioMerieux) was used for selective culturing of fungi from samples taken from the patients. Species identification of recovered colonies was performed using commercial chromogenic media Candida Chromogenic LAB-AGAR TM (BioMaxima, BioCORP) and ID 32 C (BioMerieux) tests, using the metabolic profile of individual species for identification. The antifungal susceptibility of the recovered isolates was studied with the microdilution method using the commercial ATB FUNGUS 3 kit (BioMerieux) with automatic reading of the results. For the reference strains of *Candida albicans* NCPF 3153 and *Candida parapsilosis* ATCC 22019 determination of the minimal inhibitory concentration (MIC) of amphotericin B (AmB) and fluconazole (FLC) was additionally performed, according to the procedure described in the section “Determination of the antifungal activity of the C1 compound”. The culture and identification of fungal isolates and testing their susceptibility to the standard antifungals were performed in the Laboratory of the Chair and Department of Medical Microbiology, at the Medical University in Lublin, Poland. This laboratory is included in the list of laboratories maintained by the National Chamber of Laboratory Diagnosticians under number 2683 on the list for the Lubelskie Voivodeship.

### Determination of the antifungal activity of the C1 compound

The identified isolates were stored at -70°C in cryovials VIABANK (BioMaxima) and used for further studies. Before each experiment, in order to obtain logarithmically growing fungal cells, the strains were precultured in a liquid YPD medium (1% yeast extract, 2% bactopeptone, 2% glucose) in 0.01 mol/l phosphate buffer pH 7.0, with shaking, at 35°C, for 24 h. The antifungal activity of the C1 compound was determined against the reference strains of *Candida albicans* NCPF 3153, *Candida parapsilosis* ATCC 22019, *Aspergillus niger* ATCC 16888, *Rhodotorula mucilaginosa* ATTC 22273, and *Trichophyton rubrum* 28188, as well as clinical isolates of *Candida* species resistant to selected antifungals, including *C*. *krusei*, *C*. *albicans*, *C*. *glabrata*, *C*. *tropicalis*, and *C*. *dubliniensis*b (the resistance profiles of the reference and clinical isolates are indicated in Table A in supplementary file). The broth microdilution method with identification of the minimal inhibitory concentration (MIC) was employed according to the standard methodological guidelines recommended by the Clinical and Laboratory Standards Institute (CLSI): document M27-A3 and M38-A2 for testing the susceptibility of yeasts and moulds, respectively [17, 18]. The method was modified by addition of 2% glucose to RPMI 1640 medium in order to optimize the fungal growth conditions. To determine the MIC values, an inoculum was prepared with the final density of 0.5 x 10^3^–2.5 x 10^3^ cells/ml for yeast and 0.4 x 10^4^–5 x 10^4^ for mold spores, diluted in RPMI 1640 medium, without phenol red and sodium bicarbonate (Sigma-Aldrich, catalog number: R 8755), buffered to pH 7.0 with 0.165 mol/l 3-(N-morpholino) propanesulfonic acid (MOPS) and supplemented with 2% of glucose. To obtain a stock solution, 1 mg of the C1 compound was dissolved in 100 μl of DMSO and further diluted in the culture medium to the concentration of 1 mg/ml. The stock solution was used to obtain twice-concentrated aliquots of the C1 compound in the culture medium on 96-well plates. The fungal inoculum (100 μl) was added to the wells containing 100 μl of two-fold serial the dilutions of the C1 compound, obtaining the final concentrations of the C1 compound ranging from 128 to 0.5 μg/ml. An additional final concentration of 96 μg/ml of C1 was also included in the assays. Some rows of the wells were left for the control fungal cells (no C1) and the medium-only control. The final concentration of DMSO did not exceed 0.5% and was added to the control samples as well. The plates were incubated for 48 h at 35°C or 27°C for the yeasts (*Candida* spp. and *Rhodotorula* spp.) and molds (*Aspergillus* spp. and *Trichophyton* spp.), respectively. Results were inspected visually and read spectrophotometrically using an E-max Microplate Reader at a wavelength of 600 nm (OD_600_). The concentrations of the compound inhibiting the microbial growth completely after 48 h of culture were assumed as the MIC_100_ values. and those inhibiting the growth by 70% were taken as the MIC_70_ values (see below). The MIC assays were repeated three times for each strain/species. In one repetition, each concentration of the compound was tested in 4 replicates (wells). The results presented are therefore the average of 12 individual measurements. The MIC_70_ value was determined by calculating the percentage of the control growth in each replication according to the following equation: (OD_600_ of the treated sample) / (OD_600_ of the untreated sample) x 100. Next, the average value of the percentage of the control growth was calculated. When the average percentage growth of the treated sample relative to the control sample was 30% or lower, the concentration was taken as MIC_70_ (70% growth inhibition).

The percentage inhibition of fungal growth by the different concentrations of the C1 compound after 24 and 48 h of incubation, in relation to the control, was measured for the reference strains of *C*. *albicans* NCPF 3153 and *C*. *parapsilosis* ATCC 22019. Cultures were prepared on 96-well plates according to the procedure described for MIC determinations. After 24 and 48 h of culture at 35°C, the optical density was spectrophotometrically determined using an E-max Microplate Reader at a wavelength of 600 nm. The assays were repeated three times for each strain. In one repetition, each concentration of the compound was tested in 4 replicates (wells). The results presented are therefore the average of 12 individual measurements. The data were normalized according to the procedure described for MIC determinations. Basic statistics, one-way analysis of variance (ANOVA), and a post-hoc Tukey test, were performed using StatSoft Statistica 12.5 software.

The minimal fungicidal concentration (MFC) was determined with the plate serial dilution method. Fungal cultures treated for 48 h with the concentrations of the C1 compound ranging from 16 to 256 μg/ml and prepared as described for the MIC determinations were serially diluted and aliquots of 20 μl were inoculated onto the Sabouraud dextrose agar plates. Samples treated with a concentration of the compound at which no colony growth was expected were inoculated on the plates without dilution. After 48 h of incubation at 35°C or 27°C for the yeasts and molds, respectively, the number of colonies was counted. The concentrations at which no colonies or only 1–2 colonies were grown were taken as the MFC values. The MFC assays were repeated three times for each strain/species, and duplicate samples were included in each repeat. The results presented therefore come from 6 individual readings. Two-fold dilutions of the compound were tested (0.5; 1; 2; 4; 8; 16; 32; 64, 128, 256 μg/ml, and additionally 96 μg/ml); hence, the read MFC values were identical in each repetition for a given strain. Therefore, it was not necessary to calculate the average values.

### Morphological studies

Morphological observations and measurements were carried out using standard strains of *C*. *albicans* (NCPF 3153) and *C*. *parapsilosis* (ATCC 22019). For measurements of the cell size, the cultures were treated with the C1 compound at the concentrations of ¼ MIC_70_ (8 μg/ml for *C*. *parapsilosis* and 16 μg/ml for *C*. *albicans*) in the culture medium as described for the MIC determinations at the initial inoculum density of 5 x 10^3^–1 x 10^4^ cells/ml. At the determined time points (24, 48, and 72 h), cell samples were taken, placed on microscope slides, covered with a cover glass, and observed under a bright field optical microscope. A series of images of each slide were taken at a fixed magnification using the Canon A 640 digital camera. The measurements of the short and the long diameter of 200 individual cells in the images were made for each sample and the cell area in μm^2^ (cell size) was estimated according to the formula for the ellipse area (major radius x minor radius x 3.14) using Microsoft Excel software (MS Excel 2010, Microsoft Corporation). The ratio of the long/short cell diameter was also calculated to determine changes in the cell shape. Basic statistics, one-way ANOVA, and a post-hoc Tukey test were performed using StatSoft Statistica 12.5 software. The basic statistics, including the median, 25–75% range of data, and non-outlier and outlier range of data were presented on box and whisker plots or on scatter plots prepared using StatSoft Statistica 12.5 software. Statistically significant differences between the averages of the treated groups in relation to the untreated control were marked with asterisks on the plots.

For induction of hypha formation and observation of morphological changes, *Candida* cells were cultured in RPMI 1640 medium without glucose enrichment with addition of 20% bovine serum, at 37°C. Control cells and cells treated with the C1 compound at the concentrations of 0.5–8 μg/ml were cultured in 96-well plates for 48 h. After that time, the cells were stained with Calcofluor White (as described below), and slides from each C1 concentration were prepared and observed under a fluorescence and phase-contrast microscope. A series of images was taken of each slide. The number of cells displaying each morphotype was scored, and the percentage of each morphotype was calculated.

### Supravital cell staining with fluorochromes

For chitin detection, the cells were stained with Calcofluor White (Sigma-Aldrich, 18909). The stock solution containing Calcofluor White M2R (1 g/l) and Evans Blue (0.5 g/l) was added to the culture suspension in a volume ratio of 1:1. After 5 min of staining in the dark, the cells were centrifuged, washed in PBS buffer, pH 7.4, and placed on the microscopic slides. Freshly prepared slides were examined under a Nikon Labophot 2 fluorescence microscope equipped with a 380–420 nm band-pass blue excitation filter block (V-2A, Nikon), a 430 nm dichromatic mirror, and a 450 nm long-pass barrier filter.

To stain β(1→3) glucan, the cells were stained with a 0.005% Aniline Blue solution (Sigma-Aldrich, 415049) in 1 M glycine, pH 9.5, for 5 min and observed in a drop of the solution using a Nikon Labophot 2 fluorescence microscope equipped with a 330–380 nm band-pass blue excitation filter block (UV-2A, Nikon), a 400 nm dichromatic mirror, and a 420 nm long-pass barrier filter.

Intracellular compartments with low pH were visualized using Acridine Orange, i.e. a metachromatic fluorescent cationic dye, which permeates the cell membrane, intercalates DNA and RNA, and additionally differentiates cell compartments with different pH. The acidic compartments fluoresce bright red, and thus the volume of acidic cellular compartments can be quantified using acridine orange staining [19, 20]. The cells were stained with a water solution of Acridine Orange hydrochloride hydrate (Sigma-Aldrich, 318337) at a final concentration of 1 μg/ml for 20 min. Next, the cells were centrifuged, washed in water, and placed on microscope slides. The microscope was equipped with a 450–490 nm band-pass blue excitation filter block (B-2A, Nikon), a 500 nm dichromatic mirror, and a 515 nm long-pass barrier filter. Images were captured on a Nikon Labophot 2 fluorescence microscope equipped with a Canon Power Shot A 640 digital camera. *Candida* cells treated with the C1 compound were also checked for fluorescence without any additional fluorochrome staining to exclude background fluorescence that might confound the interpretation of the fluorochrome staining data.

### Osmotic resistance test

To test the osmotic resistance of the C1-treated cells, fungal cultures of standard *C*. *albicans* and *C*. *parapsilosis* strains at the initial inoculum density of 5 x 10^3^–1 x 10^4^ cells/ml were treated with the C1 compound at the concentration of ¼ MIC_70_ for 48 h. After the treatment, the cells were centrifuged, washed in PBS buffer, pH 7.4, centrifuged again, and divided into two groups of samples. The first group was resuspended in RPMI 1640 culture medium and the second group in pure water, and incubated for 30 min at 35°C. Control cells (not treated with C1) were prepared in the same way. Following incubation in the culture medium or in water, the cells were centrifuged and slides were prepared. Freshly prepared slides were examined under an optical bright field microscope and a series of images of each slide was taken at a fixed magnification using a Canon A 640 digital camera. Measurements of the short and the long diameter of 200 individual cells in the images were made for each sample and the cell area in μm^2^ (cell size) was estimated according to the formula for the ellipse area (major radius x minor radius x 3.14) using Microsoft Excel software (MS Excel 2010, Microsoft Corporation). Basic statistics, one-way ANOVA, and a post-hoc Tukey test were performed using StatSoft Statistica 12.5 software. The scatter plots presenting the distribution of the data in particular samples were prepared using StatSoft Statistica 12.5 software. Statistically significant differences between the averages of the treated groups in relation to the untreated control were marked with asterisks on the plots.

### Interaction of C1 with Calcofluor White

Calcofluor White is a compound that interferes in the polymerization of cell wall molecules. To study the interaction of C1 and Calcofluor White, cultures of standard strains of *C*. *albicans* and *C*. *parapsilosis* were prepared on 96-well plates according to the procedure described for MIC determinations. In the successive rows of the wells, the fungal cells were treated with Calcofluor White (at the concentrations of 10 and 28 μg/ml), with the C1 compound (at the concentrations of 4, 8, 16, and 32 μg/ml) individually, or with a combination of these substances. After 48 h incubation at 35°C, the OD_600_ was measured using the E-max Microplate Reader. The assays were repeated three times and each sample was tested in 4 replicates (wells) in one repetition. The results presented are therefore the average of 12 individual measurements. Basic statistics, one-way ANOVA, and a post-hoc Tukey test were performed in the StatSoft Statistica 12.5 software. Statistically significant differences between the treatment groups were marked with asterisks on the plots.

### Scanning electron microscopy (SEM)

Cells were cultured in RPMI 1640 medium, buffered to pH 7.0 with 0.165 mol/l MOPS, and supplemented with 2% of glucose in Eppendorf tubes for 24 h with shaking at 30°C starting at an initial inoculum density of 5 x 10^4^ cells/ml. There were two groups of samples: the control (no drug added) and samples treated with the C1 compound at a concentration of 32 μg/ml. The cells were harvested by centrifugation at 1000 rpm for 3 min at 4°C and fixed for 24 h in 4°C in a solution containing 4% glutaraldehyde (G5882 Sigma-Aldrich), 0.5% formaldehyde (18814–10 Polyscience), 5 mM CaCl_2_, and 0.5 M sorbitol (S1876 Sigma-Aldrich) in 0.1 M cacodylate buffer pH 7.2 (prepared from sodium cacodylate trihydrate 01131–25 Polyscience). Next, the cells were rinsed twice in the cacodylate buffer pH 7.2 and postfixed in a 1% osmium tetroxide solution (75632 Sigma-Aldrich) with 0.5 M sorbitol in 0.1 M cacodylate buffer pH 7.2 for 2 h in 4°C. After washing twice in the cacodylate buffer, cells were dehydrated in a series of ethanol solutions (30%, 50%, 70%) and next in acetone solutions (70%, 90%, 99%) for 15 min. Dehydratation of the cells was continued in anhydrous acetone (2 x 30 min) and next in a desiccator with CaCl_2_ for 24 h. The dried samples were mounted on specimen stubs using a double-sided adhesive tape and coated with gold in a vacuum sputter coating system. The coated samples were viewed under a VEGA 3 TESCAN scanning microscope at 30 kV. The whole procedure was repeated 3 times. Representative photographs from the control and C1-treated cells are presented.

### The spheroplast lysis assay

Cells were cultured in YPD broth at the initial inoculum density of 5 x 10^4^ cells/ml, for 24 h with shaking at 30ºC. There were two groups of samples: the control (no drug added) and samples treated with the C1 compound at the concentration of 32 μg/ml. The rate of spheroplasts lysis under the enzymatic digestion of the cell walls in hypotonic conditions was assessed according to the method described by Ovalle et al. [21]. After 24 h of culture, cells at the early stationary phase were harvested by centrifugation at 1000 x g for 5 min and washed three times in sterile deionized water. The harvested cells were suspended in TE buffer (50 mM Tris-HCl, 150 mM NaCl, 5 mM EDTA; pH 7.5) to obtain optical density between 0.6 and 0.7. Zymolyase^TM^ 100 T (AMS Biotechnology), i.e. lyophilized powder containing a mixture of protease and β-glucanase, was dissolved in a 1:1 glycerol/water solution to a concentration of 20 mg/ml and stored at -70°C. The working solution was prepared by diluting the stock solution 1:10 with TE buffer. A 100 μl aliquot of the Zymolyase working solution was added to 2 ml of the cell suspension in TE buffer (optical density between 0.6 and 0.7), vortexed, and kept in a thermostat at 25°C. The samples were vortexed for 30 s before each OD determination (600 nm, 10 min intervals) in the spectrophotometer (Agilent Cary 60 UV-Vis). The experiment was repeated three times with two replicates (n = 6). Basic statistics, multivariate analysis of variance (MANOVA), and a post-hoc Tukey (HSD) test were performed using StatSoft Statistica 12.5 software.

### Quantification of ergosterol

Determination of the ergosterol content in the C1-treated cells, in comparison with the control, was performed using the *C*. *albicans* standard strain (NCPF 3153), which is susceptible to FLC. To confirm the specificity of the ergosterol quantification method, the amount of this compound was additionally measured in cells treated with FLC, which inhibits ergosterol biosynthesis, and with AmB, which does not affect the synthesis of this compound. Cells at the initial inoculum density of 5 x 10^3^–1 x 10^4^ cells/ml were cultured in RPMI 1640 medium, without phenol red and sodium bicarbonate (Sigma-Aldrich, catalog number: R 8755), buffered to pH 7.0 with 0.165 mol/l MOPS and enriched with 2% glucose, with addition of the C1 compound at the concentrations of ½ and ¼ MIC_70_, FLC at the concentration of ½ MIC_70_, or AmB at the concentration of ½ MIC_100_ (MIC values are indicated in Table A in S1 File), in 2 ml Eppendorf tubes for 24 h with shaking at 35°C. Cells were harvested by centrifugation at 1,700 x g for 5 min at 4°C and washed once with sterile distilled water. The supernatant was carefully drained by setting the tubes at an angle on filter paper. The cell pellet from a few Eppendorf tubes in each treatment was collected to obtain a net wet weight of yeast equal to 200 mg. Total intracellular sterols were extracted and spectrophotometrically measured as described by Breivik and Owades [22] with modifications [23]. The cell pellet was mixed with 3 ml of a 25% potassium hydroxide ethanolic solution (25 g KOH added to 35 ml of sterile deionized water and brought to 100 ml with 100% ethanol, the solution was freshly prepared daily), transferred to borosilicate glass tubes with screw caps, vortex mixed for 1 min, and incubated in a water bath at 85°C for 1.5 h. After cooling to room temperature, total sterols were extracted by adding 3 ml of *n*-heptane and 1 ml of sterile deionized water and vigorous mixing on a vortex for 3 min. The blank solution was prepared by mixing 3 ml of a 25% potassium hydroxide ethanolic solution, 3 ml of *n*-heptane, and 1 ml of sterile deionized water. The test and blank tubes were left for 30 min to allow the *n*-heptane layer to clarify. After this time, the *n*-heptane layer was carefully collected and diluted fivefold with absolute ethanol. Absorbance was measured at 281.5 and 230 nm against a similarly diluted blank using a Varian Cary 50 UV-Vis Spectrophotometer. The reading at 281.5 nm is the sum of the absorbances of ergosterol and the late sterol intermediate 24(28)-dehydroergosterol (DHE), while the reading at 230 nm is the absorbance of DHE. The content of ergosterol was calculated as a percentage of the wet weight of the cell pellet according to the equations: % ergosterol + % DHE = [(absorbance at 281.5 nm/290) x dilution]/pellet weight, % DHE = [(absorbance at 230 nm/518) x dilution]/pellet weight and % ergosterol = (% ergosterol + % DHE)—% DHE, where 290 and 518 are the *E* values (in percentages per centimeter) determined for crystalline ergosterol and DHE, respectively.

This experiment was repeated three times with one replication (n = 3). The results are presented as the percentage of ergosterol content in the treatment samples relative to the control sample. The percentage of the control was calculated according to the following equation: (% of ergosterol in wet weight of the treated sample) / (% of ergosterol in wet weight of the untreated sample) x 100. Next, the average value of the percentage of the ergosterol content of the treated samples in relation to the control was calculated. Basic statistics, one-way ANOVA, and a post-hoc Tukey test were performed using StatSoft Statistica 12.5 software. A statistically significant difference between the treated samples and the control sample is marked with an asterisk on the plot.

### Cell wall isolation

The cells were cultured at the initial inoculum density of 5 x 10^4^ cells/ml in RPMI 1640 medium pH 7.0 with 0.165 mol/l MOPS and supplemented with 2% of glucose in Eppendorf tubes for 24 h with shaking at 30°C. There were two groups of samples: the control (no drug added) and samples treated with the C1 compound at the concentration of 32 μg/ml. The cell walls were isolated with the method described by Pitarch et al. [24]. Briefly, cells were harvested by centrifugation at 4500 x g for 5 min at 4°C and washed three times with sterile ice-cold water and then twice with an ice-cold lysis buffer (10 mM Tris-HCl, pH 7.4 with a protease inhibitor—1 mM phenylmethylsulfonyl fluoride (PMSF, P7626 Sigma-Aldrich). Cells resuspended in ice-cold lysis buffer (10^8^ cells/μl) were frozen with liquid nitrogen and disintegrated in an alabaster mortar by grinding with a pestle. The cycle of freezing and grinding was carried out until complete cell breakage, verified under a phase-contrast microscope and *a posteriori* by the lack of colony growth on YPD plates. To remove noncovalently bound proteins and intracellular contaminants, a multi-stage washing procedure was performed. The isolated cell walls were resuspended in 10 ml of the first washing solution (1 mM PMSF in ice-cold water) and centrifuged 10 min at 1000 x g at 4°C. The supernatant was carefully discarded. This step was repeated three more times. A similar procedure was performed with the second (5% NaCl, 1 mM PMSF), third (2% NaCl, 1 mM PMSF) and fourth (1% NaCl, 1 mM PMSF) washing solutions. The isolated cell walls were washed three times with ice-cold sterile water and harvested by centrifugation. Aliquots (100 μl) of the isolated cell walls were poured into freezing tubes and stored in liquid nitrogen until use.

### ATR-FTIR spectroscopy

Attenuated total reflection Fourier transform infrared spectroscopy (ATR-FTIR) was used to study the influence of C1 on *C*. *albicans* cell wall material. A 20 μl aliquot of the isolated cell walls was sampled on the ZnSe crystal and dried under a N_2_ stream. The ATR-FTIR spectra in the range of 4000–900 cm^-1^ were measured for the cell walls isolated from *C*. *albicans* control and C1–treated cells using a FTIR VERTEX 70 (Bruker Optik GmbH, Germany) spectrometer. Spectral resolution was set at 2 cm^-1^, sampling 16 scans per sample, and the MCT detector was used. The software Grams/AI 9.1 Spectroscopy Software (Thermo Fisher Scientific Inc.) was used to carry out baseline correction, vector normalization, and calculation of the reverse second derivatives of spectral values. The experiment was repeated three times and duplicate samples were included in each repeat. Three spectra were measured from each replicate sample. Representative spectra and reverse second derivatives of spectral values of the walls isolated from control cells and those treated with 32 μg/ml of C1 are presented.

### Evaluation of cytotoxic activity

The cytotoxicity of the C1 compound was determined *in vitro* against normal human dermal fibroblasts (NHDF, Lonza, CC-2511, Bazel, Switzerland). The cells were stored in liquid nitrogen in a tissue bank, thawed at 37°C before the experiment, transferred to culture bottles for adherent cultures (Nunclon®) containing Dulbecco’s modified Eagle medium nutrient mixture F-12 HAM (Sigma, D8062), supplemented with 10% heat-inactivated fetal bovine serum and 10 μg/ml of gentamycin, and cultured in an incubator with humidified atmosphere saturated with 5% CO_2_ at 37°C for 48 h. The cell suspension obtained by detachment with a 0.25% trypsin-EDTA solution was diluted with the medium described above, pipetted to 96-well plates for adherent cultures (Nunclon®) at the density of 6,500 cells in 200 μl of the medium per well, and cultured for 24 h. Next, the culture medium was gently aspired and prewarmed medium supplemented with the C1 compound was added at the concentrations of 4–512 μg/ml. The cells were further cultured for 48 h, after which the cell viability was determined with the *In Vitro* Toxicology Assay Kit, MTT based (Sigma-Aldrich, TOX 1), according to the manufacturer’s instructions. The amount of formazan formed was determined spectrophotometrically using an E-max Microplate Reader at a wavelength of 570 nm. Based on these analyses, the concentration leading to 50% inhibition of cell viability (IC_50_) by the studied compound was estimated. The assays were repeated three times with 8 replicates (wells) for each concentration. The results presented are therefore the average of 24 individual measurements. They are presented as a percentage of the viability of the treated cells in relation to the control cells. The percentage of the viability of the treated cells was calculated according to the following equation: (absorbance at 570 nm of the treated sample) / (average absorbance at 570 nm of the untreated sample) x 100. Next, the average value of the percentage of the cell viability in the treated samples in relation to the control was calculated. Basic statistics, one-way ANOVA, and a post-hoc Tukey test were performed using StatSoft Statistica 12.5 software. Statistically significant differences between the treated samples and the control sample were marked with asterisks on the plot.

## Results

### The C1 compound exhibits antifungal activity against various fungal species

**Fig 2:**
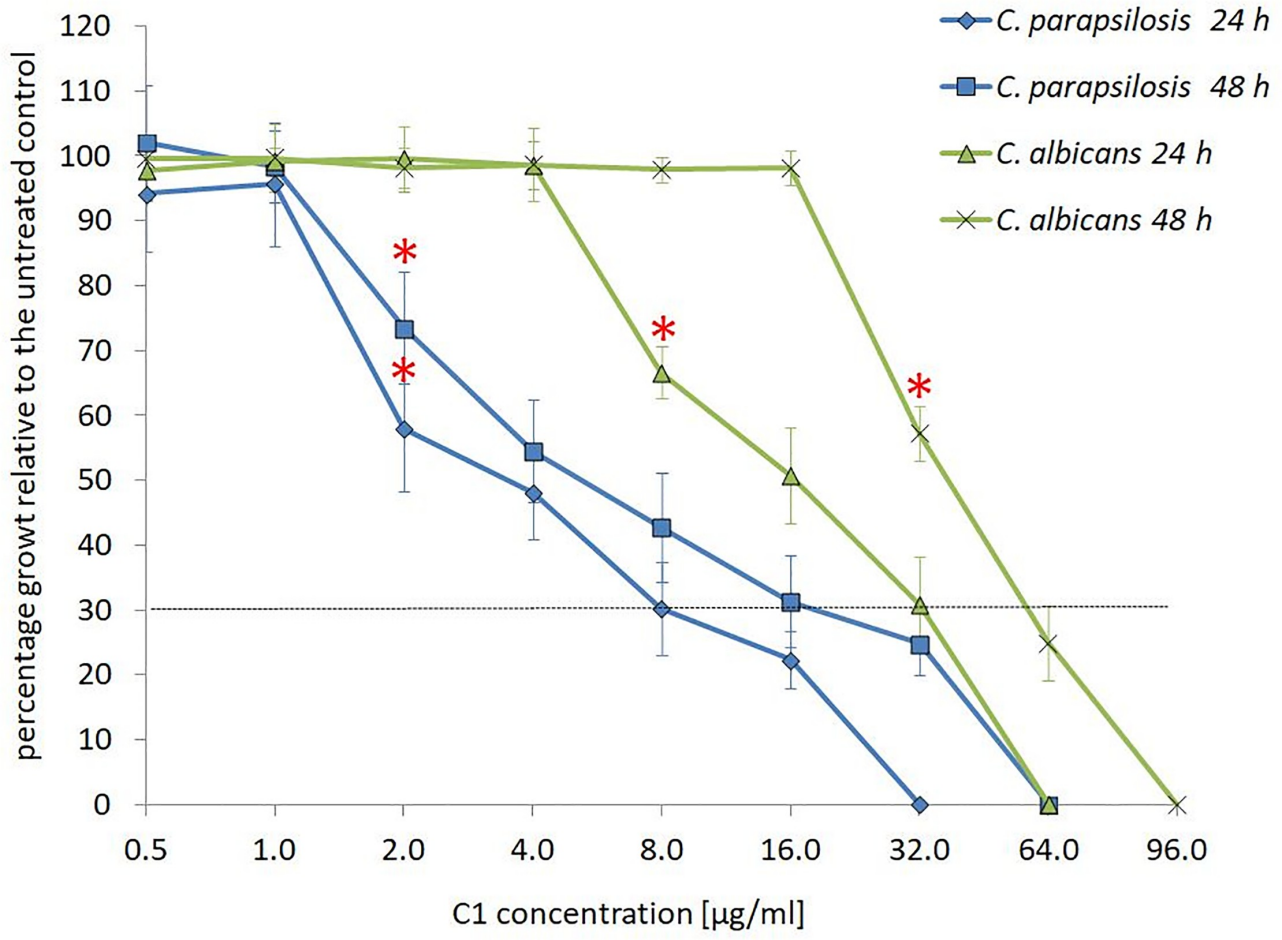
Percentage of *C*. *albicans* NCPF 3153 and *C*. *parapsilosis* ATCC 22019 growth, measured as the OD_600_, induced by the C1 compound in relation to the control. Yeasts were grown in 96-well plates according to the procedure described for MIC determinations. After 24 and 48 h of culture at 35°C, the optical density was spectrophotometrically determined at a wavelength of 600 nm. The assays were repeated three times for each strain. In one repetition, each concentration of the compound was tested in 4 replicates (n = 12). The percentage of treated samples growth in relation to the control was calculated according to the following equation: (OD_600_ of the treated sample) / (OD_600_ of the untreated sample) x 100. The average percentage values are given along with the standard deviation. Raw data can be found in Tables B and C in S1 File. *—the lowest concentration at which a statistically significant decrease in OD in comparison with the control was noted, as determined by one-way ANOVA and a post-hoc Tukey test. The dotted line indicates 30% growth relative to the control.

To determine the MIC of C1 against fungal species and strains, the broth microdilution method was employed according to the standard methodological guidelines recommended by the Clinical and Laboratory Standards Institute (CLSI): document M27-A3 and M38-A2 for testing the susceptibility of yeasts and moulds, respectively. MFC was studied using the plate serial dilution method. The results of these investigations showed that the C1 compound has antifungal activity against various types of standard strains and clinical isolates. Depending on the concentration, it exhibited fungistatic or fungicidal activity (Table 1). The values of the minimal inhibitory concentration inhibiting fungal growth to 100% (MIC_100_) obtained after 48 h of culture varied depending on the strain in the range of 8–96 μg/ml, while the values of the minimal inhibitory concentration inhibiting fungal growth to 70% (MIC_70_) were lower and ranged from 4 to 64 μg/ml. An advantage of this compound is the fact that it is possible to determine its concentration that completely inhibits the proliferation of the pathogenic fungi and the minimal fungicidal concentration (MFC) at which the growth of colonies is completely eradicated. The MFC values determined for this compound ranged from 32 to 128 μg/ml, and the MFC/MIC_100_ ratio for the tested pathogens was in all cases < 4, indicating that the C1 compound has not only fungistatic but also fungicidal activity. Furthermore, the analyses have shown that the C1 compound has a wide range of antifungal activity against *C*. *albicans* and non-*albicans* species from the *Candida* genus and against molds, such as *Aspergillus* and *Trichophyton*. It also exhibited fungicidal activity against clinical isolates resistant to azoles, with similar MIC and MFC values to those against strains that are sensitive to these antifungals (see Table A in S1 File). Detailed studies of the antifungal activity of the C1 compound were performed on standard *C*. *albicans* and *C*. *parapsilosis* strains by observing the dynamics of growth inhibition induced by the C1 compound in the two *Candida* species after 24 and 48 h of treatment (Fig 2). These experiments showed 70% inhibition of *C*. *parapsilosis* and *C*. *albicans* growth at C1 doses that were by half or two third, respectively, lower than the determined MIC_100_.

**Table 1:** Antifungal activity of the C1 compound against standard strains and clinical isolates of pathogenic fungi.

Strain	MIC_70_ [μg/ml]	MIC_100_ [μg/ml]	MFC [μg/ml]	MFC/MIC_100_
*Candida albicans* NCPF 3153	64	96	96	1
*Candida parapsilosis* ATCC 22019	32	64	64	1
*Trichophyton rubrum* ATCC 28188	8	32	32	1
*Aspergillus niger* ATCC 16888	32	64	128	2
*Rhodotorula mucilaginosa* ATCC 22273	16	32	64	2
*Candida albicans* isolate 102	16	32	64	2
*Candida krusei* isolate 93	16	64	128	2
*Candida dubliniensis* isolate 176	4	8	16	2
*Candida glabrata* isolate 124	32	96	128	1,3
*Candida krusei* isolate 103	64	96	128	1,3
*Candida tropicalis* isolate 175	64	96	128	1,3

**MIC**_**70**_—minimal inhibitory concentration reducing pathogen growth by 70% after 48 h of culture

**MIC**_**100**_—minimal inhibitory concentration completely reducing pathogen growth after 48 h of culture

**MFC**—minimal fungicidal concentration completely eradicating the colony growth

### Morphological analysis revealed that the C1 compound causes phenotypic changes in *Candida* species

**Fig 3:**
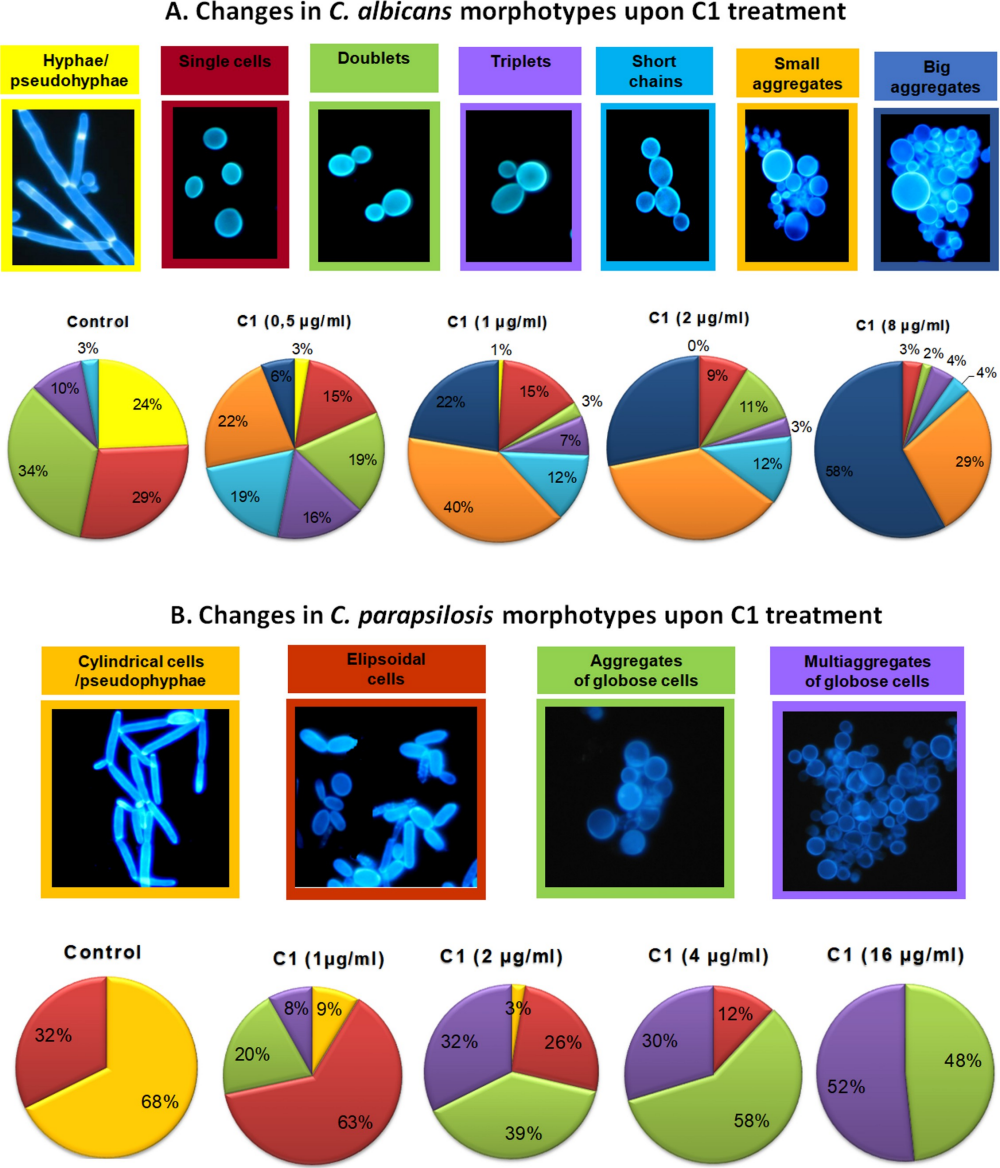
**Changes in the morphotypes of *C*. *albicans* NCPF 3153 (A) and *C*. *parapsilosis* ATCC 22019 (B) upon C1 treatment.** *Candida* cells were cultured in RPMI 1640 medium, without glucose enrichment, with addition of 20% bovine serum, at 37°C for 48 h. Control cells were cultured without the drug, and the C1 compound was added to the treatment groups at concentrations ranging from of 0.5–16 μg/ml as indicated. Following treatment, cells were stained with Calcofluor White and images were acquired using a fluorescence microscope. The number of cells displaying each morphotype was recorded and the percentage share was calculated. The total number of cells counted for each sample ranged from 79–300 (see Tables D and E in S1 File for raw data).

Observations of *Candida* cells cultured in presence of the C1 compound by microscopy showed that cells exhibited characteristic morphological disturbances occurring at doses significantly lower than the determined MIC values. A detailed analysis of morphological aberrations was performed for the *C*. *albicans* NCPF 3153 and *C*. *parapsilosis* ATCC 22019 standard strains which differed in cell shape and ability to produce specific morphotypes. Furthermore, these strains vary in their sensitivity to the most effective antifungal agent from the polyene group (AmB); *C*. *albicans* is sensitive to AmB (the MIC is equal to 0.5 μg/ml; Table A in S1 File), while *C*. *parapsilosis* is not very sensitive to this antifungal (the MIC is equal to 2 μg/ml; Table A in S1 File). To investigate the effect of the C1 compound on the formation of specific morphotypes, the *Candida* strains were cultured in MOPS-buffered RPMI 1640 medium with the addition of 2% glucose and 20% bovine serum, at 37°C, for 48 h. In this medium, *C*. *albicans*, which is a polymorphic fungus, produced different morphological forms, including the yeast form (blastospores), true hyphae, and pseudohyphae. In the control population (no drug added), the hyphal and pseudohyphal morphotypes accounted for approx. 24% of *C*. *albicans* cells; 29% were blastospores in the form of rounded single cells, 34% were budding yeast (doublets), approx. 10% were three yeast cells combined together (triplets), and only 3% were short chains of joined cells (Fig 3A). The addition of the C1 compound at a concentration of 0.5 and 1 μg/ml resulted in a reduction of the percentage of hyphae and pseudohyphae produced, down to 3 and 1%, respectively. A C1 concentration of 2 μg/ml completely inhibited formation of these morphotypes. Another distinctive phenotypic change occurring under the influence of increasing concentrations of the C1 compound was the reduction in the percentage of single yeast forms and budding doublet yeasts with a corresponding increase in the formation of multicellular aggregates. Multicellular aggregates appeared at a C1 concentration as low as 0.5 μg/ml, at which small aggregates represented about 22% and big multicellular aggregates accounted for about 6% of all cells in the culture. With an increase in the C1 compound concentration, the number of the big cellular aggregates increased, reaching 44% and 58% of all cells in the culture at C1 concentrations of 2 and 8 μg/ml, respectively. The non-aggregated morphological forms at the C1 concentrations of 2 and 8 μg/ml were reduced to a very low level (Fig 3A).

The *C*. *parapsilosis* cells growing in the control medium had a cylindrical shape and most were arranged in long chains forming pseudohyphal (Fig 3B). The cells treated with C1 exhibited aberrant morphology, which manifested as a change in the cell shape from cylindrical to rounded, as well as a tendency to flocculate. These phenotypic changes were observed at C1 concentrations as low as 0.5 μg/ml. The percentage of aggregates, both small and big, increased with an increase in C1 concentration. At a concentration of 2 μg/ml, cylindrical cells constituted only 3% and the total share of aggregates of different sizes was about 71%. In the culture treated with C1 at a concentration of 8 μg/ml, there were no cylindrical cells at all. Free ellipsoid cells constituted only 3% of the cell population, and aggregates of rounded cells were a vast majority (Fig 3B).

**Fig 4:**
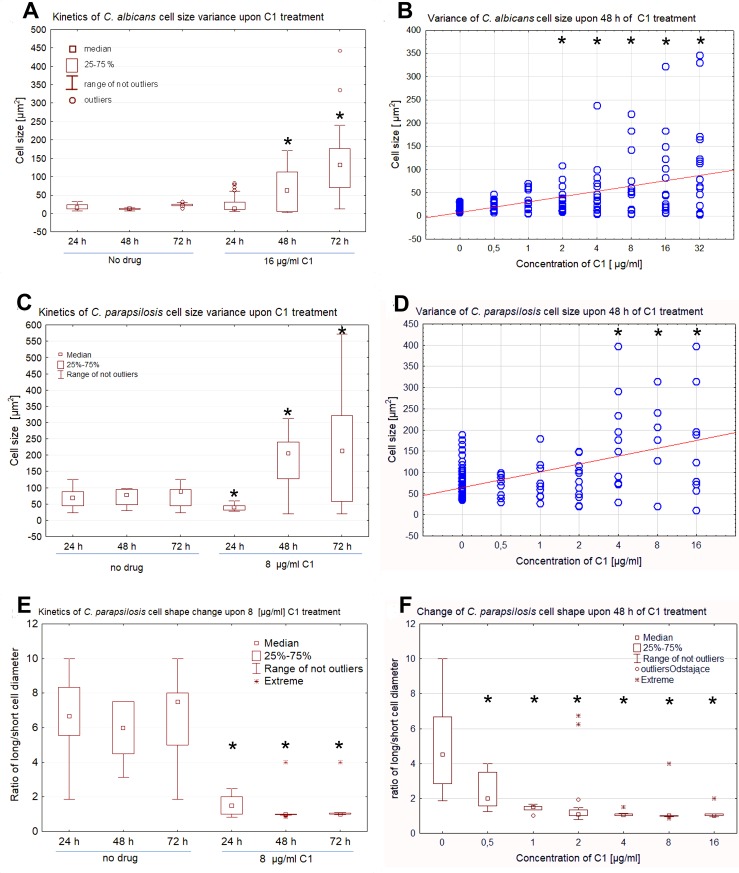
Cell size and shape changes upon C1 treatment in *C*. *albicans* NCPF 3153 and *C*. *parapsilosis* ATCC 22019. Yeast cells were cultured in RPMI 1640 medium, with 2% of glucose at 35°C, with different concentrations of C1. A series of images was taken for each condition under a bright field microscope at a fixed magnification. Two diameters of 200 individual consecutive cells in the microphotographs were measured in each slide and used to estimate cell size. Raw data are presented in Tables F-I in S1 File. The kinetics of the cell size changes during 3 d culture in the presence of the C1 compound at the concentration of ¼ MIC_70_ is shown in panel **A** for *C*. *albicans* and in panel **C** for *C*. *parapsilosis*. The variance in the size of cells treated with the range of the C1 concentrations for 48 h is shown in panel **B** for *C*. *albicans* and in panel **D** for *C*. *parapsilosis*. Changes in the *C*. *parapsilosis* cell shape expressed as the ratio of the long/short cell diameter are shown as the kinetics in the 3 d culture in the presence of the C1 compound at the concentration of ¼ MIC_70_ (panel **E)** and for cells treated with the range of the C1 concentrations for 48 h (panel **F).** The median, 25–75% range of data, and non-outlier and outlier range of data are presented as box and whisker plots or scatter plots. *—statistically significant differences in comparison to the control (*p*<0.05) as determined by one-way ANOVA and a post-hoc Tukey test.

It was observed that the treatment of *Candida* cells with C1 resulted in the formation of abnormally large cells, in addition to the altered rounded shape of the cells. The kinetics of the cell size changes was analyzed after 24, 48, and 72 h of culture upon C1 treatment at the concentration ¼ MIC_70_. The morphometric analysis performed for both *Candida* species revealed that the size of the cells in the control cultures (without the addition of the drug) did not change statistically significantly on the particular days of their growth. In the case of *C*. *albicans*, the average size of the control cells was 17 ± 6.6 μm^2^ after 24 h, 11.7 ± 2.6 μm^2^ after 48 h, and 22.2 ± 6 μm^2^ after 72 h of culture (Fig 4A). There was also no statistically significant increase in the size of the cells 24 h after the start of the culture with the addition of 16 μg/ml of C1 (23.8 ± 20.5 μm^2^), although a greater variation in the cell size was observed in this group. However, after 48 and 72 h of culture upon the C1 treatment, an increase in the size of many cells was observed, giving an average score of 65.8 ± 55.9 μm^2^ after 48 h and 156.7 ± 103.7 μm^2^ after 72 h (a statistically significant increase in comparison to the control, *p*<0.05). Due to the high standard deviation, the calculated average cell size does not reflect the actual state, because under the influence of C1, very small cells with a size ranging between 8–12 μm^2^ (probably degenerated cells or empty cell walls) and very large cells with a size of 200–400 μm^2^ appeared, which were not observed in the control conditions. The cell size distribution for *C*. *albicans* in the following days after the application of 16 μg/ml C1 is shown in a statistical plot (Fig 4A). Additional analysis was performed to identify the lowest concentration of the C1 compound at which changes in the cell size and shape would occur after 48 h of the treatment. In the case of *C*. *albicans*, statistically significant changes in the cell size were already observed at the C1 concentration of 2 μg/ml. The average size of the cells increased with the increasing C1 concentration (Fig 4B).

In the case of *C*. *parapsilosis*, the size of the control cells was 68.1 ± 29.6 μm^2^ after 24 h, 88.4 ± 25.4 μm^2^ after 48 h, and of 72.6 ± 31.5 μm^2^ after 72 h of culture (Fig 4C), and the differences were not statistically significant. The treatment with the C1 compound at the concentration of 8 μg/ml for 24 h resulted in reduction of the mean cell size to 40.9 ± 9.9 μm^2^, while at the longer exposure time the cell size grew rapidly and reached 182.9 ± 97.9 μm^2^ after 48 h and 211.4 ± 173.5 μm^2^ after 72 h (statistically significant differences in comparison to the control, *p*<0.05). Also, in the case of this species, after a longer time of exposure to C1, a large diversity of cell sizes was observed, i.e. the presence of very small and very large cells. In a subsequent experiment, we determined the lowest C1 concentration at which changes in the *C*. *parapsilosis* cell size would occur after 48 h of culture. The results showed a statistically significant increase in the cell size at the concentration of 4 μg/ml (Fig 4D).

In *C*. *parapsilosis*, a change in the shape of the cells from cylindrical to rounded was observed, which is numerically shown by calculation of the ratio of the long-to-short cell diameter (Fig 4E). In the control group, the long cell diameter was on average 6 times longer than the short one, but this ratio fell down to about 1 after 24 h of culture in the presence of C1 and remained at a similar level after the prolonged culture, which indicates loss of the characteristic cylindrical shape of the cells and transition to a rounded form. The change in the long-to-short cell diameter ratio (change in the cell shape) was statistically significant already at the C1 concentration of 0.5 μg/ml (Fig 4F).

### C1 affects fungal cell wall integrity

**Fig 5:**
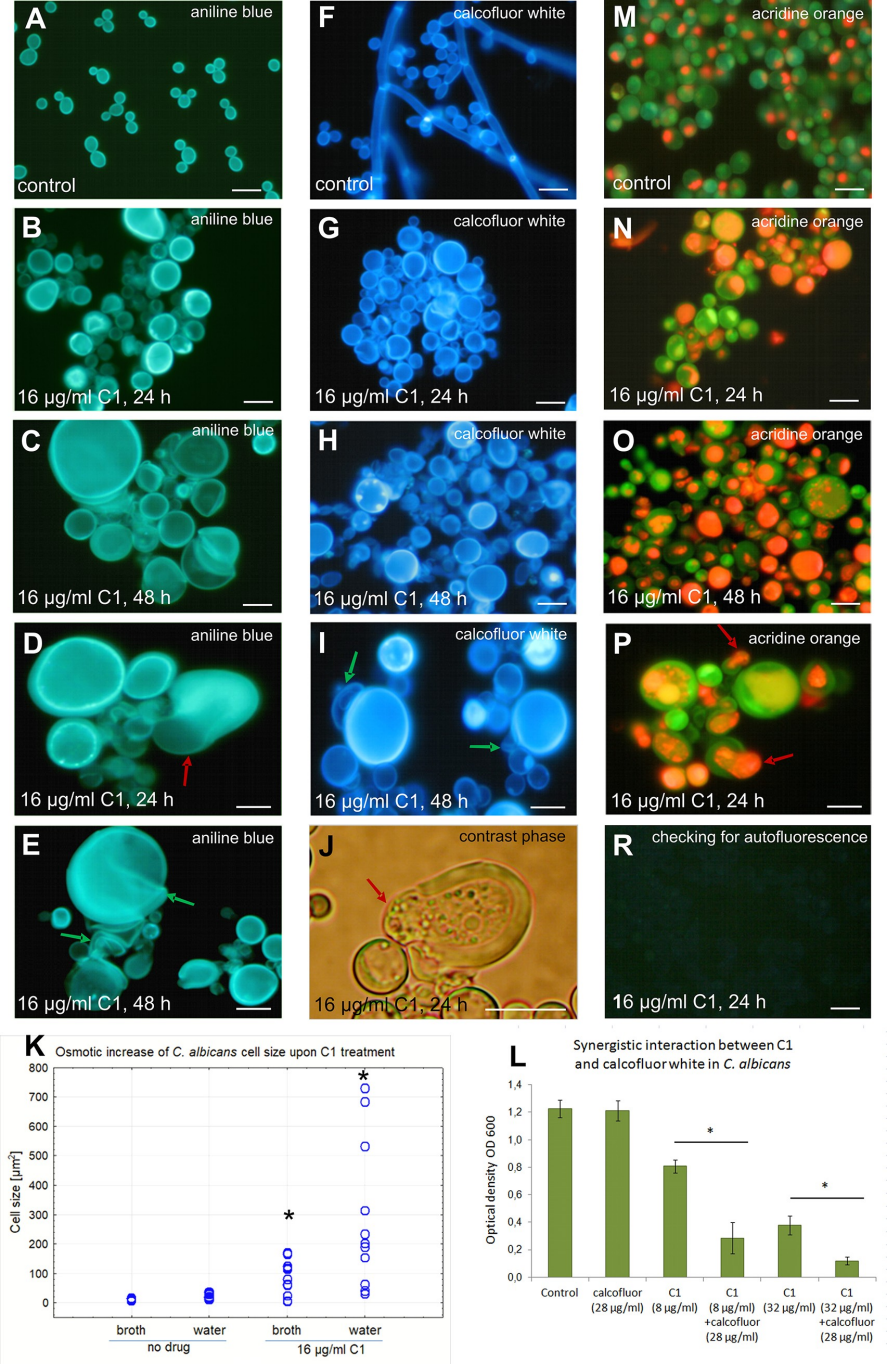
Abnormalities of cell walls and intracellular compartments of *C*. *albicans* NCPF 3153 upon C1 treatment. **A-E.** Aniline Blue staining for β(1→3) glucan. **F-I.** Calcofluor White staining for chitin. **J**. Phase contrast microscope image. **M-P**. Acridine Orange staining for acidic compartments. **R.** Unstained, C1-treated cells (control for autofluorescence). Red arrows–protoplasts being pushed out by internal turgor pressure; green arrows—defective cell walls. Scale bar = 10 μm. **K.** Distribution of the size (cell area in μm^2^) of cells pretreated with the C1 compound for 48 h then exposed to osmotic stress (water for 30 min) in comparison with drug non-treated cells (control cells). Measurements of 200 cells were made for each sample and are presented as scatter plots. Statistically significant differences between the averages of treated groups in relation to the untreated control are marked with asterisks on the plots and were determined by one-way ANOVA and a post-hoc Tukey test. Raw data are shown in Table J in S1 File. **L.** Growth of cells treated with Calcofluor White and C1 expressed as the final OD_600_ obtained after 48 h at 35°C. The assays were repeated three times with 4 replicates (wells). The results presented are therefore the average of 12 individual measurements. Raw data are provided in Table L in S1 File. *–statistically significant difference between the treatment groups (*p*<0.05) as determined by one-way ANOVA and a post-hoc Tukey test.

**Fig 6:**
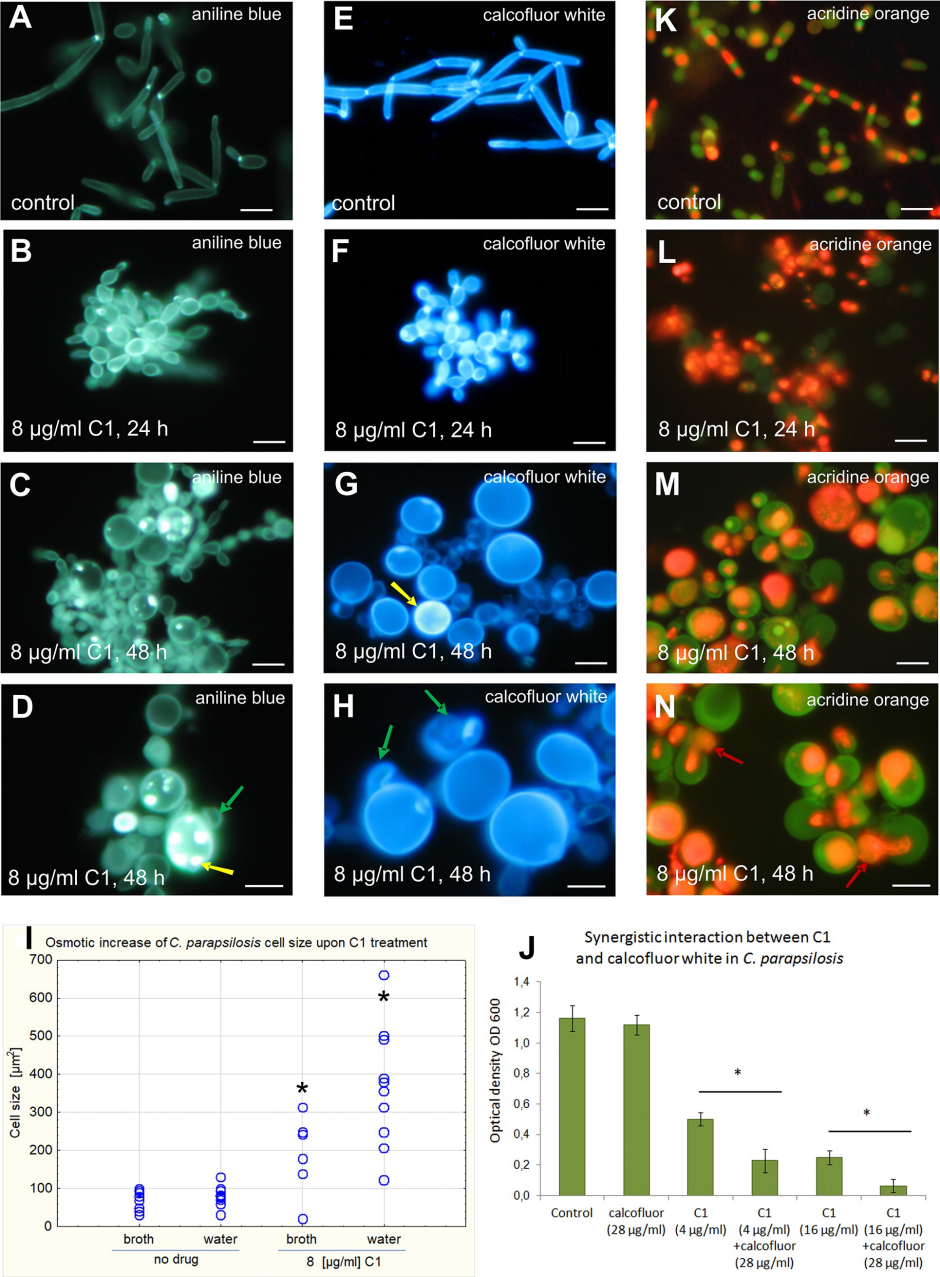
Abnormalities of cell walls and intracellular compartments of *C*. *parapsilosis* ATCC 22019 upon C1 treatment. **A-D.** Aniline Blue staining for β(1→3) glucan. **E-H.** Calcofluor White staining for chitin. **K-N**. Acridine Orange staining for acidic compartments. Red arrows–protoplasts being pushed out by internal turgor pressure; green arrows—defective cell walls; yellow arrows–defective β(1→3) glucan and chitin deposition. Scale bar = 10 μm. **I.** Distribution of the size (cell area in μm^2^) of cells pretreated with the C1 compound for 48 h then exposed to osmotic stress (water for 30 min) in comparison with drug non-treated cells (control cells). Measurements of 200 cells were made for each sample and are presented as scatter plots. Statistically significant differences between the averages of treated groups in relation to the untreated control are marked with asterisks on the plots and were determined by one-way ANOVA and a post-hoc Tukey test. Raw data are provided in Table K in S1 File. **J.** Growth of cells treated with Calcofluor White and C1 expressed as the final OD_600_ obtained after 48 h at 35°C. Assays were repeated three times with 4 replicates (wells). Results presented are therefore the average of 12 individual measurements. Raw data are provided in Table M in S1 File. *–statistically significant difference between the treatment groups (*p*<0.05) as determined by one-way ANOVA and a post-hoc Tukey test.

The morphotypic analysis of the C1-treated *Candida* cells stained with Calcofluor White for chitin and Aniline Blue for β(1→3) glucan revealed that the synthesis of these cell wall components was not inhibited, but impaired distribution of these substances was observed (Figs 5A–5I and 6A–6H). Irregular clusters and unusually intense fluorescence of chitin and β(1→3) glucan were visible in many cells, while the new budding cells were deprived of these substances. After Calcofluor White staining of the C1 treated cells, no fluorescence of chitin in the septum separating the new cell from the parental cell was observed in a majority of budding cells. To determine unambiguously the effect of C1 on the formation of the chitinous septum, further observations of this phenomenon under an transmission electron microscope would be necessary. The incorrect polymerization of cell wall components is evidenced by the fact that all C1-treated cells had a spherical shape. The images clearly indicate that the wall of many cells was ruptured and protoplasm was leaking through the cell wall (Figs 5D, 5J, 6G and 6H). It is likely that such protoplasts restore internal cell wall layers composed of chitin and β(1→3) glucan and exhibited intense fluorescence after staining with Aniline Blue and Calcofluor White. Additionally, next to many enlarged cells, there was an adhering empty cell wall (Figs 5C, 5E, 5I, 6D, 6G and 6H). Ruptured cell walls accompanied by protoplast material leaking out of the cell were also visible. Between the cells, there was an amorphous substance probably formed by degraded cells and cell wall residue (Fig 5B–5E, 6C and 6G). The *Candida* cells treated with the C1 compound were also checked for fluorescence without any additional fluorochrome staining to exclude background fluorescence that might confound the interpretation of the Calcofluor White and Aniline Blue data. The results showed no visible fluorescence under filters used for Calcofluor White, Aniline Blue, and Acridine Orange observations. An example of a picture of the lack of autofluorescence of *C*. *albicans* cells treated with C1 is shown in Fig 5R.

**Fig 7:**
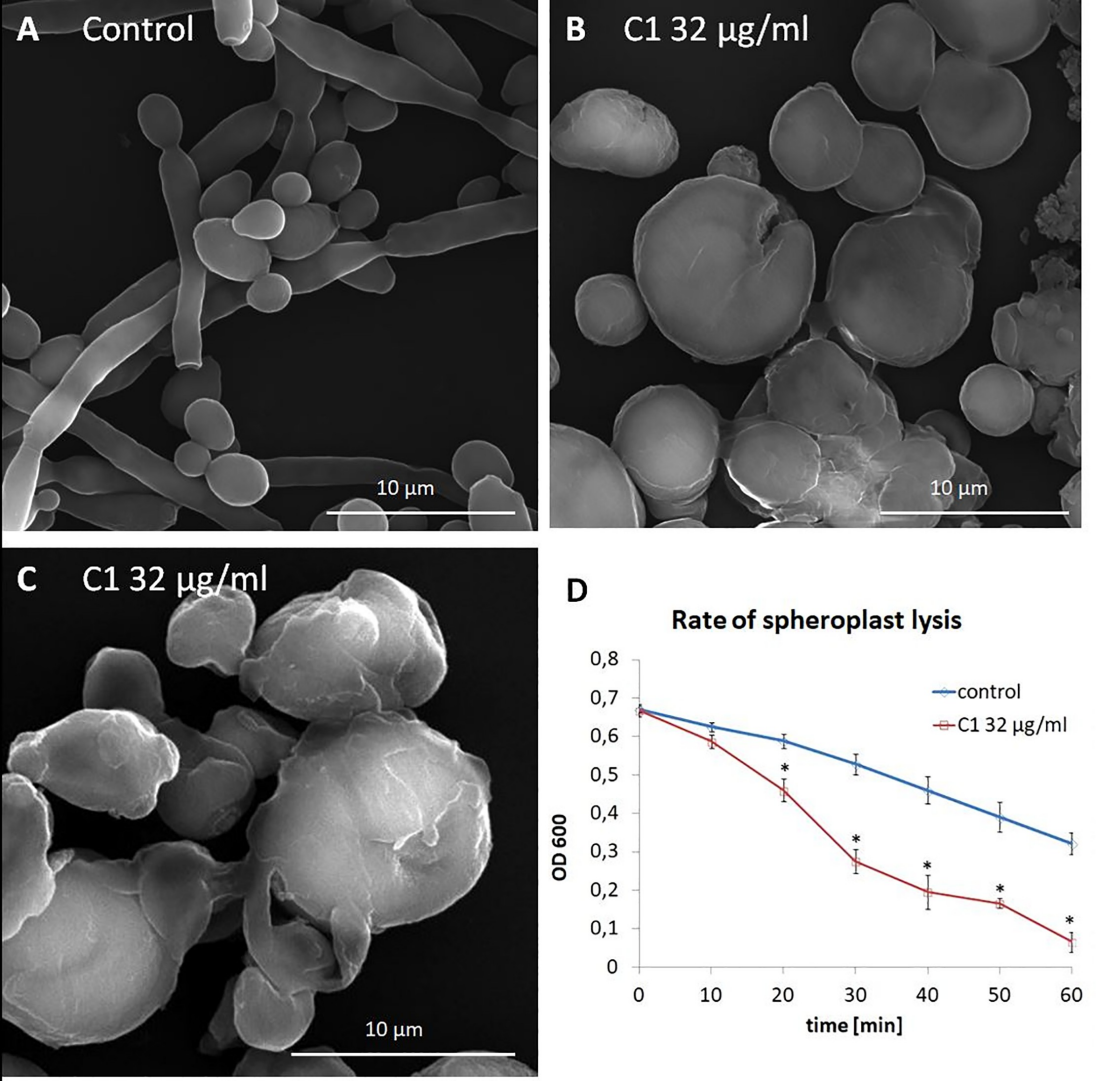
Changes in *C*. *albicans* NCPF 3153 cell wall integrity under C1 treatment. **A-C.** Scanning electron micrographs of *C*. *albicans* control (no drug added) (A) and C1-treated (32 μg/ml) cells (B, C). Images are representative of 3 independent preparations of each sample. Scale bars = 10 μm. **D.** The rate of *C*. *albicans* NCPF 3153 spheroplast lysis, as indicated by the decrease in OD_600_, under enzymatic digestion of the cell wall in untreated (control) and C1-treated cells (32 μg/ml). The experiment was repeated three times with two replicates (n = 6) and the average values with standard deviation are presented on the plot. *—statistically significant differences between the control and C1–treated cells as determined by multivariate analysis of variance (MANOVA), and a post-hoc Tukey (HSD) test. Raw data are provided in Table N in S1 File.

Multicellular aggregates (Fig 6B and 6F) can form as a result of impaired cytokinesis or as a result of flocculation. Studies carried out using SEM showed that the wall of untreated cells was smooth and regularly covered the yeast and hyphal morphotypes (Fig 7A). In the C1 treated cells (32 μg/ml for 24 h), abnormalities in the structure of the cell walls were observed, such as an irregular wall surface, and cracking and shedding of wall material (Fig 7B and 7C). The residue from degraded cell walls appeared to form an amorphous substance that stuck together numerous cells to form multicellular aggregates (Fig 7B and 7C).

The reduction of cell wall integrity was also demonstrated by the reduced osmotic resistance of cells treated with the C1 compound. The control cells of both tested *Candida* species cultured in RPMI 1640 medium for 48 h and then placed in water for 30 min showed no statistically significant increase in their size (Figs 5K and 6I), indicating that the osmotic influx of water into the cells was balanced by a properly polymerized wall. In contrast, cells grown in the presence of C1 at a concentration of ¼ MIC_70_ for 48 h and then transferred to pure water for 30 min were characterized by a rapid increase in their size caused by an osmotic influx of water into their interior, which was not prevented by walls with compromised integrity. The average size of *C*. *albicans* cells affected by the C1 treatment increased to 280.1 ± 246.5 μm^2^ after 30 min incubation in water, while cells left in the medium maintained an average size of 65.8 ± 56 μm^2^ (Fig 5K). In the case of *C*. *parapsilosis* pretreated with C1, the cell size increased to 350.2 ± 156.5 μm^2^ during 30 min of incubation in water, whereas the size of cells left in the culture medium remained at the level of 189.6 ± 99.6 μm^2^ (Fig 6I). Extremely large cells, reaching 500–700 μm^2^, as well as cells with extremely small sizes of approx. 3–5 μm^2^, which were probably empty walls or cell fragments, were visible in the hypoosmotic stress conditions. Some of the C1-pretreated cells kept in water swelled to enormous sizes, but did not burst immediately, which indicates that some of the cell wall layers were preserved, although with compromised integrity. On the other hand, the disrupted cell wall integrity caused swelling and ultimately cell lysis due to turgor pressure in many cells.

To confirm that the cell wall biogenesis was affected by the C1 compound, the interaction of C1 with Calcofluor White, which disrupts the polymerization of the fungal cell wall, was determined. An antifungal synergistic interaction was observed for these substances. Calcofluor White when applied individually at a concentration of 28 μg/ml did not significantly affect the growth of the cells of both *Candida* species, while the combined application with the C1 compound potentiated its antifungal activity by about 43% in *C*. *albicans* (Fig 5L) and by about 23% in *C*. *parapsilosis* (Fig 6J). Based on the results of this experiment, it can be concluded that fungal cells treated with the C1 compound exhibited cell wall defects, with enhanced sensitivity of such cells to compounds that interfere with polymerization of the cell wall components (Calcofluor White).

To determine whether the C1 compound affected cell wall integrity, an assay of the rate of spheroplast lysis during enzymatic digestion of the cell walls was performed. Untreated (control) cells and C1-treated (32μg/ml for 24 h) cells of *C*. *albicans* subjected to the action of the glucanase-protease mixture exhibited different rates of spheroplsts lysis in hypotonic conditions (Fig 7D). C1-treated cells were more sensitive to the action of the wall-digesting enzymes, as shown by the faster decrease in the OD_600_ in comparison to the untreated cells (Fig 7D). A statistically significant difference between the OD_600_ of the C1-treated and control cells was recorded after 20 min of enzymatic digestion and was maintained until the 60^th^ minute of the experiment. The results of this experiment confirmed the impaired integrity of *C*. *albicans* cells growing in the presence of the C1 compound.

### C1 causes disorders in intracellular compartments

Acridine Orange is a substance that fluoresces in red-orange color at low pH (2–3) and in green at pH ≥ 5. This was used to highlight intracellular compartments with low pH. In the control cells, orange-red fluorescence was observed only in the vacuoles. In *C*. *albicans*, each blastoconidium contained one regular vacuole which occupied about ¼ of the cell volume (Fig 5M), while the cylindrical cells of *C*. *parapsilosis* usually had two small regular vacuoles (Fig 6K). In contrast, severe disturbances in intracellular acidic compartments were observed in cells treated with the C1 compound at a concentration of ¼ MIC_70_. In many cells, a red-orange color spread throughout the whole cytoplasm. In the other groups of cells, there were multiple vacuoles with different sizes and irregular shapes, occupying a larger area of the cytoplasm than in the control cells (Figs 5N–5P and 6L–6N). Such vacuolar disorders may indicate the process of autophagy occurring in cells with impaired wall biogenesis. In many cells, this staining revealed breaks in cell walls and protoplasts being pushed out by internal turgor pressure (Figs 5P and 6N). In cultures treated with the C1 compound for 48 h, there was also a red-orange fluorescent amorphous extracellular substance composed of degraded cells and remnants of the cell walls (Fig 6M and 6N), which was absent in the control cultures (Fig 6K).

### The C1 compound does not affect the ergosterol level

**Fig 8:**
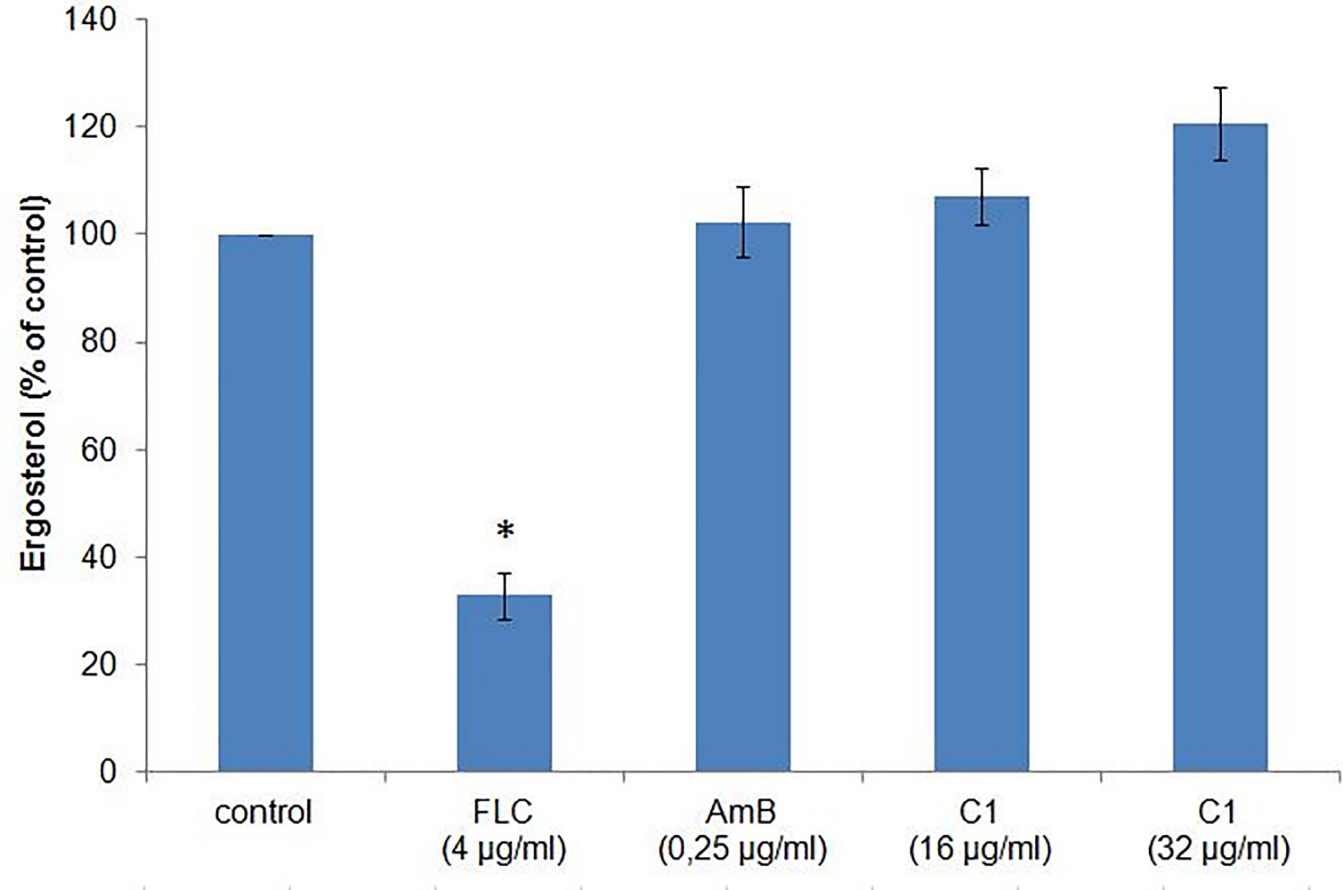
Ergosterol level expressed as the control percentage in *C*. *albicans* NCPF 3153 cells treated with fluconazole (FLC), amphotericin B (AmB), and the C1 compound. *C*. *albicans* cells were cultured in RPMI 1640 medium, without phenol red and sodium bicarbonate, buffered to pH 7.0 with 0.165 mol/l MOPS and enriched with 2% glucose, with addition of the C1 compound at the concentrations of ½ and ¼ MIC_70_, FLC at the concentration of ½ MIC_70_, or AmB at the concentration of ½ MIC_100_, for 24 h with shaking at 35°C. The ergosterol level was measured with the spectroscopic method. The average values from three repetitions are presented. The results are presented as the percentage of ergosterol content in the treatment samples relative to the control sample. The percentage of the control was calculated according to the following equation: (% of ergosterol in wet weight of the treated sample) / (% of ergosterol in wet weight of the untreated sample) x 100. Next, the average value of the percentage of the ergosterol content of the treated samples in relation to the control was calculated. Error bars represent the standard deviation. Raw data are presented in Table O in S1 File. *–statistically significant difference in comparison to the control (*p*<0.05) as determined by one-way ANOVA and post-hoc Tukey test.

Because the mechanism of the antifungal activity of drugs from the azole group involves inhibition of the ergosterol biosynthesis pathway, it was checked whether the C1 compound (a 1,3,4-thiadiazole) caused a decrease in the level of ergosterol in fungal cells. Treatment of *C*. *albicans* with the C1 compound, even at high concentrations (16 and 32 μg/ml), did not reduce the cell ergosterol content in comparison with the control cells (Fig 8). For additional verification of the effectiveness of the applied method for determination of the ergosterol level, an experiment was performed in which *C*. *albicans* cells were treated with FLC and AmB, whose mechanisms of antifungal activity are known. In the case of FLC, which inhibits the activity of the enzyme in the ergosterol biosynthesis pathway, a significant reduction in the ergosterol content was found compared with the control. In turn, no reduction in the ergosterol level was induced by AmB, although the compound causes loss of integrity of the fungal cell membrane (Fig 8). These results lead to a conclusion that the mechanism of the antifungal activity of the C1 compound does not rely on inhibition of ergosterol biosynthesis.

### ATR-FTIR analysis shows that the C1 compound causes changes in the cell wall of *C*. *albicans*

**Fig 9:**
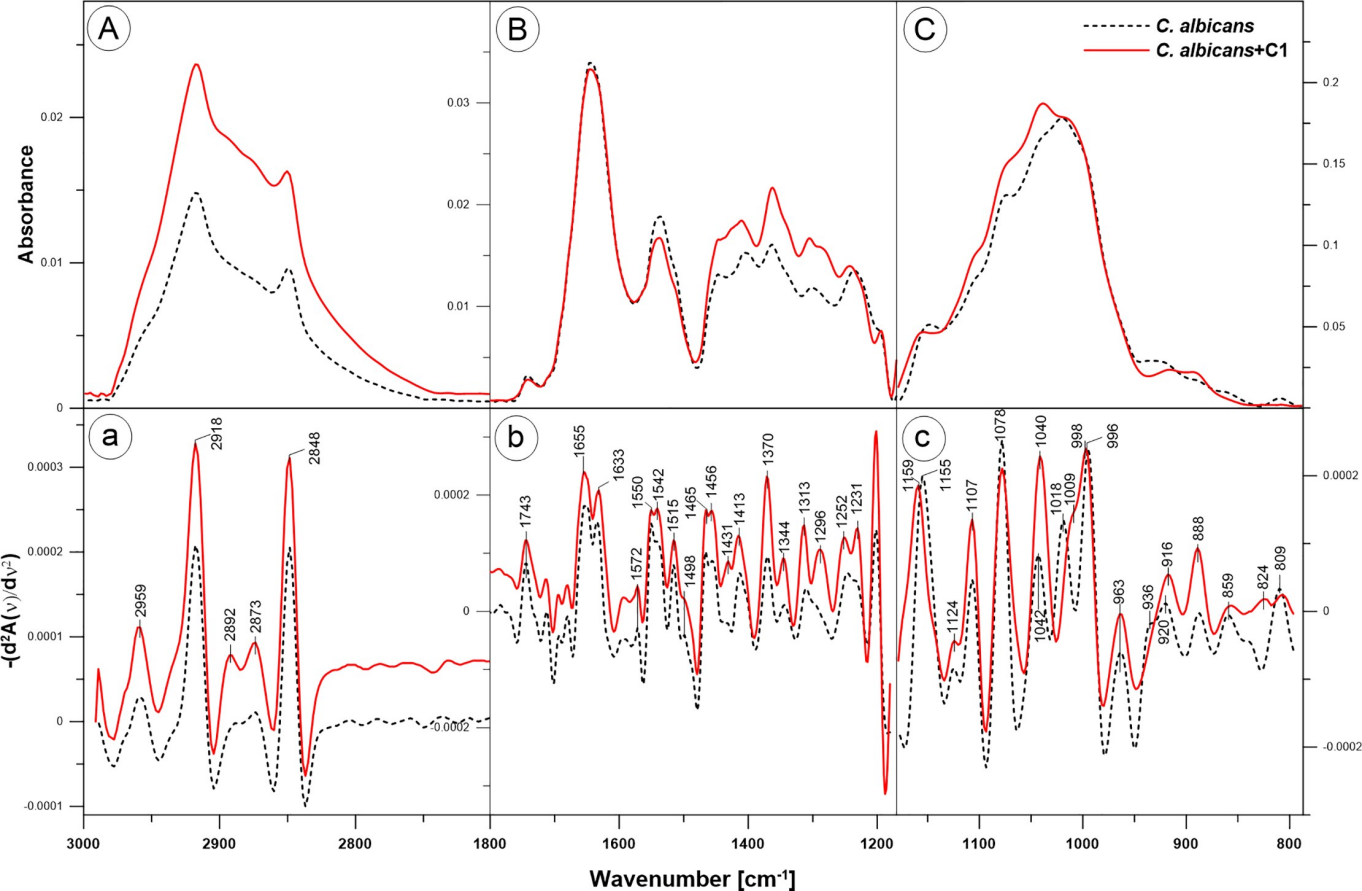
ATR-FTIR spectra of intact *C*. *albicans* control cells and cells treated for 24 h with the C1 compound at the concentration of 32 μg/ml. **A.** ATR-FTIR spectra in the region from 3000–2600 cm^-1^. **B.** Spectra in the region from 1800–1200 cm^-1^. **C.** Spectra in the region of 1200–800 cm^-1^. **a-c:** reverse second derivatives of spectra presented in A-C. The experiment was repeated three times and duplicate samples were included in each repeat. From each replicate sample three spectra were measured. A representative spectra and the reverse second derivatives of spectral values of the cell walls isolated from control cells and from cells treated with 32 μg/ml of C1 are presented.

The ATR-FTIR spectra of cell walls isolated from *C*. *albicans* cells, both the control and those treated with the C1 compound at the concentration of 32 μg/ml, are shown in Fig 9A–9C. Spectral differences can be better appraised by inspecting the reverse second derivative of the mean of the FTIR absorption spectra, as they help to resolve the problem of the overlapping components of the IR absorption bands. The reverse second derivatives of the spectra, corresponding to the regions defined above, are shown in Fig 9A–9C. The comparison of the reverse second derivatives of the spectra clearly indicates that the cell walls isolated from C1-treated *C*. *albicans* cells exhibit some important spectral modifications in comparison with the walls from the control cell. The spectral assignments were performed based on previously published data as indicated in Table 2.

**Table 2:** Wavenumbers (cm^-1^) and assignments of ATR-FTIR bands for *C*. *albicans* control and C1 (32 μg/ml)-treated cells; υ stretching, δ deformation, τ bending.

Position of bands [cm^-1^]		Type and origin of vibrations	References
walls of control cells	walls of C1-treated cells		
2959	2959	υ_asym_ CH_3,_ chitin	[25]
2918	2918 extended bandwidth profile	υ_asym_ CH_2_ β-glucan and chitin	[25, 26]
2892	2892	υ C-H β-glucan and chitin	[27, 28]
2873	2873	υ_sym_ CH_3_,chitin	[25]
2848	2848	υ_sym_ CH_2,_ β-glucan, chitin	[26]
1655	1655	Amide I: υ C = O, chitin, proteins	[25, 29]
1633	1633	Amide I, chitin, proteins	[25, 29]
1550	1550	Amide II: υ C-N, τ N-H chitin, proteins	[25, 28, 29]
1542	1542	Amide II chitin, proteins	[25, 29]
1313	1313 and 1296	Amide III: δ C-N chitin, proteins	[30, 31]
1252	1252	δ N-H chitin, proteins	[25]
1155	1159	β(1→3) glucan	[25, 32, 33]
1107	1107	β(1→3) glucan	[33, 34]
1042	1040	mannans	[32]
1018	-	β glucans	[25, 26]
996	998	β(1→6) glucan	[33, 34]
963	963	mannans	[33, 34]
936	-	glucans	[26]
888	888	glucans	[30, 34]

In the region of wavenumbers from 3000 to 2700 cm^−1^ (Fig 9A, a) bands characteristic for yeast wall polysaccharides were recorded. The bands centered at 2918 cm^−1^ and 2848 cm^-1^ can be ascribed to asymmetric and symmetric vibrations of CH_2_ groups, respectively, in β-glucan and chitin molecules [25, 26]. The bands at 2959 cm^−1^ and 2873 cm^−1^ reflect the asymmetric and symmetric vibrations of CH_3_ groups, respectively, in chitin molecules [25]. The band at 2892 cm^−1^ indicate the stretching vibrations of C-H bindings in polysaccharides [27, 28]. In the described region, no spectral shifts were observed in the walls isolated from C1-treated cells compared to the walls from the control cells. The only noticeable change was the widening of a profile of the band with a maximum at 2918 cm^−1^_._ However, a higher intensity of the bands related to β-glucan and chitin was observed in the C1-treated cells in comparison to the control cells. This result may indicate a higher content of β-glucan and chitin in *C*. *albicans* cells growing upon the C1 compound.

In the wavenumbers from 1800 to 1500 cm^−1^ (Fig 9B, b), the amide I and amide II bands are the two most prominent vibrational bands of the protein backbone and the most sensitive spectral region in the protein secondary structure. Similarly, the amide I and II bands are characteristic for chitin molecules [25]. The applied in the present work procedure of isolation of the yeast cell walls retains covalently bound proteins. Therefore, the observed amide bands reflect the vibrations of the bonds present in the chitin as well as in the wall proteins. When comparing the spectrum of cell walls isolated from control cells and treated with C1, No spectral shifts were observed between the amide I bands (1655 cm^−1^, 1633 cm^−1^), whereas in the amide II bands the change of the peak ratio 1550/1542 cm^−1^was observed. The band of amide III was found in both samples at 1313 cm^−1^, but in the C1-treated cells a new band at 1296 cm^−1^ appeared. Similarly, the band characteristic for δ N-H vibrations was recorded at 1252 cm^−1^ in both samples, but in the C1-treated cells an additional peak at 1231 cm^−1^ appeared. According to the literature, the spectral region of 1250–1230 cm^−1^ can also be related to the antisymmetric stretching of the double bond P = O in the phosphomannans of *N*-mannans. Phosphomannan is fairly abundant in many *Candida* strains [35, 36]. A detailed interpretation of these changes requires further research, perhaps starting with the removal of covalently bound proteins from the isolated cell wall material to interpret whether the observed changes concern the proteins or chitin.

The most pronounced changes in the C1-treated cells in comparison to the control cells were observed in the polysaccharide absorption region (1200 to 800 cm^-1^) of the FTIR spectra (Fig 9C, c). The spectral shifts towards higher frequencies were observed for the bands related to β(1→3) glucan: the band centered at 1155 cm^-1^ in the walls of control cells was shifted to 1159 cm^-1^ in the walls of C1-treated cells. Similarly, the band related to β(1→6) glucan, centered in the control cells at 996 cm^-1^ was shifted to 998 cm^-1^ in the cells upon C1 treatment. These spectral shifts may be related to weakened interactions between cell wall molecules to which they are assigned. In contrast, the band related to mannans centered in the control *C*. *albicans* cells at 1042 cm^-1^ was shifted by 2 cm^-1^ towards lower frequencies in the C1-treated cells, which may be related to stronger interactions between these molecules. A significant increase in the absorption intensity of bands related to mannans (1042/1040 cm^-1^ and 963 cm^-1^) in the C1-treated cells was also observed. Comparing the FTIR spectra of C1-treated cells with the spectra of control cells, the disappearance of a two glucan-related bands was also observed (1018 cm^-1^ and 936 cm^-1^). However, in the 925–800 cm^-1^ region which provides information about the mannan and total glucan content, increased absorbance of the samples isolated from C1-treated cells in comparison to the control cells was recorded.

### The C1 compound is not toxic to human cells

**Fig 10:**
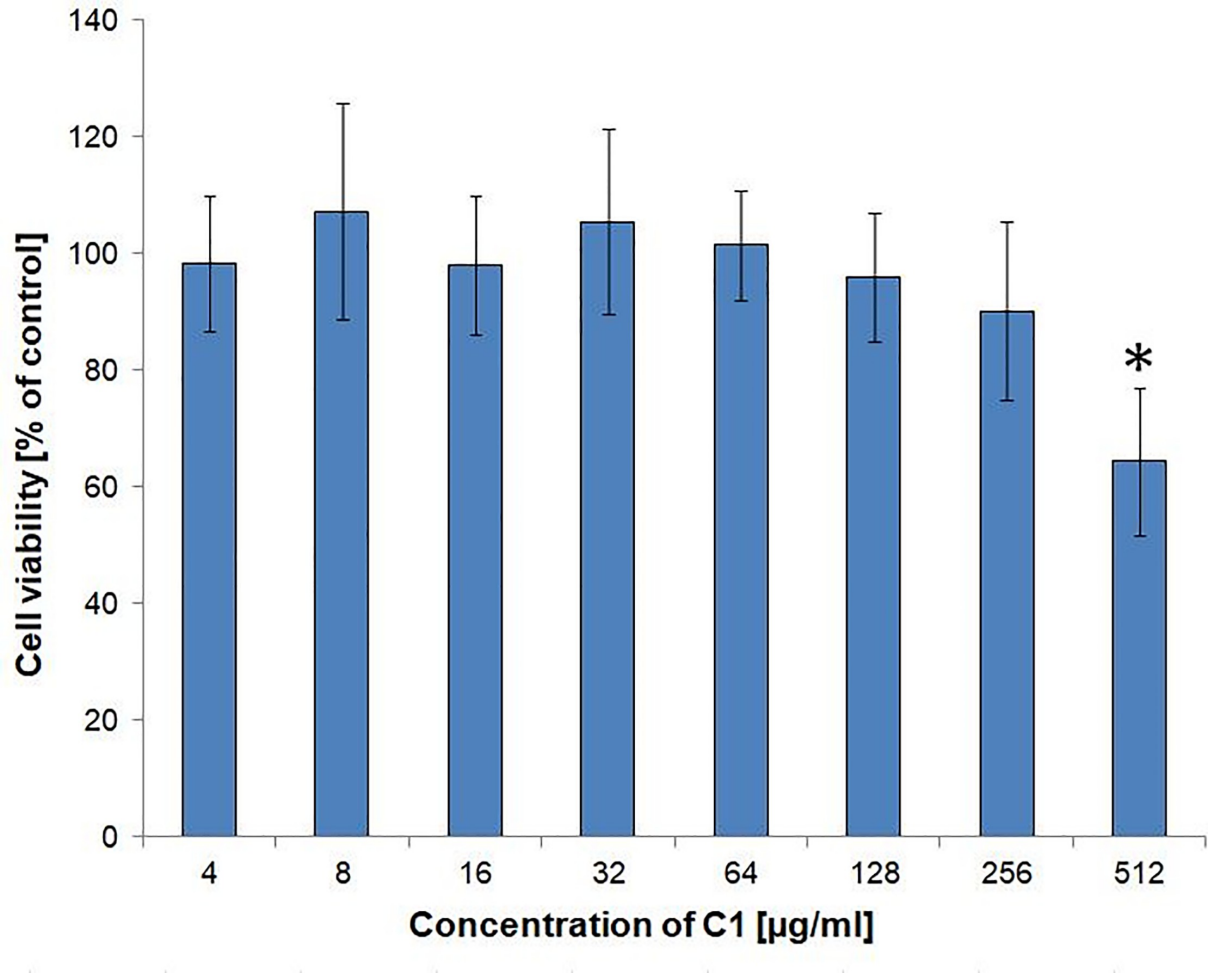
Viability of normal human dermal fibroblasts (NHDF) upon C1 treatment. The NHDF cells (Lonza, CC-2511, Bazel, Switzerland) were cultured in Dulbecco’s modified Eagle medium nutrient mixture F-12 HAM, supplemented with 10% fetal bovine serum in an incubator with humidified atmosphere saturated with 5% CO_2_ at 37ºC, for 96 h. The treatment groups were cultured in the presence of C1 compound at the concentrations of 4–512 μg/ml. cell viability was measured spectrophotometrically using the MTT assay and expressed as a percentage of the control. The assays were repeated three times with 8 replicates (wells) for each concentration. The results presented are therefore the average of 24 individual measurements. Error bars represent the standard deviation. Raw data are presented in Tables P and R in S1 File. Statistically significant differences between the treated samples and the control sample were marked with asterisks on the plot and were determined by one-way ANOVA and a post-hoc Tukey test.

The cytotoxicity of the C1 compound was assessed *in vitro* in normal human dermal fibroblasts (NHDF). The method of spectrophotometric determination of the amount of MTT reduction product after 72 h of culture revealed no significant decrease in the viability of the C1-treated cells, up to a concentration of 256 μg/ml, in comparison with the control cells (Fig 10). There were also no morphological disturbances in the C1-treated cells for these concentrations. Inhibition of cell viability to the level of 64.3 ± 12.6% of the control group was observed at the C1 concentration of 512 μg/ml. This C1 concentration can therefore be described as a dose inhibiting cell viability by 50% (IC_50_). The therapeutic index (IC_50_/MIC_100_) ranged from 5.3 to 64, depending on the fungal strain.

## Discussion

The morphological disturbances observed in *Candida* cells under the influence of C1 suggest that this compound acts by affecting the cell wall biogenesis. Numerous disorders related to abnormal polymerization of the cell wall were observed in the C1-treated fungal cells, including the inability to maintain the characteristic shape of particular morphotypes, an increase in the cell size and formation of giant cells, intense flocculation, reduced osmotic resistance, cell wall shedding and bursting, protoplast pushed out from the walls, formation of buds not covered with chitin, defective distribution of chitin and β(1→3) glucan, and increased sensitivity to substances interacting with wall polymerization (Calcofluor White). The spheroplast lysis assay, conducted under enzymatic digestion of the cell wall components in the hypotonic conditions, showed that the C1-treated cells are more sensitive to β-glucanase and protease activity in comparison to the control cells. This assay is recommended as a tool for studying the cell wall integrity in yeast [21]. Studies carried out using the scanning electron microscopy, showed the abnormalities in the structure of the cell walls upon C1 treatment. These disturbances consisted of irregular wall surface, cracking and shedding of wall material. Additionally, under the influence of the C1 compound, disturbances in intracellular compartments with low pH were observed. This phenomenon indicates functional disorders of the cells, perhaps a process of autophagy or necrotic cell death requiring additional research. Abnormal cell morphology, similar to that observed upon the C1 treatment, was reported as a common phenotype in cells treated with agents that are known to affect the cell wall [37, 38]. According to the literature, antifungals that affect the cell wall biogenesis, such as echinocandins, cause loss of cell wall integrity and ultimately lead to cell lysis due to turgor pressure [39]. Other authors have reported that *C*. *albicans* cells with impairment of cell wall integrity due to the mutation in the gene encoding the Ecm33 protein (a glycosylphosphatidylinositol-anchored cell wall protein) exhibit similar morphological changes to those observed upon the C1 treatment [40, 41]. The authors described *C*. *albicans* cells with affected cell wall integrity as giant, round and containing large vacuoles similar to autophagic cells. Additionally, those cells exhibited hypersensitivity to temperature, osmotic and oxidative stress, and a shortened chronological lifespan.

To confirm the influence of the C1 compound on the *C*. *albicans* cell wall composition, the analysis of ATR-FTIR spectra of isolated cell walls was performed.

The ATR-FTIR spectra of the C1 compound dissolved in ethanol and at different mol % concentrations in dipalmitoylphosphatidylcholine (DPPC) liposomes were previously presented in the publication by Kluczyk et al. [[42]]. The spectra presented in this paper show that even at high molar % concentrations of C1 in DPPC (up to 25 mol %), the characteristic bands for the vibrations of functional groups of this compound are not visible. The changes observed in the DPPC liposomes spectra containing C1 result from interactions of this compound with lipid layers. In the present work, the C1 compound was added to *C*. *albicans* cell culture at a concentration of 32 μg/ml, and after 24 h of incubation, the cells walls were isolated. The concentration of the C1 compound that was bound to the yeast cell walls was too low to be visualized in the form of vibrations of the characteristic functional groups of this compound in the FTIR spectra. We conclude, therefore, that the visible changes in the spectra between the control and the C1–treated *C*. *albicans* cells result from the active influence of this compound on the cells, and not merely from its presence in the cell wall samples. The most evident spectral shifts in the *C*. *albicans* C1-treated cells, in comparison with the control cells, were related to characteristic changes in the cell wall polysaccharides. The cell wall of the fungi from the *Candida* genus has a bilaminate structure composed of an outer layer of fibrillar mannoproteins and a branched core of β(1→3) glucan with interchain β(1→6) glucan linked to chitin via a β(1→4) linkage [43]. Branched β(1→3) glucan accounts for about 50–55%, interchain β(1→6) glucan represents 10–15%, whereas chitin as a polymer of β(1→4) linked *N*-acetylglucosamine accounts for only 1–2% of the total yeast cell wall polysaccharides [44]. β(1→6) glucan stabilizes the cell wall, since it plays a central role as a linker for specific cell wall components, including β-(1,3)-glucan, chitin, and mannoproteins [44]. Chitin forms a scaffold for cross-linking of β(1→3) glucans and β(1→6) glucans, which determine cell wall rigidity and stability [37, 45]. The core is subjected to continuous synthetic elaboration, degradation, and remodeling by a large arsenal of enzymes, whose activities must be appropriately balanced to provide the cell wall with adequate elasticity to allow growth, budding, or branching and yet sufficient strength to protect against cell lysis [43]. For proper polymerization of the cell wall and normal morphogenesis, the formation of the cross-linking is as important as the content of particular polysaccharides. In budding yeast, it was demonstrated that attachment of the chitin ring, which forms at the mother-bud neck during budding, to β(1→3) glucan in the cell wall is necessary for proper morphogenesis [46]. The visualized ATR-FTIR spectral shifts in the *C*. *albicans* cells treated with the C1 compound may indicate weakened interactions between the molecules of β(1→3) glucans and β(1→6) glucans, which can be a cause of the impaired cell wall integrity. Significant spectral changes in the C1-treated cells were also observed in bands characteristic for chitin. On the other hand, the obtained spectra suggest strongest interactions between mannan molecules and significantly increased absorption for mannans and glucans in the C1-treated cells, which may be a result of a defensive reaction of the yeast cells to weakened cross-linking between β(1→3) glucans and β(1→6) glucans. Other possible side of C1 interaction with the phosphate groups are phosphomannans, which are a part of *N*-mannans in many *Candida* species. Changes in the mannosyl-phosphate moiety are reported as a stress response during conditions of drought, high osmolarity, or nutrient limitation. Phosphomannans, as other cell wall components, are critical for a proper interaction of *C*. *albicans* with the components of the immune response [35, 36]. Proving such interactions obviously requires further detailed research. However, the analysis of the FTIR spectra of isolated cell walls does not allow the unambiguous conclusions about the specific molecules and interactions between them, because of the complexity of the sample. It can be only concluded that treatment of *C*. *albicans* cells with the C1 compound causes changes in the cell wall composition. Further studies are needed to elucidate the molecular mechanism of antifungal activity of the C1 compound. The supravital staining with fluorochromes showed that the C1 compound did not inhibit chitin or β(1→3) glucan synthesis in the *Candida* cells. In many cells, especially those with enlarged sizes, overproduction of these polysaccharides was observed, as evidenced by the intense fluorescence after the Calcofluor White and Aniline Blue staining. However, these substances were unevenly distributed and formed irregular clusters and were absent in the newly formed buds and in the septa, leading to impaired cytokinesis. The overproduction of chitin and β(1→3) glucans in the C1-treated cells may be a symptom of the defense mechanism of these cells in response to disrupted polymerization of wall components [47].

A proper structure and composition of the cell wall is necessary for pathogenic fungi to be virulent. For proper cell wall polymerization and morphogenesis, the cross-linking between wall elements, especially the chitin-glucan linkage, is critical, in addition to the content of individual polysaccharides and glycoproteins [46]. The participation of the cell wall in modulating host's immune response, avoidance of phagocytosis, and induction of immunotolerance are also subject to changes in response to environmental conditions and antifungal drugs [48, 49]. For effective tissue invasion, expression of proteins from the groups of invasins and adhesins on the surface of a properly polymerized cell wall is necessary. They are responsible for penetration into the host cells and adhesion to various biotic and abiotic surfaces [48]. It was shown that cells with impaired cell wall integrity were phagocytosed by macrophages to a wider extent than the wild-type strain, but the damage caused to mouse cells was smaller than in the case of the wild-type strain [40]. It was also reported that cells with impaired cell wall integrity had aberrant surface localization of adhesins and reduced adherence and capacity to invade and damage hosts' oral epithelial and endothelial cells [50]. Another important element of the virulence of *Candida* is the phenotypic flexibility in response to changing environmental conditions. *C*. *albicans* belongs to highly polymorphic fungi, creating *in vivo* and *in vitro* alternative vegetative forms in response to changing conditions, e.g. availability of nutrients, pH, temperature, and presence of serum. This phenotypic flexibility is an important virulence factor necessary for epithelial invasion, dissemination in tissues and various niches of the hosts' organism, as well as modulation of immune response and avoidance of attack by the immune system [48, 51]. The formation of hyphae is also an indispensable element in the formation of drug-resistant biofilms on various biotic and abiotic surfaces, which are a serious surgical problem in the implantation of valves, various types of catheters, and endoprothesis. The C1 compound was revealed to be highly active in inhibiting the hyphal formation in *Candida*. In the case of *C*. *albicans*, significant reduction of the formation of hyphae and pseudohyphae was induced by the C1 compound at the doses of 0.5 and 1 μg/ml, and complete inhibition was noted at the concentration of 2 μg/ml.

Summing up, in doses much lower than the determined MIC values, the C1 compound causes inhibition of the emergence of invasive forms as well as changes in the morphology and intracellular compartment distribution in *Candida* cells by interference in the cell wall biogenesis. Due to these effects on cell wall polymerization, cells with altered morphology are hypersensitive to osmotic, thermal, and oxidative stress and exhibit impaired adhesion to host tissues [40, 50]. The *in vitro* morphological disturbances and suppression of invasive forms in fungal pathogens induced by the C1 compound will probably occur *in vivo* as well. This may result in the loss of adhesion and invasiveness of the pathogen in the host tissues and increased susceptibility to phagocytosis. It may therefore be beneficial to administer even low doses of the C1 compound for therapeutic purposes. Since cells treated with C1 become hypersensitive to stress, this compound is most likely to show additive and synergistic interactions with other antifungal drugs. These observations should be taken into account when considering the possible use of the C1 compound for therapeutic purposes. It is worth noting that the C1 compound exhibits activity against fungal pathogens belonging to various species and genera, including *Candida* nonalbicans species, which are often characterized by resistance to echinocandins and azoles. It is particularly important that the *C*. *glabrata* and *C*. *parapsilosis* strains, which are less sensitive to echinocandins and often exhibit resistance [8], were sensitive to the C1 compound. Similarly, this compound has been shown to be active against clinical isolates of azole-resistant pathogenic fungi, which is related to the fact that C1 affects a different metabolic target than the ergosterol biosynthesis process. The C1 compound is also active against mold fungi, including *Aspergillus niger*, which is difficult to treat due to frequent intrinsic resistance to AmB.

Despite the relatively high MIC values for the C1 compound, its potential therapeutic use is worth considering, given its low cytotoxicity towards human cells and the fact that much lower doses than MICs are potent in inhibition of formation of hyphae and induction of morphological changes in fungal cells. The cytotoxicity of the C1 compound was tested on normal human dermal fibroblasts (NHDF cell line), because its application as a potential drug in treatment of surface mycoses is taken into consideration. The calculated therapeutic index (IC_50_/MIC_100_), depending on the fungal strain, ranged from 5.3 to 64, which makes the C1 compound applicable as a therapeutic agent in the treatment of surface and gastrointestinal mycoses. The possibility of using the C1 compound for treatment of systemic fungal infections will depend on the results of studies on its bioavailability after oral and injectable administration. Model biophysical studies of dipalmitoylphosphatidylcholine bilayers containing C1 molecules have shown that this compound does not cross the lipid bilayer, but interacts with the hydrophilic membrane surface [42]. This property probably results in the low cytotoxicity of the C1 compound to human cells. In the case of fungal cells, the C1 compound probably affects enzymes involved in cell wall biogenesis, which are located on the cell surface; therefore, it is not necessary for the compound to penetrate into the cytoplasm actively.

## Supporting information

S1 File Sensitivity of the pathogenic fungi studied in the present work to antifungals and raw data obtained in the experiments. (PDF)
