# Supplementary material for: Antifungal effects of a 1,3,4-thiadiazole derivative determined by cytochemical and vibrational spectroscopic studies
Source: PLoS One. 2019 Sep 30;14(9):e0222775. doi: 10.1371/journal.pone.0222775 (PMC6768478; doi:10.1371/journal.pone.0222775)
Supplement: S1 File — (PDF) [file pone.0222775.s001.pdf]

Table A. Susceptibility of pathogenic fungal isolates and standard strains to the antifungals studied with the ATB Fungus 3 strips reading method. Interpretation of the antibiogram according to EUCAST 8.8 (2018) recommendation; S – sensitive, R – resistant, MS – medium sensitive

Strain	antifungals				
	Amphotericin B	fluconazole	itraconazole	voriconazole	flucytosine
Candida albicans isolate 102	S	S	R	S	S
Candida krusei isolate 93	S	R	R	MS	MS
Candida dubliniensis isolate 176	S	S	R	S	R
Candida glabrata isolate 124	S	MS	MS	S	S
Candida krusei isolate 103	S	R	R	MS	S
Candida tropicalis isolate 175	S	R	R	R	S
Trichophyton rubrum ATCC 28188	S	S	S	S	S
Rhodotorula mucilaginosa ATCC 22273	S	R	S	S	S
Aspergillus niger ATCC 16888	MS	R	S	S	S
Candida albicans NCPF 3153	S (MIC_{100} 0.5 µg/ml)	S (MIC_{70} 8 µg/ml)	S	S	S
Candida parapsilosis ATCC 22019	MS (MIC_{100} 2 µg/ml)	S (MIC_{70} 16 µg/ml)	S	S	S

Table B. Inhibition of *C. albicans* NCPF 3153 growth by the C1 compound, measured as the OD_{600}. Results from this table were used for preparing Fig 2 in the main body of the paper.

	C. albicans 24 h									
**C1 [µg/ml]**	**Control**	**96.0**	**64.0**	**32.0**	**16.0**	**8.0**	**4.0**	**2.0**	**1.0**	**0.5**
Optical density (OD_{600})	1.160	0.000	0.000	0.434	0.530	0.780	1.120	1.150	1.150	1.116
	1.167	0.000	0.000	0.443	0.640	0.830	1.130	1.137	1.127	1.116
	1.268	0.000	0.000	0.315	0.620	0.810	1.210	1.318	1.220	1.215
	1.323	0.000	0.000	0.417	0.490	0.830	1.330	1.313	1.313	1.153
	1.240	0.000	0.000	0.527	0.672	0.786	1.140	1.144	1.224	1.260
	1.340	0.000	0.000	0.321	0.592	0.843	1.310	1.250	1.314	1.213
	1.220	0.000	0.000	0.345	0.567	0.872	1.320	1.230	1.241	1.232
	1.110	0.000	0.000	0.234	0.672	0.763	1.210	1.110	1.121	1.121
	1.210	0.000	0.000	0.392	0.672	0.832	1.130	1.250	1.161	1.231
	1.320	0.000	0.000	0.413	0.593	0.901	1.310	1.370	1.342	1.334
	1.210	0.000	0.000	0.467	0.743	0.862	1.240	1.310	1.231	1.221
	1.320	0.000	0.000	0.261	0.734	0.782	1.220	1.220	1.312	1.321
	C. albicans 24 h									
	**Control**	**96.0**	**64.0**	**32.0**	**16.0**	**8.0**	**4.0**	**2.0**	**1.0**	**0.5**
Control %	1.160	0.000	0.000	37.414	45.690	67.241	96.552	99.138	99.138	96.207
	1.167	0.000	0.000	37.961	54.841	71.123	96.829	97.429	96.572	95.630
	1.268	0.000	0.000	24.842	48.896	63.880	95.426	103.943	96.215	95.820
	1.323	0.000	0.000	31.519	37.037	62.736	100.529	99.244	99.244	87.150
	1.240	0.000	0.000	42.500	54.194	63.387	91.935	92.258	98.710	101.613
	1.340	0.000	0.000	23.955	44.179	62.910	97.761	93.284	98.060	90.522
	1.220	0.000	0.000	28.279	46.475	71.475	108.197	100.820	101.721	100.984
	1.110	0.000	0.000	21.081	60.541	68.739	109.009	100.000	100.991	100.991
	1.210	0.000	0.000	32.397	55.537	68.769	93.388	103.306	95.950	101.736
	1.320	0.000	0.000	31.288	44.924	68.258	99.242	103.788	101.667	101.061
	1.210	0.000	0.000	38.595	61.405	71.240	102.479	108.264	101.736	100.909
	1.320	0.000	0.000	19.773	55.606	59.242	92.424	92.424	99.394	100.076
mean		0.000	0.000	30.800	50.777	66.583	98.648	99.492	99.116	97.725
SD		0.000	0.000	7.390	7.367	4.029	5.620	5.019	2.131	4.799

	C. albicans 48 h									
	**Control**	**96.0**	**64.0**	**32.0**	**16.0**	**8.0**	**4.0**	**2.0**	**1.0**	**0.5**
Optical density (OD_{600})	1.621	0.000	0.482	0.932	1.643	1.543	1.674	1.543	1.654	1.621
	1.543	0.000	0.423	0.962	1.543	1.534	1.498	1.534	1.532	1.532
	1.678	0.000	0.293	0.897	1.621	1.642	1.632	1.648	1.543	1.612
	1.653	0.000	0.472	0.934	1.613	1.613	1.621	1.541	1.664	1.576
	1.546	0.000	0.521	0.942	1.532	1.513	1.553	1.524	1.646	1.621
	1.672	0.000	0.242	0.893	1.653	1.653	1.532	1.612	1.572	1.632
	1.593	0.000	0.372	0.976	1.521	1.543	1.498	1.524	1.521	1.623
	1.612	0.000	0.421	0.897	1.632	1.621	1.674	1.612	1.664	1.634
	1.643	0.000	0.513	0.986	1.613	1.663	1.676	1.612	1.687	1.623
	1.635	0.000	0.361	0.820	1.613	1.543	1.586	1.578	1.543	1.579
	1.564	0.000	0.332	0.821	1.432	1.521	1.521	1.601	1.521	1.612
	1.543	0.000	0.362	0.963	1.513	1.498	1.565	1.606	1.675	1.532
	C. albicans 48 h									
	**Control**	**96.0**	**64.0**	**32.0**	**16.0**	**8.0**	**4.0**	**2.0**	**1.0**	**0.5**
Control %	1.621	0.000	29.735	57.495	101.357	95.188	103.270	95.188	102.036	100.000
	1.543	0.000	27.414	62.346	100.000	99.417	97.084	99.417	99.287	99.287
	1.678	0.000	17.461	53.456	96.603	97.855	97.259	98.212	91.955	96.067
	1.653	0.000	28.554	56.503	97.580	97.580	98.064	93.224	100.665	95.342
	1.546	0.000	33.700	60.931	99.094	97.865	100.453	98.577	106.468	104.851
	1.672	0.000	14.474	53.409	98.864	98.864	91.627	96.411	94.019	97.608
	1.593	0.000	23.352	61.268	95.480	96.861	94.036	95.669	95.480	101.883
	1.612	0.000	26.117	55.645	101.241	100.558	103.846	100.000	103.226	101.365
	1.643	0.000	31.223	60.012	98.174	101.217	102.009	98.113	102.678	98.783
	1.635	0.000	22.080	50.153	98.654	94.373	97.003	96.514	94.373	96.575
	1.564	0.000	21.228	52.494	91.560	97.251	97.251	102.366	97.251	103.069
	1.543	0.000	23.461	62.411	98.056	97.084	101.426	104.083	108.555	99.287
mean		0.000	24.900	57.177	98.055	97.843	98.611	98.148	99.666	99.510
SD		0.000	5.656	4.213	2.664	1.985	3.700	3.061	5.192	2.901

Table C. Inhibition of *C. parapsilosis* ATCC 22019 growth by the C1 compound, measured as the OD_{600}. Results from this table were used for preparing Fig 2 in the main body of the paper.

	C. parapsilosis 24 h									
**C1 [µg/ml]**	**control**	**96.0**	**64.0**	**32.0**	**16.0**	**8.0**	**4.0**	**2.0**	**1.0**	**0.5**
Optical density (OD 600)	1.022	0.000	0.000	0.000	0.232	0.334	0.579	0.620	1.032	1.070
	1.040	0.000	0.000	0.000	0.245	0.413	0.568	0.740	1.021	1.040
	1.032	0.000	0.000	0.000	0.282	0.318	0.615	0.570	1.050	1.050
	1.021	0.000	0.000	0.000	0.242	0.421	0.489	0.770	1.040	1.010
	1.250	0.000	0.000	0.000	0.287	0.441	0.543	0.602	1.030	1.020
	1.288	0.000	0.000	0.000	0.182	0.377	0.531	0.702	1.030	1.040
	1.250	0.000	0.000	0.000	0.301	0.384	0.466	0.604	1.050	1.030
	1.200	0.000	0.000	0.000	0.273	0.348	0.538	0.601	1.070	1.050
	1.120	0.000	0.000	0.000	0.232	0.375	0.523	0.730	1.100	1.110
	1.150	0.000	0.000	0.000	0.198	0.228	0.561	0.590	1.260	1.050
	1.080	0.000	0.000	0.000	0.197	0.244	0.611	0.720	1.130	1.090
	1.140	0.000	0.000	0.000	0.352	0.211	0.456	0.550	1.100	1.150
	C. parapsilosis 24 h									
Control %	**Control**	**96.0**	**64.0**	**32.0**	**16.0**	**8.0**	**4.0**	**2.0**	**1.0**	**0.5**
	1.022	0.000	0.000	0.000	22.701	32.681	56.654	60.665	100.978	104.697
	1.040	0.000	0.000	0.000	23.558	39.712	54.615	71.154	98.173	100.000
	1.032	0.000	0.000	0.000	27.326	30.814	59.593	55.233	101.744	101.744
	1.021	0.000	0.000	0.000	23.702	41.234	47.894	75.416	101.861	98.923
	1.250	0.000	0.000	0.000	22.960	35.280	43.440	48.160	82.400	81.600
	1.288	0.000	0.000	0.000	14.130	29.270	41.227	54.503	79.969	80.745
	1.250	0.000	0.000	0.000	24.080	30.720	37.280	48.320	84.000	82.400
	1.200	0.000	0.000	0.000	22.750	29.000	44.833	50.083	89.167	87.500
	1.120	0.000	0.000	0.000	20.714	33.482	46.696	65.179	98.214	99.107
	1.150	0.000	0.000	0.000	17.217	19.826	48.783	51.304	109.565	91.304
	1.080	0.000	0.000	0.000	18.241	22.593	56.574	66.667	104.630	100.926
	1.140	0.000	0.000	0.000	30.877	18.509	40.000	48.246	96.491	100.877
mean		0.000	0.000	0.000	22.355	30.260	48.132	57.911	95.599	94.152
SD		0.000	0.000	0.000	4.450	7.135	7.288	9.644	9.493	8.889

	C. parapsilosis 48 h									
**C1 [µg/ml]**	**control**	**96.0**	**64.0**	**32.0**	**16.0**	**8.0**	**4.0**	**2.0**	**1.0**	**0.5**
Optical density (OD $_{600}$)	1.260	0.000	0.000	0.242	0.232	0.580	0.783	0.994	1.230	1.310
	1.250	0.000	0.000	0.342	0.323	0.427	0.749	0.990	1.310	1.200
	1.232	0.000	0.000	0.342	0.321	0.431	0.748	0.940	1.150	1.240
	1.241	0.000	0.000	0.253	0.514	0.413	0.651	0.990	1.250	1.310
	1.340	0.000	0.000	0.342	0.524	0.454	0.655	0.980	1.240	1.210
	1.320	0.000	0.000	0.202	0.320	0.420	0.745	0.940	1.320	1.190
	1.320	0.000	0.000	0.121	0.410	0.670	0.648	0.940	1.260	1.250
	1.340	0.000	0.000	0.142	0.360	0.676	0.462	0.690	1.190	1.340
	1.220	0.000	0.000	0.193	0.425	0.653	0.642	0.920	1.170	1.350
	1.260	0.000	0.000	0.145	0.423	0.643	0.754	0.810	1.240	1.420
	1.160	0.000	0.000	0.192	0.454	0.565	0.641	0.970	1.240	1.370
	1.190	0.000	0.000	0.125	0.425	0.531	0.736	0.920	1.260	1.210
	C. parapsilosis 48 h									
Control %	**Control**	**96.0**	**64.0**	**32.0**	**16.0**	**8.0**	**4.0**	**2.0**	**1.0**	**0.5**
	1.260	0.000	0.000	19.206	18.413	46.032	62.143	78.889	97.619	103.968
	1.250	0.000	0.000	27.360	25.840	34.160	59.920	79.200	104.800	96.000
	1.232	0.000	0.000	27.760	26.063	34.984	60.714	76.299	93.344	100.649
	1.241	0.000	0.000	20.387	41.418	33.280	52.458	79.774	100.725	105.560
	1.340	0.000	0.000	25.522	39.104	33.881	48.881	73.134	92.537	90.299
	1.320	0.000	0.000	15.303	24.242	31.818	56.439	71.212	100.000	90.152
	1.320	0.000	0.000	9.167	31.061	50.758	49.091	71.212	95.455	94.697
	1.340	0.000	0.000	10.597	26.866	50.448	34.478	51.493	88.806	100.000
	1.220	0.000	0.000	15.820	34.836	53.525	52.623	75.410	95.902	110.656
	1.260	0.000	0.000	11.508	33.571	51.032	59.841	64.286	98.413	112.698
	1.160	0.000	0.000	16.552	39.138	48.707	55.259	83.621	106.897	118.103
	1.190	0.000	0.000	10.504	35.714	44.622	61.849	77.311	105.882	101.681
mean		0.000	0.000	17.474	31.356	42.770	54.475	73.487	98.365	102.039
SD		0.000	0.000	6.664	7.101	8.420	7.878	8.577	5.597	8.727

Table D. Number of morphotypes of *C. albicans* NCPF 3153 in control culture and upon C1 treatment. Results from this table were used for preparing Fig 3A in the main body of the paper.

Type of morphotype	***Candida albicans*** **morphotypes**															
	**Control**		**0.5 µg/ml C1**		**1 µg/ml C1**		**2 µg/ml C1**		**4 µg/ml C1**		**8 µg/ml C1**		**16 µg/ml C1**		**32 µg/ml C1**	
	number	%	number	%	number	%	number	%	number	%	number	%	number	%	number	%
Hyphae	79	24.3	8	2.7	2	1.1	0	0.0	0	0.0	0	0.0	0	0.0	0	0.0
Single cells	94	28.9	47	15.7	28	15.0	15	8.6	5	3.9	4	3.6	5	6.1	7	7.2
Doublets	110	33.8	56	18.7	5	2.7	19	10.9	2	1.6	2	1.8	3	3.7	2	2.1
Triplets	32	9.8	48	16.0	13	7.0	6	3.4	4	3.1	5	4.5	1	1.2	5	5.2
Schort chains	10	3.1	56	18.7	23	12.3	21	12.1	12	9.4	4	3.6	4	4.9	3	3.1
Small aggregates	0	0.0	67	22.3	74	39.6	64	36.8	48	37.8	32	28.6	23	28.0	23	23.7
Big aggregates	0	0.0	18	6.0	42	22.5	49	28.2	56	44.1	65	58.0	46	56.1	57	58.8
Total	79		300		187		174		127		112		82		97	

Table E. Number of morphotypes of *C. parapsilosis* ATCC 22019 in control culture and upon C1 treatment. Results from this table were used for preparing Fig 3B in the main body of the paper.

Type of morphotype	***Candida parapsilosis* morphotypes**													
	**Control**		**0.5 µg/ml C1**		**1 µg/ml C1**		**2 µg/ml C1**		**4 µg/ml C1**		**8 µg/ml C1**		**16 µg/ml C1**	
	number	%	number	%	number	%	number	%	number	%	number	%	number	%
Cylindrical cells	203	67.7	110	36.7	26	8.7	8	2.7	0	0.0	0	0.0	0	0.0
Elipsoidal cells	97	32.3	176	58.7	189	63.0	78	26.0	36	12.0	8	2.7	0	0.0
Aggregates of globose cells	0	0.0	14	4.7	61	20.3	117	39.0	175	58.3	172	57.3	145	48.3
Multiaggregates of globose cells	0	0.0	0	0.0	24	8.0	97	32.3	89	29.7	120	40.0	155	51.7
Total	300		300		300		300		300		300		300	

Table F. Size of control and C1-treated *C. albicans* NCPF 3153 cells after 24h, 48h and 72 h of culture. Results from this table were used for preparing Fig 4A in the main body of the paper.

Candida albicans																	
**Control 24 h**			**Control 48h**			**Control 72h**			**C1 16 µg/ml, 24h**			**C1 16 µg/ml, 48h**			**C1 16 µg/ml, 72h**		
**Diameter A [µm]**	**Diameter B [µm]**	**Cell size [µm^2]**	**Diameter A [µm]**	**Diameter B [µm]**	**Cell size [µm^2]**	**Diameter A [µm]**	**Diameter B [µm]**	**Cell size [µm^2]**	**Diameter A [µm]**	**Diameter B [µm]**	**Cell size [µm^2]**	**Diameter A [µm]**	**Diameter B [µm]**	**Cell size [µm^2]**	**Diameter A [µm]**	**Diameter B [µm]**	**Cell size [µm^2]**
3	3.5	8.2	4	4	12.6	4	4	12.6	9	9	63.6	10	10	78.5	17.5	17.5	240.4
4.5	4.5	15.9	4	4	12.6	4	4	12.6	9.5	9.5	70.8	10	10	78.5	17.5	17.5	240.4
4	5	15.7	4	4	12.6	4	4	12.6	9	8.5	60.1	10	10	78.5	17.5	17.5	240.4
4.5	3	10.6	4	4	12.6	4	4	12.6	9	8.5	60.1	10	10	78.5	17.5	17.5	240.4
4.5	5	17.7	4	4	12.6	4	4	12.6	9	8.5	60.1	10	10	78.5	17.5	17.5	240.4
4.5	4.5	15.9	4	4	12.6	4	4	12.6	9	8.5	60.1	10	10	78.5	17.5	17.5	240.4
5	5	19.6	4	4	12.6	4	4	12.6	9	8.5	60.1	10	10	78.5	17.5	17.5	240.4
4.5	5	17.7	4	4	12.6	4	4	12.6	9	8.5	60.1	10	10	78.5	17.5	17.5	240.4
4	4	12.6	4	4	12.6	4	4	12.6	9	8.5	60.1	10	10	78.5	17.5	17.5	240.4
5	4.5	17.7	4	4	12.6	4	4	12.6	9	8.5	60.1	10	10	78.5	17.5	17.5	240.4
4.5	4.5	15.9	4	4	12.6	4	4	12.6	9	8.5	60.1	10	10	78.5	17.5	17.5	240.4
3.5	3.5	9.6	4	4	12.6	4	4	12.6	9	8.5	60.1	10	10	78.5	17.5	17.5	240.4
5.5	4.5	19.4	4	4	12.6	4	4	12.6	9	8.5	60.1	10	10	78.5	17.5	17.5	240.4
4.5	4.5	15.9	4	4	12.6	4	4	12.6	9	8.5	60.1	10	10	78.5	17.5	17.5	240.4
4	4	12.6	4	4	12.6	4	4	12.6	9	9	63.6	10	10	78.5	17.5	17.5	240.4
3	4	9.4	4	4	12.6	4	4	12.6	10	10.5	82.4	10	10	78.5	17.5	17.5	240.4
3	5	11.8	4	4	12.6	4	4	12.6	10	10	78.5	10	10	78.5	17.5	17.5	240.4
5.5	7.5	32.4	4	4	12.6	4	4	12.6	10	10	78.5	10	10	78.5	17.5	17.5	240.4
5	6.5	25.5	4	4	12.6	4	4	12.6	10	10	78.5	10	10	78.5	17.5	17.5	240.4
5	6.5	25.5	4	4	12.6	4	4	12.6	10	10	78.5	12	12	113.0	17.5	17.5	240.4
5	6	23.6	3.5	4	11.0	4	4	12.6	10	10	78.5	12	12	113.0	22.5	19	335.6
4.5	6	21.2	3.5	4	11.0	4	4	12.6	10	10	78.5	12	12	113.0	22.5	19	335.6
4.5	7.5	26.5	3.5	4	11.0	4	4	12.6	10	10	78.5	12	12	113.0	22.5	19	335.6
5.5	5.5	23.7	3.5	4	11.0	4	4	12.6	10	10	78.5	12	12	113.0	22.5	19	335.6
5	7	27.5	3.5	4	11.0	4	4	12.6	10	10	78.5	12	12	113.0	22.5	19	335.6
4.5	5	17.7	3.5	4	11.0	4	4	12.6	10	10	78.5	12	12	113.0	22.5	19	335.6

5.5	5	21.6	3.5	4	11.0	4	4	12.6	7	7	38.5	12	12	113.0	22.5	19	335.6
4.5	6	21.2	3.5	4	11.0	4	4	12.6	7	7	38.5	12	12	113.0	22.5	19	335.6
4.5	6.5	23.0	3.5	4	11.0	4	4	12.6	7	7	38.5	12	12	113.0	22.5	19	335.6
4.5	7	24.7	3.5	4	11.0	4	4	12.6	7	7	38.5	12	12	113.0	22.5	19	335.6
4.5	7	24.7	3.5	4	11.0	4	4	12.6	7	7	38.5	12	12	113.0	22.5	19	335.6
5	7.5	29.4	3.5	4	11.0	4	4	12.6	7	7	38.5	12	12	113.0	22.5	19	335.6
4.5	8	28.3	3.5	4	11.0	4	4	12.6	7	7	38.5	12	12	113.0	22.5	19	335.6
5.5	7.5	32.4	3.5	4	11.0	4	4	12.6	7	7	38.5	12	12	113.0	22.5	19	335.6
4.5	4.5	15.9	3.5	4	11.0	4	4	12.6	7	7	38.5	12	12	113.0	22.5	19	335.6
4	4.5	14.1	3.5	4	11.0	4	4	12.6	7	7	38.5	12	12	113.0	22.5	19	335.6
4.5	4.5	15.9	3.5	4	11.0	4	4	12.6	7	7.5	41.2	12	12	113.0	22.5	19	335.6
3.5	4.5	12.4	3.5	4	11.0	4	4	12.6	7	7	38.5	12	12	113.0	22.5	19	335.6
4.5	3.5	12.4	3.5	4	11.0	4	4	12.6	7	7	38.5	12	12	113.0	22.5	19	335.6
4	4	12.6	3.5	4	11.0	4	4	12.6	7	7	38.5	12	12	113.0	22.5	19	335.6
4.5	4	14.1	3.5	4	11.0	5.5	5	21.6	7	7.5	41.2	12	12	113.0	15	15	176.6
4.5	4.5	15.9	3.5	4	11.0	5.5	5	21.6	7	7	38.5	12	12	113.0	15	15	176.6
4	3.5	11.0	3.5	4	11.0	5.5	5	21.6	7	7	38.5	12	12	113.0	15	15	176.6
3.5	4	11.0	3.5	4	11.0	5.5	5	21.6	7	7	38.5	12	12	113.0	15	15	176.6
4	4	12.6	3.5	4	11.0	5.5	5	21.6	7	7	38.5	12	12	113.0	15	15	176.6
4.5	3.5	12.4	3.5	4	11.0	5.5	5	21.6	7	7	38.5	9	8.5	60.1	15	15	176.6
4	4.5	14.1	3.5	4	11.0	5.5	5	21.6	7	7	38.5	9	8.5	60.1	15	15	176.6
4.5	4	14.1	3	3	7.1	5.5	5	21.6	6	6	28.3	9	8.5	60.1	15	15	176.6
4.5	4.5	15.9	3	3	7.1	5.5	5	21.6	6	6	28.3	9	8.5	60.1	15	15	176.6
4	3.5	11.0	3	3	7.1	5.5	5	21.6	6	6	28.3	9	8.5	60.1	15	15	176.6
3.5	4	11.0	3	3	7.1	5.5	5	21.6	6	6	28.3	9	8.5	60.1	15	15	176.6
4.5	4.5	15.9	3	3	7.1	5.5	5	21.6	6	6	28.3	9	8.5	60.1	15	15	176.6
4.5	4.5	15.9	3	3	7.1	5.5	5	21.6	6	6	28.3	9	8.5	60.1	15	15	176.6
4	4	12.6	3	3	7.1	5.5	5	21.6	6	6	28.3	9	8.5	60.1	15	15	176.6
3.5	3.5	9.6	3	3	7.1	5.5	5	21.6	6	6	28.3	9	8.5	60.1	15	15	176.6
3.5	4.5	12.4	3	3	7.1	5.5	5	21.6	6	6	28.3	9	8.5	60.1	15	15	176.6
4	3.5	11.0	3	3	7.1	5.5	5	21.6	6	6	28.3	9	8.5	60.1	15	15	176.6
4.5	3.5	12.4	3	3	7.1	5.5	5	21.6	6	6	28.3	9	8.5	60.1	15	15	176.6
4	3.5	11.0	3	3	7.1	5.5	5	21.6	6	6.5	30.6	9	8.5	60.1	15	15	176.6
4.5	4	14.1	3	3	7.1	5.5	5	21.6	6	6.5	30.6	9	9	63.6	15	15	176.6
3.5	4.5	12.4	3	3	7.1	5.5	5	21.6	6	6.5	30.6	9	9	63.6	15	15	176.6
3.5	4	11.0	3	3	7.1	5.5	5	21.6	6	6.5	30.6	9	9	63.6	15	15	176.6

3.5	4	11.0	3	3	7.1	5.5	5	21.6	6	6.5	30.6	9	9	63.6	15	15	176.6
4.5	4	14.1	3	3	7.1	5.5	5	21.6	6	6.5	30.6	9	9	63.6	15	15	176.6
4.5	4.5	15.9	3	3	7.1	5.5	5	21.6	6	6.5	30.6	9	9	63.6	15	15	176.6
4	4	12.6	3	3	7.1	5.5	5	21.6	6	6.5	30.6	9	9	63.6	15	15	176.6
3.5	3.5	9.6	3	3	7.1	5.5	5	21.6	6	6.5	30.6	9	9	63.6	15	15	176.6
4.5	4.5	15.9	3	3	7.1	5.5	5	21.6	6	6.5	30.6	9	9	63.6	15	15	176.6
4	4.5	14.1	3	3	7.1	5.5	5	21.6	6	6.5	30.6	9	9	63.6	15	15	176.6
4.5	5	17.7	3	3.5	8.2	5.5	5	21.6	6	6.5	30.6	9	9	63.6	15	15	176.6
3.5	5	13.7	3	3.5	8.2	5.5	5	21.6	6	6.5	30.6	9	9	63.6	15	15	176.6
3.5	4	11.0	3	3.5	8.2	5.5	5	21.6	6	6.5	30.6	9	9	63.6	15	15	176.6
4.5	4.5	15.9	3	3.5	8.2	5.5	5	21.6	6	6.5	30.6	9	9	63.6	15	15	176.6
4	5	15.7	3	3.5	8.2	5.5	5	21.6	6	6.5	30.6	9	9	63.6	15	15	176.6
4	5	15.7	3	3.5	8.2	5.5	5	21.6	6	6.5	30.6	9	9	63.6	15	15	176.6
3.5	4.5	12.4	3	3.5	8.2	5.5	5	21.6	5	5	19.6	12.5	12.5	122.7	15	15	176.6
4.5	4	14.1	3	3.5	8.2	5.5	5	21.6	5	5	19.6	12.5	12.5	122.7	15	15	176.6
4	4.5	14.1	3	3.5	8.2	5.5	5	21.6	5	5	19.6	12.5	12.5	122.7	15	15	176.6
4	5	15.7	3	3.5	8.2	5.5	5	21.6	5	5	19.6	12.5	12.5	122.7	15	15	176.6
4.5	3	10.6	3	3.5	8.2	5.5	5	21.6	5	5	19.6	12.5	12.5	122.7	15	15	176.6
4.5	5	17.7	3	3.5	8.2	5.5	5	21.6	5	5	19.6	12.5	12.5	122.7	12.5	13.5	132.5
6	4.5	21.2	3	3.5	8.2	5.5	5	21.6	5	5	19.6	12.5	12.5	122.7	12.5	13.5	132.5
5	5	19.6	3	3.5	8.2	5.5	5	21.6	5	5	19.6	12.5	12.5	122.7	12.5	13.5	132.5
4.5	5	17.7	3	3.5	8.2	5.5	5	21.6	5	5	19.6	12.5	12.5	122.7	12.5	13.5	132.5
4	4	12.6	3	3.5	8.2	5.5	5	21.6	5	5	19.6	12.5	12.5	122.7	12.5	13.5	132.5
5	4.5	17.7	3	3.5	8.2	5.5	5	21.6	5	5	19.6	12.5	12.5	122.7	12.5	13.5	132.5
4.5	4.5	15.9	3	3.5	8.2	5.5	5	21.6	5	5	19.6	12.5	12.5	122.7	12.5	13.5	132.5
3.5	3.5	9.6	4	4.5	14.1	5.5	5	21.6	5	5	19.6	12.5	12.5	122.7	12.5	13.5	132.5
5.5	4.5	19.4	4	4.5	14.1	5.5	5	21.6	2.5	2.5	4.9	12.5	12.5	122.7	12.5	13.5	132.5
4.5	4.5	15.9	4	4.5	14.1	5.5	5	21.6	2.5	2.5	4.9	12.5	12.5	122.7	12.5	13.5	132.5
4	4	12.6	4	4.5	14.1	5.5	5	21.6	2.5	2.5	4.9	12	12.5	117.8	12.5	13.5	132.5
4	4	12.6	4	4.5	14.1	5.5	5	21.6	2.5	2.5	4.9	12	12.5	117.8	12.5	13.5	132.5
4	4	12.6	4	4.5	14.1	5.5	5	21.6	2.5	2.5	4.9	12	12.5	117.8	12.5	13.5	132.5
4	4	12.6	4	4.5	14.1	5.5	5	21.6	2.5	2.5	4.9	12	12.5	117.8	12.5	13.5	132.5
4	4	12.6	4	4.5	14.1	5.5	5	21.6	2.5	2.5	4.9	12	12.5	117.8	12.5	13.5	132.5
4	4	12.6	4	4.5	14.1	5.5	5	21.6	2.5	2.5	4.9	12	12.5	117.8	12.5	13.5	132.5
4	4.5	14.1	4	4.5	14.1	5.5	5	21.6	2.5	2.5	4.9	12	12.5	117.8	12.5	13.5	132.5
4	4	12.6	4	4.5	14.1	5.5	5	21.6	2.5	2.5	4.9	12	12.5	117.8	12.5	13.5	132.5

4	4	12.6	4	4.5	14.1	5.5	5	21.6	2.5	2.5	4.9	12	12.5	117.8	12.5	13.5	132.5
4	3.5	11.0	4	4.5	14.1	5.5	5	21.6	2.5	2.5	4.9	12	12.5	117.8	12.5	13.5	132.5
4	4	12.6	4	4.5	14.1	5.5	5	21.6	2.5	2.5	4.9	2.5	2.5	4.9	9	9	63.6
4	4	12.6	4	4.5	14.1	5.5	5	21.6	2.5	2.5	4.9	2.5	2.5	4.9	9	9	63.6
4	3	9.4	4	4.5	14.1	5.5	5	21.6	2.5	2.5	4.9	2.5	2.5	4.9	9	9	63.6
4	4	12.6	4	4.5	14.1	5.5	5	21.6	2.5	2.5	4.9	2.5	2.5	4.9	9	9	63.6
5.5	6	25.9	4	4.5	14.1	5.5	5	21.6	2.5	2.5	4.9	2.5	2.5	4.9	9	9	63.6
5.5	5.5	23.7	4	4.5	14.1	5.5	5	21.6	2.5	2.5	4.9	2.5	2.5	4.9	9	9	63.6
5.5	5.5	23.7	4	4.5	14.1	5.5	5	21.6	2.5	2.5	4.9	2.5	2.5	4.9	9	9	63.6
6	5.5	25.9	4	4.5	14.1	5.5	5	21.6	2.5	2.5	4.9	2.5	2.5	4.9	9	9	63.6
6	6	28.3	4	4.5	14.1	5.5	5	21.6	2.5	2.5	4.9	2.5	2.5	4.9	9	9	63.6
6	6	28.3	4	4.5	14.1	5.5	5	21.6	2.5	2.5	4.9	2.5	2.5	4.9	9	9	63.6
6	5.5	25.9	4	4.5	14.1	5.5	5	21.6	2.5	3	5.9	2.5	2.5	4.9	9	9	63.6
6	6	28.3	4	4.5	14.1	5.5	5	21.6	2.5	3	5.9	2.5	2.5	4.9	9	9	63.6
5.5	5.5	23.7	4.5	4.5	15.9	5.5	5	21.6	2.5	3	5.9	2.5	2.5	4.9	9	9	63.6
5.5	5.5	23.7	4.5	4.5	15.9	5.5	5	21.6	2.5	3	5.9	2.5	2.5	4.9	9	9	63.6
4.5	6	21.2	4.5	4.5	15.9	5.5	5	21.6	2.5	3	5.9	2.5	2.5	4.9	9	9	63.6
4.5	6	21.2	4.5	4.5	15.9	5.5	5	21.6	2.5	3	5.9	2.5	2.5	4.9	9	9	63.6
4.5	7.5	26.5	4.5	4.5	15.9	5.5	5	21.6	2.5	3	5.9	2.5	2.5	4.9	9	9	63.6
4.5	7.5	26.5	4.5	4.5	15.9	5.5	5	21.6	2.5	3	5.9	2.5	2.5	4.9	9	9	63.6
4.5	8	28.3	4.5	4.5	15.9	5.5	5	21.6	2.5	3	5.9	2.5	2.5	4.9	9	9	63.6
4.5	8	28.3	4.5	4.5	15.9	5.5	5	21.6	2.5	3	5.9	2.5	2.5	4.9	4	4	12.6
4.5	7.5	26.5	4.5	4.5	15.9	5.5	5	21.6	2.5	3	5.9	2.5	2.5	4.9	4	4	12.6
4.5	7.5	26.5	4.5	4.5	15.9	5.5	5	21.6	2.5	3	5.9	2.5	2.5	4.9	4	4	12.6
4.5	8	28.3	4.5	4.5	15.9	5.5	5	21.6	2.5	3	5.9	2.5	2.5	4.9	4	4	12.6
4.5	8	28.3	4.5	4.5	15.9	5.5	5	21.6	2.5	3	5.9	2.5	2.5	4.9	4	4	12.6
4.5	8	28.3	4.5	4.5	15.9	6	5	23.6	2.5	3	5.9	2.5	2.5	4.9	4	4	12.6
4.5	7.5	26.5	4.5	4.5	15.9	6	5	23.6	2.5	3	5.9	2.5	2.5	4.9	4	4	12.6
4.5	7	24.7	4.5	4.5	15.9	6	5	23.6	4	4	12.6	2.5	2.5	4.9	4	4	12.6
4.5	7	24.7	4.5	4.5	15.9	6	5	23.6	4	4	12.6	2.5	2.5	4.9	4	4	12.6
4.5	7.5	26.5	4.5	4.5	15.9	6	5	23.6	4	4	12.6	2.5	2.5	4.9	4	4	12.6
4.5	7	24.7	4.5	4	14.1	6	5	23.6	4	4	12.6	2.5	2.5	4.9	4	4	12.6
4.5	7.5	26.5	4.5	4	14.1	6	5	23.6	4	4	12.6	2.5	2.5	4.9	10	9	70.7
4.5	8	28.3	4.5	4	14.1	6	5	23.6	4	4	12.6	3	2.5	5.9	10	9	70.7
4.5	8	28.3	4.5	4	14.1	6	5	23.6	4	4	12.6	3	2.5	5.9	10	9	70.7
4.5	8	28.3	4.5	4	14.1	6	5	23.6	4	4	12.6	3	2.5	5.9	10	9	70.7

4.5	7.5	26.5	4.5	4	14.1	6	5	23.6	4	4	12.6	3	2.5	5.9	10	9	70.7
4.5	7.5	26.5	4.5	4	14.1	6	5	23.6	4	4	12.6	3	2.5	5.9	10	9	70.7
4.5	8	28.3	4.5	4	14.1	6	5	23.6	4	4	12.6	3	2.5	5.9	10	9	70.7
4.5	8	28.3	4.5	4	14.1	6	5	23.6	4	4	12.6	3	2.5	5.9	10	9	70.7
4.5	7	24.7	4.5	4	14.1	6	5	23.6	4	4	12.6	3	2.5	5.9	10	9	70.7
4.5	7	24.7	4.5	4	14.1	6	5	23.6	4	4	12.6	3	2.5	5.9	10	9	70.7
4.5	7.5	26.5	4.5	4	14.1	7.5	5	29.4	4	4	12.6	3	2.5	5.9	10	9	70.7
4.5	8	28.3	4.5	4	14.1	7.5	5	29.4	4	4	12.6	3	2.5	5.9	10	9	70.7
4.5	7	24.7	4.5	4	14.1	7.5	5	29.4	4	4	12.6	3	2.5	5.9	10	9	70.7
4.5	7	24.7	4.5	4	14.1	7.5	5	29.4	4	4	12.6	3	2.5	5.9	10	9	70.7
4.5	7.5	26.5	4.5	4	14.1	7.5	5	29.4	4	4	12.6	3	2.5	5.9	10	9	70.7
4.5	8	28.3	4.5	4	14.1	7.5	5	29.4	4	4	12.6	3	2.5	5.9	10	9	70.7
4.5	7	24.7	4.5	4	14.1	7.5	5	29.4	4	4	12.6	3	2.5	5.9	10	9	70.7
4.5	7.5	26.5	4.5	4	14.1	7.5	5	29.4	4	4	12.6	3	2.5	5.9	10	9	70.7
4	3	9.4	4	4	12.6	7.5	5	29.4	4	4	12.6	3	2.5	5.9	10	9	70.7
4	3	9.4	4	4	12.6	7.5	5	29.4	4	4	12.6	3	2.5	5.9	10	9	70.7
4	3	9.4	4	4	12.6	7.5	5	29.4	4	4	12.6	3	2.5	5.9	10	9	70.7
4	3	9.4	4	4	12.6	7.5	5	29.4	4	4	12.6	3	2.5	5.9	10	9	70.7
4	3	9.4	4	4	12.6	7.5	5	29.4	4	4	12.6	2	2	3.1	10	9	70.7
4	3	9.4	4	4	12.6	7.5	5	29.4	4	4	12.6	2	2	3.1	10	9	70.7
4	3	9.4	4	4	12.6	7.5	5	29.4	4	4	12.6	2	2	3.1	10	9	70.7
4	3	9.4	4	4	12.6	7.5	5	29.4	4	4	12.6	2	2	3.1	10	9	70.7
4	3	9.4	4	4	12.6	7.5	5	29.4	4	4	12.6	2	2	3.1	10	9	70.7
4	3	9.4	4	4	12.6	7.5	5	29.4	4	4	12.6	2	2	3.1	10	22.5	176.6
3	3	7.1	4	4	12.6	7.5	5.5	32.4	4	4	12.6	2	2	3.1	10	22.5	176.6
3	3	7.1	4	4	12.6	7.5	5.5	32.4	4	4	12.6	2	2	3.1	10	22.5	176.6
4.5	4.5	15.9	4	4	12.6	7.5	5.5	32.4	4	4	12.6	2	2	3.1	10	22.5	176.6
4.5	4.5	15.9	4	4	12.6	7.5	5.5	32.4	4	4	12.6	2	2	3.1	10	22.5	176.6
4.5	4.5	15.9	4	4	12.6	7.5	5.5	32.4	4	4	12.6	2	2	3.1	10	22.5	176.6
4.5	4.5	15.9	4	4	12.6	7.5	5.5	32.4	4	4	12.6	2	2	3.1	10	22.5	176.6
4.5	4.5	15.9	4	4	12.6	7.5	5.5	32.4	4	4	12.6	2	2	3.1	10	22.5	176.6
4.5	4.5	15.9	4	4	12.6	7.5	5.5	32.4	4	4	12.6	2	2	3.1	10	22.5	176.6
4.5	4.5	15.9	4	4	12.6	7.5	5.5	32.4	4	4	12.6	2	2	3.1	25	22.5	441.6
4.5	4.5	15.9	4	4	12.6	7.5	5.5	32.4	4	4	12.6	2	2	3.1	25	22.5	441.6
4.5	4	14.1	4	4	12.6	7.5	5.5	32.4	4	4	12.6	2	2	3.1	25	22.5	441.6
4.5	4.5	15.9	4	4	12.6	7.5	5.5	32.4	4	4	12.6	2	2	3.1	25	22.5	441.6

4.5	4.5	15.9	4	3.5	11.0	7.5	5.5	32.4	4	4	12.6	2	2	3.1	25	22.5	441.6
4	4.5	14.1	4	3.5	11.0	7.5	5.5	32.4	4	4	12.6	2	2	3.1	25	22.5	441.6
4.5	3.5	12.4	4	3.5	11.0	7.5	5.5	32.4	4	4	12.6	2	14.5	22.8	25	22.5	441.6
4.5	4.5	15.9	4	3.5	11.0	7.5	5.5	32.4	4	4	12.6	2	14.5	22.8	12.5	10	98.1
4.5	4.5	15.9	4	3.5	11.0	7.5	5.5	32.4	4	4	12.6	2	14.5	22.8	12.5	10	98.1
4.5	4.5	15.9	4	3.5	11.0	7.5	5.5	32.4	4	4	12.6	2	14.5	22.8	12.5	10	98.1
4.5	4.5	15.9	4	3.5	11.0	7.5	5.5	32.4	4	4	12.6	2	14.5	22.8	12.5	10	98.1
3.5	3	8.2	4	3.5	11.0	7.5	5.5	32.4	4	4	12.6	2	14.5	22.8	12.5	10	98.1
3.5	3.5	9.6	4	3.5	11.0	7.5	5.5	32.4	4	4	12.6	2	14.5	22.8	12.5	10	98.1
3.5	3.5	9.6	4	3.5	11.0	7.5	5.5	32.4	4	4	12.6	2	14.5	22.8	12.5	10	98.1
3.5	3.5	9.6	4	3.5	11.0	6.5	5	25.5	4	4	12.6	15	14.5	170.7	12.5	10	98.1
4.5	3.5	12.4	4	3.5	11.0	6.5	5	25.5	4	4	12.6	15	14.5	170.7	12.5	10	98.1
4.5	3.5	12.4	4	3.5	11.0	6.5	5	25.5	4	4	12.6	15	14.5	170.7	12.5	10	98.1
4.5	3.5	12.4	4	3.5	11.0	6.5	5	25.5	4	4	12.6	15	14.5	170.7	12.5	10	98.1
4.5	3.5	12.4	4	3.5	11.0	6.5	5	25.5	4	3.5	11.0	15	14.5	170.7	12.5	10	98.1
4	3.5	11.0	4	3.5	11.0	6.5	5	25.5	4	3.5	11.0	14.5	14.5	165.0	12.5	10	98.1
4	3.5	11.0	4	3.5	11.0	6.5	5	25.5	4	3.5	11.0	14.5	14.5	165.0	9	9	63.6
4	3.5	11.0	4	3.5	11.0	6.5	5	25.5	4	3.5	11.0	14.5	14.5	165.0	9	9	63.6
4	3.5	11.0	4	3.5	11.0	6.5	5	25.5	4	3.5	11.0	14.5	14.5	165.0	9	9	63.6
4	3.5	11.0	4	3.5	11.0	6.5	5	25.5	4	3.5	11.0	14.5	14.5	165.0	9	9	63.6
4	3.5	11.0	4	3.5	11.0	6.5	5	25.5	4	3.5	11.0	14.5	14.5	165.0	9	9	63.6
4	3.5	11.0	4	3.5	11.0	6.5	5	25.5	4	3.5	11.0	14.5	14.5	165.0	9	9	63.6
4	3.5	11.0	4	3.5	11.0	6.5	5	25.5	4	3.5	11.0	14.5	14.5	165.0	9	9	63.6
4	3.5	11.0	4	3.5	11.0	6.5	5	25.5	4	3.5	11.0	14.5	14.5	165.0	9	9	63.6
4	3	9.4	4	3.5	11.0	6.5	5	25.5	3	3	7.1	14.5	14.5	165.0	9	9	63.6
3	3	7.1	4	3.5	11.0	6.5	5	25.5	3	3	7.1	14.5	14.5	165.0	9	9	63.6
3	3	7.1	4	3.5	11.0	6.5	5	25.5	3	3	7.1	14.5	14.5	165.0	9	9	63.6
3	3	7.1	4	3.5	11.0	6.5	5	25.5	3	3	7.1	14.5	14.5	165.0	9	9	63.6
4.5	4.5	15.9	4	3.5	11.0	6.5	5	25.5	3	3	7.1	14.5	14.5	165.0	9	9	63.6
4.5	4.5	15.9	4	3.5	11.0	6.5	5	25.5	3	3	7.1	14.5	14.5	165.0	9	9	63.6

Table G. Size of control and C1-treated *C. parapsilosis* ATCC 22019 cells after 24h, 48h and 72 h of culture. Results from this table were used for preparing Fig 4C and 4 F in the main body of paper.

Candida parapsilosis																							
Control 24 h				Control 48h				Control 72h				C1 16 µg/ml, 24h				C1 16 µg/ml, 48h				C1 16 µg/ml, 72h			
Diameter A [µm]	Diameter B [µm]	Cell size [µm^2]	A/B	Diameter A [µm]	Diameter B [µm]	Cell size [µm^2]	A/B	Diameter A [µm]	Diameter B [µm]	Cell size [µm^2]	A/B	Diameter A [µm]	Diameter B [µm]	Cell size [µm^2]	A/B	Diameter A [µm]	Diameter B [µm]	Cell size [µm^2]	A/B	Diameter A [µm]	Diameter B [µm]	Cell size [µm^2]	A/B
25	3	58.9	8.3	20	5	78.5	4.0	30	4	94.2	7.5	10	4	31.4	2.5	5	5	19.6	1.0	27.5	26.5	572.1	1.0
25	3	58.9	8.3	20	5	78.5	4.0	30	4	94.2	7.5	10	4	31.4	2.5	5	5	19.6	1.0	27.5	26.5	572.1	1.0
25	3	58.9	8.3	20	5	78.5	4.0	30	4	94.2	7.5	10	4	31.4	2.5	5	5	19.6	1.0	27.5	26.5	572.1	1.0
25	3	58.9	8.3	20	5	78.5	4.0	30	4	94.2	7.5	10	4	31.4	2.5	5	5	19.6	1.0	27.5	26.5	572.1	1.0
25	3	58.9	8.3	20	5	78.5	4.0	30	4	94.2	7.5	10	4	31.4	2.5	5	5	19.6	1.0	27.5	26.5	572.1	1.0
25	3	58.9	8.3	20	5	78.5	4.0	30	4	94.2	7.5	10	4	31.4	2.5	5	5	19.6	1.0	27.5	26.5	572.1	1.0
25	3	58.9	8.3	20	5	78.5	4.0	30	4	94.2	7.5	10	4	31.4	2.5	5	5	19.6	1.0	27.5	26.5	572.1	1.0
25	3	58.9	8.3	20	5	78.5	4.0	30	4	94.2	7.5	10	4	31.4	2.5	5	5	19.6	1.0	27.5	26.5	572.1	1.0
25	3	58.9	8.3	20	5	78.5	4.0	30	4	94.2	7.5	10	4	31.4	2.5	5	5	19.6	1.0	27.5	26.5	572.1	1.0
25	3	58.9	8.3	20	5	78.5	4.0	30	4	94.2	7.5	10	4	31.4	2.5	5	5	19.6	1.0	27.5	26.5	572.1	1.0
25	3	58.9	8.3	20	5	78.5	4.0	30	4	94.2	7.5	10	4	31.4	2.5	5	5	19.6	1.0	27.5	26.5	572.1	1.0
25	3	58.9	8.3	20	5	78.5	4.0	30	4	94.2	7.5	10	4	31.4	2.5	5	5	19.6	1.0	27.5	26.5	572.1	1.0
25	3	58.9	8.3	20	5	78.5	4.0	30	4	94.2	7.5	10	4	31.4	2.5	5	5	19.6	1.0	27.5	26.5	572.1	1.0
25	3	58.9	8.3	20	5	78.5	4.0	30	4	94.2	7.5	10	4	31.4	2.5	5	5	19.6	1.0	27.5	26.5	572.1	1.0
25	3	58.9	8.3	20	5	78.5	4.0	30	4	94.2	7.5	10	4	31.4	2.5	5	5	19.6	1.0	27.5	26.5	572.1	1.0
25	3	58.9	8.3	20	5	78.5	4.0	30	4	94.2	7.5	10	4	31.4	2.5	5	5	19.6	1.0	27.5	26.5	572.1	1.0
25	3	58.9	8.3	20	5	78.5	4.0	30	4	94.2	7.5	10	4	31.4	2.5	5	5	19.6	1.0	27.5	26.5	572.1	1.0
25	3	58.9	8.3	20	5	78.5	4.0	30	4	94.2	7.5	10	4	31.4	2.5	5	5	19.6	1.0	27.5	26.5	572.1	1.0
25	3	58.9	8.3	20	5	78.5	4.0	30	4	94.2	7.5	10	4	31.4	2.5	5	5	19.6	1.0	27.5	26.5	572.1	1.0
25	3	58.9	8.3	20	5	78.5	4.0	30	4	94.2	7.5	10	4	31.4	2.5	5	5	19.6	1.0	27.5	26.5	572.1	1.0
25	3	58.9	8.3	20	5	78.5	4.0	30	4	94.2	7.5	10	4	31.4	2.5	5	5	19.6	1.0	27.5	26.5	572.1	1.0
25	3	58.9	8.3	20	5	78.5	4.0	30	4	94.2	7.5	10	4	31.4	2.5	5	5	19.6	1.0	27.5	26.5	572.1	1.0
20	3	47.1	6.7	20	5	78.5	4.0	30	4	94.2	7.5	10	4	31.4	2.5	5	5	19.6	1.0	27.5	26.5	572.1	1.0
20	3	47.1	6.7	20	5	78.5	4.0	30	4	94.2	7.5	10	4	31.4	2.5	5	5	19.6	1.0	20	20.5	321.9	1.0
20	3	47.1	6.7	20	5	78.5	4.0	30	4	94.2	7.5	10	4	31.4	2.5	5	5	19.6	1.0	20	20.5	321.9	1.0
30	4	94.2	7.5	20	5	78.5	4.0	30	4	94.2	7.5	10	4	31.4	2.5	5	5	19.6	1.0	20	20.5	321.9	1.0
30	4	94.2	7.5	20	5	78.5	4.0	30	4	94.2	7.5	10	4	31.4	2.5	20	20	314.0	1.0	20	20.5	321.9	1.0

30	4	94.2	7.5	30	4	94.2	7.5	30	4	94.2	7.5	7.5	5	29.4	1.5	20	20	314.0	1.0	20	20.5	321.9	1.0
30	4	94.2	7.5	30	4	94.2	7.5	25	5	98.1	5.0	7.5	5	29.4	1.5	20	20	314.0	1.0	20	20.5	321.9	1.0
30	4	94.2	7.5	30	4	94.2	7.5	25	5	98.1	5.0	7.5	5	29.4	1.5	20	20	314.0	1.0	20	20.5	321.9	1.0
30	4	94.2	7.5	30	4	94.2	7.5	25	5	98.1	5.0	7.5	5	29.4	1.5	20	20	314.0	1.0	20	20.5	321.9	1.0
30	4	94.2	7.5	30	4	94.2	7.5	25	5	98.1	5.0	7.5	5	29.4	1.5	20	20	314.0	1.0	20	20.5	321.9	1.0
30	4	94.2	7.5	30	4	94.2	7.5	25	5	98.1	5.0	7.5	5	29.4	1.5	20	20	314.0	1.0	20	20.5	321.9	1.0
30	4	94.2	7.5	30	4	94.2	7.5	25	5	98.1	5.0	7.5	5	29.4	1.5	20	20	314.0	1.0	20	20.5	321.9	1.0
30	4	94.2	7.5	30	4	94.2	7.5	25	5	98.1	5.0	7.5	5	29.4	1.5	20	20	314.0	1.0	20	20.5	321.9	1.0
30	4	94.2	7.5	30	4	94.2	7.5	25	5	98.1	5.0	7.5	5	29.4	1.5	20	20	314.0	1.0	20	20.5	321.9	1.0
30	4	94.2	7.5	30	4	94.2	7.5	25	5	98.1	5.0	7.5	5	29.4	1.5	20	20	314.0	1.0	20	20.5	321.9	1.0
30	4	94.2	7.5	30	4	94.2	7.5	25	5	98.1	5.0	7.5	5	29.4	1.5	20	20	314.0	1.0	20	20.5	321.9	1.0
30	4	94.2	7.5	30	4	94.2	7.5	25	5	98.1	5.0	7.5	5	29.4	1.5	20	20	314.0	1.0	20	20.5	321.9	1.0
30	4	94.2	7.5	30	4	94.2	7.5	25	5	98.1	5.0	7.5	5	29.4	1.5	20	20	314.0	1.0	20	20.5	321.9	1.0
30	4	94.2	7.5	30	4	94.2	7.5	25	5	98.1	5.0	7.5	5	29.4	1.5	20	20	314.0	1.0	20	20.5	321.9	1.0
30	4	94.2	7.5	30	4	94.2	7.5	25	5	98.1	5.0	7.5	5	29.4	1.5	20	20	314.0	1.0	20	20.5	321.9	1.0
30	4	94.2	7.5	30	4	94.2	7.5	25	5	98.1	5.0	7.5	5	29.4	1.5	20	20	314.0	1.0	20	20.5	321.9	1.0
30	4	94.2	7.5	30	4	94.2	7.5	15	6	70.7	2.5	7.5	5	29.4	1.5	20	20	314.0	1.0	20	20.5	321.9	1.0
20	3	47.1	6.7	30	4	94.2	7.5	15	6	70.7	2.5	7.5	5	29.4	1.5	20	20	314.0	1.0	20	20.5	321.9	1.0
20	3	47.1	6.7	30	4	94.2	7.5	15	6	70.7	2.5	7.5	5	29.4	1.5	20	20	314.0	1.0	20	20.5	321.9	1.0
20	3	47.1	6.7	12.5	4	39.3	3.1	15	6	70.7	2.5	7.5	5	29.4	1.5	20	20	314.0	1.0	20	20.5	321.9	1.0
20	3	47.1	6.7	12.5	4	39.3	3.1	15	6	70.7	2.5	7.5	5	29.4	1.5	20	20	314.0	1.0	20	20.5	321.9	1.0
20	3	47.1	6.7	12.5	4	39.3	3.1	15	6	70.7	2.5	7.5	5	29.4	1.5	20	20	314.0	1.0	20	20.5	321.9	1.0
20	3	47.1	6.7	12.5	4	39.3	3.1	15	6	70.7	2.5	10	5	39.3	2.0	20	20	314.0	1.0	17.5	17.5	240.4	1.0
20	3	47.1	6.7	12.5	4	39.3	3.1	15	6	70.7	2.5	10	5	39.3	2.0	20	20	314.0	1.0	17.5	17.5	240.4	1.0
22.5	2.5	44.2	9.0	12.5	4	39.3	3.1	15	6	70.7	2.5	10	5	39.3	2.0	20	20	314.0	1.0	17.5	17.5	240.4	1.0
22.5	2.5	44.2	9.0	12.5	4	39.3	3.1	15	6	70.7	2.5	10	5	39.3	2.0	20	20	314.0	1.0	17.5	17.5	240.4	1.0
22.5	2.5	44.2	9.0	12.5	4	39.3	3.1	15	6	70.7	2.5	10	5	39.3	2.0	20	20	314.0	1.0	17.5	17.5	240.4	1.0
22.5	2.5	44.2	9.0	12.5	4	39.3	3.1	15	6	70.7	2.5	10	5	39.3	2.0	20	20	314.0	1.0	17.5	17.5	240.4	1.0
22.5	2.5	44.2	9.0	12.5	4	39.3	3.1	15	6	70.7	2.5	10	5	39.3	2.0	20	20	314.0	1.0	17.5	17.5	240.4	1.0
22.5	2.5	44.2	9.0	12.5	4	39.3	3.1	15	6	70.7	2.5	10	5	39.3	2.0	20	20	314.0	1.0	17.5	17.5	240.4	1.0
22.5	2.5	44.2	9.0	12.5	4	39.3	3.1	15	6	70.7	2.5	10	5	39.3	2.0	20	20	314.0	1.0	17.5	17.5	240.4	1.0
22.5	2.5	44.2	9.0	12.5	4	39.3	3.1	15	6	70.7	2.5	10	5	39.3	2.0	20	20	314.0	1.0	17.5	17.5	240.4	1.0
22.5	2.5	44.2	9.0	12.5	4	39.3	3.1	15	6	70.7	2.5	10	5	39.3	2.0	20	20	314.0	1.0	17.5	17.5	240.4	1.0
22.5	2.5	44.2	9.0	12.5	4	39.3	3.1	15	6	70.7	2.5	10	5	39.3	2.0	20	20	314.0	1.0	17.5	17.5	240.4	1.0
22.5	2.5	44.2	9.0	12.5	4	39.3	3.1	22.5	2.5	44.2	9.0	10	5	39.3	2.0	20	20	314.0	1.0	17.5	17.5	240.4	1.0
22.5	2.5	44.2	9.0	12.5	4	39.3	3.1	22.5	2.5	44.2	9.0	10	5	39.3	2.0	20	20	314.0	1.0	17.5	17.5	240.4	1.0

22.5	2.5	44.2	9.0	12.5	4	39.3	3.1	22.5	2.5	44.2	9.0	10	5	39.3	2.0	20	20	314.0	1.0	17.5	17.5	240.4	1.0
22.5	2.5	44.2	9.0	12.5	4	39.3	3.1	22.5	2.5	44.2	9.0	10	5	39.3	2.0	17.5	17.5	240.4	1.0	17.5	17.5	240.4	1.0
22.5	2.5	44.2	9.0	25	3.5	68.7	7.1	22.5	2.5	44.2	9.0	10	5	39.3	2.0	17.5	17.5	240.4	1.0	17.5	17.5	240.4	1.0
22.5	2.5	44.2	9.0	25	3.5	68.7	7.1	22.5	2.5	44.2	9.0	10	5	39.3	2.0	17.5	17.5	240.4	1.0	17.5	17.5	240.4	1.0
22.5	2.5	44.2	9.0	25	3.5	68.7	7.1	22.5	2.5	44.2	9.0	10	5	39.3	2.0	17.5	17.5	240.4	1.0	17.5	17.5	240.4	1.0
22.5	2.5	44.2	9.0	25	3.5	68.7	7.1	22.5	2.5	44.2	9.0	10	5	39.3	2.0	17.5	17.5	240.4	1.0	17.5	17.5	240.4	1.0
22.5	2.5	44.2	9.0	25	3.5	68.7	7.1	22.5	2.5	44.2	9.0	10	5	39.3	2.0	17.5	17.5	240.4	1.0	17.5	17.5	240.4	1.0
22.5	2.5	44.2	9.0	25	3.5	68.7	7.1	22.5	2.5	44.2	9.0	10	5	39.3	2.0	17.5	17.5	240.4	1.0	17.5	17.5	240.4	1.0
22.5	2.5	44.2	9.0	25	3.5	68.7	7.1	22.5	2.5	44.2	9.0	10	5	39.3	2.0	17.5	17.5	240.4	1.0	10	9	70.7	1.1
22.5	2.5	44.2	9.0	25	3.5	68.7	7.1	22.5	2.5	44.2	9.0	7.5	7.5	44.2	1.0	17.5	17.5	240.4	1.0	10	9	70.7	1.1
22.5	2.5	44.2	9.0	25	3.5	68.7	7.1	22.5	2.5	44.2	9.0	7.5	7.5	44.2	1.0	17.5	17.5	240.4	1.0	10	9	70.7	1.1
22.5	2.5	44.2	9.0	25	3.5	68.7	7.1	22.5	2.5	44.2	9.0	7.5	7.5	44.2	1.0	17.5	17.5	240.4	1.0	10	9	70.7	1.1
22.5	2.5	44.2	9.0	25	3.5	68.7	7.1	22.5	2.5	44.2	9.0	7.5	7.5	44.2	1.0	17.5	17.5	240.4	1.0	10	9	70.7	1.1
22.5	2.5	44.2	9.0	25	3.5	68.7	7.1	22.5	2.5	44.2	9.0	7.5	7.5	44.2	1.0	17.5	17.5	240.4	1.0	10	9	70.7	1.1
15	2.5	29.4	6.0	25	3.5	68.7	7.1	22.5	2.5	44.2	9.0	7.5	7.5	44.2	1.0	17.5	17.5	240.4	1.0	10	9	70.7	1.1
15	2.5	29.4	6.0	25	3.5	68.7	7.1	22.5	2.5	44.2	9.0	7.5	7.5	44.2	1.0	17.5	17.5	240.4	1.0	10	9	70.7	1.1
15	2.5	29.4	6.0	22.5	5	88.3	4.5	22.5	2.5	44.2	9.0	7.5	7.5	44.2	1.0	17.5	17.5	240.4	1.0	10	9	70.7	1.1
15	2.5	29.4	6.0	22.5	5	88.3	4.5	22.5	2.5	44.2	9.0	7.5	7.5	44.2	1.0	17.5	17.5	240.4	1.0	10	9	70.7	1.1
15	2.5	29.4	6.0	22.5	5	88.3	4.5	22.5	2.5	44.2	9.0	7.5	7.5	44.2	1.0	17.5	17.5	240.4	1.0	10	9	70.7	1.1
15	2.5	29.4	6.0	22.5	5	88.3	4.5	22.5	2.5	44.2	9.0	7.5	7.5	44.2	1.0	17.5	17.5	240.4	1.0	10	9	70.7	1.1
15	2.5	29.4	6.0	22.5	5	88.3	4.5	22.5	2.5	44.2	9.0	7.5	7.5	44.2	1.0	17.5	17.5	240.4	1.0	10	9	70.7	1.1
15	2.5	29.4	6.0	22.5	5	88.3	4.5	22.5	2.5	44.2	9.0	7.5	7.5	44.2	1.0	17.5	17.5	240.4	1.0	10	9	70.7	1.1
15	2.5	29.4	6.0	22.5	5	88.3	4.5	22.5	2.5	44.2	9.0	7.5	7.5	44.2	1.0	17.5	17.5	240.4	1.0	10	9	70.7	1.1
15	2.5	29.4	6.0	22.5	5	88.3	4.5	22.5	2.5	44.2	9.0	7.5	7.5	44.2	1.0	17.5	17.5	240.4	1.0	10	9	70.7	1.1
15	2.5	29.4	6.0	22.5	5	88.3	4.5	22.5	2.5	44.2	9.0	7.5	7.5	44.2	1.0	17.5	17.5	240.4	1.0	10	9	70.7	1.1
15	2.5	29.4	6.0	22.5	5	88.3	4.5	7.5	4	23.6	1.9	7.5	7.5	44.2	1.0	17.5	17.5	240.4	1.0	10	9	70.7	1.1
15	2.5	29.4	6.0	22.5	5	88.3	4.5	7.5	4	23.6	1.9	7.5	7.5	44.2	1.0	17.5	17.5	240.4	1.0	10	9	70.7	1.1
15	2.5	29.4	6.0	22.5	5	88.3	4.5	7.5	4	23.6	1.9	7.5	7.5	44.2	1.0	17.5	17.5	240.4	1.0	10	9	70.7	1.1
15	2.5	29.4	6.0	22.5	5	88.3	4.5	7.5	4	23.6	1.9	7.5	7.5	44.2	1.0	17.5	17.5	240.4	1.0	10	9	70.7	1.1
15	2.5	29.4	6.0	22.5	5	88.3	4.5	7.5	4	23.6	1.9	7.5	7.5	44.2	1.0	17.5	17.5	314.0	1.0	10	9	70.7	1.1
25	4.5	88.3	5.6	22.5	5	88.3	4.5	7.5	4	23.6	1.9	7.5	7.5	44.2	1.0	17.5	17.5	240.4	1.0	10	9	70.7	1.1
25	4.5	88.3	5.6	15	2.5	29.4	6.0	7.5	4	23.6	1.9	7.5	7.5	44.2	1.0	17.5	17.5	240.4	1.0	10	9	70.7	1.1
25	4.5	88.3	5.6	15	2.5	29.4	6.0	7.5	4	23.6	1.9	7.5	7.5	44.2	1.0	17.5	17.5	240.4	1.0	10	9	70.7	1.1
25	4.5	88.3	5.6	15	2.5	29.4	6.0	7.5	4	23.6	1.9	7.5	7.5	44.2	1.0	17.5	17.5	240.4	1.0	10	9	70.7	1.1
25	4.5	88.3	5.6	15	2.5	29.4	6.0	7.5	4	23.6	1.9	7.5	7.5	44.2	1.0	17.5	17.5	240.4	1.0	17	16	213.5	1.1
25	4.5	88.3	5.6	15	2.5	29.4	6.0	7.5	4	23.6	1.9	7.5	7.5	44.2	1.0	17.5	17.5	240.4	1.0	17	16	213.5	1.1

25	4.5	88.3	5.6	15	2.5	29.4	6.0	7.5	4	23.6	1.9	7.5	7.5	44.2	1.0	17.5	17.5	240.4	1.0	17	16	213.5	1.1
25	4.5	88.3	5.6	15	2.5	29.4	6.0	7.5	4	23.6	1.9	10	7.5	58.9	1.3	17.5	17.5	240.4	1.0	17	16	213.5	1.1
25	4.5	88.3	5.6	15	2.5	29.4	6.0	7.5	4	23.6	1.9	10	7.5	58.9	1.3	17.5	17.5	240.4	1.0	17	16	213.5	1.1
25	4.5	88.3	5.6	15	2.5	29.4	6.0	7.5	4	23.6	1.9	10	7.5	58.9	1.3	17.5	17.5	240.4	1.0	17	16	213.5	1.1
25	4.5	88.3	5.6	15	2.5	29.4	6.0	7.5	4	23.6	1.9	10	7.5	58.9	1.3	12.5	13	127.6	1.0	17	16	213.5	1.1
25	4.5	88.3	5.6	15	2.5	29.4	6.0	7.5	4	23.6	1.9	10	7.5	58.9	1.3	12.5	13	127.6	1.0	17	16	213.5	1.1
25	4.5	88.3	5.6	15	2.5	29.4	6.0	7.5	4	23.6	1.9	10	7.5	58.9	1.3	12.5	13	127.6	1.0	17	16	213.5	1.1
25	4.5	88.3	5.6	15	2.5	29.4	6.0	7.5	4	23.6	1.9	10	7.5	58.9	1.3	12.5	13	127.6	1.0	17	16	213.5	1.1
25	4.5	88.3	5.6	15	2.5	29.4	6.0	7.5	4	23.6	1.9	10	7.5	58.9	1.3	12.5	13	127.6	1.0	17	16	213.5	1.1
25	4.5	88.3	5.6	15	2.5	29.4	6.0	7.5	4	23.6	1.9	10	7.5	58.9	1.3	12.5	13	127.6	1.0	17	16	213.5	1.1
25	4.5	88.3	5.6	15	2.5	29.4	6.0	7.5	4	23.6	1.9	10	7.5	58.9	1.3	12.5	13	127.6	1.0	17	16	213.5	1.1
25	4.5	88.3	5.6	15	2.5	29.4	6.0	7.5	4	23.6	1.9	10	7.5	58.9	1.3	12.5	13	127.6	1.0	17	16	213.5	1.1
25	4.5	88.3	5.6	15	2.5	29.4	6.0	7.5	4	23.6	1.9	10	7.5	58.9	1.3	12.5	13	127.6	1.0	17	16	213.5	1.1
25	4.5	88.3	5.6	15	2.5	29.4	6.0	7.5	4	23.6	1.9	10	7.5	58.9	1.3	12.5	13	127.6	1.0	17	16	213.5	1.1
25	4.5	88.3	5.6	15	2.5	29.4	6.0	20	2.5	39.3	8.0	10	7.5	58.9	1.3	12.5	13	127.6	1.0	17	16	213.5	1.1
25	4.5	88.3	5.6	15	2.5	29.4	6.0	20	2.5	39.3	8.0	10	7.5	58.9	1.3	12.5	13	127.6	1.0	17	16	213.5	1.1
25	4.5	88.3	5.6	15	2.5	29.4	6.0	20	2.5	39.3	8.0	10	7.5	58.9	1.3	12.5	13	127.6	1.0	17	16	213.5	1.1
25	4.5	88.3	5.6	15	2.5	29.4	6.0	20	2.5	39.3	8.0	10	7.5	58.9	1.3	12.5	13	127.6	1.0	17	16	213.5	1.1
25	4.5	88.3	5.6	15	2.5	29.4	6.0	20	2.5	39.3	8.0	10	7.5	58.9	1.3	12.5	13	127.6	1.0	17	16	213.5	1.1
25	4.5	88.3	5.6	15	2.5	29.4	6.0	20	2.5	39.3	8.0	10	7.5	58.9	1.3	12.5	13	127.6	1.0	17	16	213.5	1.1
25	4.5	88.3	5.6	15	2.5	29.4	6.0	20	2.5	39.3	8.0	10	7.5	58.9	1.3	12.5	13	127.6	1.0	17	16	213.5	1.1
25	4.5	88.3	5.6	15	2.5	29.4	6.0	20	2.5	39.3	8.0	10	7.5	58.9	1.3	12.5	13	127.6	1.0	17	16	213.5	1.1
25	4.5	88.3	5.6	15	2.5	29.4	6.0	20	2.5	39.3	8.0	10	7.5	58.9	1.3	12.5	13	127.6	1.0	7.5	7.5	44.2	1.0
25	5	98.1	5.0	20	3	47.1	6.7	20	2.5	39.3	8.0	7.5	9	53.0	0.8	12.5	13	127.6	1.0	7.5	7.5	44.2	1.0
25	5	98.1	5.0	20	3	47.1	6.7	20	2.5	39.3	8.0	7.5	9	53.0	0.8	12.5	13	127.6	1.0	7.5	7.5	44.2	1.0
25	5	98.1	5.0	20	3	47.1	6.7	20	2.5	39.3	8.0	7.5	9	53.0	0.8	12.5	13	127.6	1.0	7.5	7.5	44.2	1.0
25	5	98.1	5.0	20	3	47.1	6.7	20	2.5	39.3	8.0	7.5	9	53.0	0.8	12.5	13	127.6	1.0	7.5	7.5	44.2	1.0
40	4	125.6	10.0	20	3	47.1	6.7	20	2.5	39.3	8.0	7.5	9	53.0	0.8	15	15	176.6	1.0	7.5	7.5	44.2	1.0
40	4	125.6	10.0	20	3	47.1	6.7	20	2.5	39.3	8.0	7.5	9	53.0	0.8	15	15	176.6	1.0	7.5	7.5	44.2	1.0
40	4	125.6	10.0	20	3	47.1	6.7	20	2.5	39.3	8.0	7.5	9	53.0	0.8	15	15	176.6	1.0	7.5	7.5	44.2	1.0
40	4	125.6	10.0	20	3	47.1	6.7	20	2.5	39.3	8.0	7.5	9	53.0	0.8	15	15	176.6	1.0	7.5	7.5	44.2	1.0
40	4	125.6	10.0	20	3	47.1	6.7	20	2.5	39.3	8.0	7.5	9	53.0	0.8	15	15	176.6	1.0	7.5	7.5	44.2	1.0
40	4	125.6	10.0	20	3	47.1	6.7	20	2.5	39.3	8.0	7.5	9	53.0	0.8	15	15	176.6	1.0	7.5	7.5	44.2	1.0
40	4	125.6	10.0	20	3	47.1	6.7	20	2.5	39.3	8.0	7.5	9	53.0	0.8	15	15	176.6	1.0	7.5	7.5	44.2	1.0
40	4	125.6	10.0	20	3	47.1	6.7	20	2.5	39.3	8.0	7.5	9	53.0	0.8	15	15	176.6	1.0	7.5	7.5	44.2	1.0
40	4	125.6	10.0	20	3	47.1	6.7	20	2.5	39.3	8.0	7.5	9	53.0	0.8	15	15	176.6	1.0	7.5	7.5	44.2	1.0

40	4	125.6	10.0	20	3	47.1	6.7	20	2.5	39.3	8.0	7.5	9	53.0	0.8	15	15	176.6	1.0	7.5	7.5	44.2	1.0
40	4	125.6	10.0	20	3	47.1	6.7	25	4.5	88.3	5.6	7.5	9	53.0	0.8	15	15	176.6	1.0	7.5	7.5	44.2	1.0
40	4	125.6	10.0	20	3	47.1	6.7	25	4.5	88.3	5.6	7.5	9	53.0	0.8	15	15	176.6	1.0	7.5	7.5	44.2	1.0
40	4	125.6	10.0	20	3	47.1	6.7	25	4.5	88.3	5.6	7.5	9	53.0	0.8	15	15	176.6	1.0	7.5	7.5	44.2	1.0
40	4	125.6	10.0	20	3	47.1	6.7	25	4.5	88.3	5.6	7.5	9	53.0	0.8	15	15	176.6	1.0	7.5	7.5	44.2	1.0
40	4	125.6	10.0	20	3	47.1	6.7	25	4.5	88.3	5.6	7.5	9	53.0	0.8	15	15	176.6	1.0	7.5	7.5	44.2	1.0
40	4	125.6	10.0	20	3	47.1	6.7	25	4.5	88.3	5.6	7.5	9	53.0	0.8	15	15	176.6	1.0	7.5	7.5	44.2	1.0
40	4	125.6	10.0	20	3	47.1	6.7	25	4.5	88.3	5.6	7.5	9	53.0	0.8	15	15	176.6	1.0	7.5	7.5	44.2	1.0
40	4	125.6	10.0	20	3	47.1	6.7	25	4.5	88.3	5.6	7.5	9	53.0	0.8	15	15	176.6	1.0	7.5	7.5	44.2	1.0
22.5	5	88.3	4.5	20	3	47.1	6.7	25	4.5	88.3	5.6	7.5	9	53.0	0.8	15	15	176.6	1.0	7.5	7.5	44.2	1.0
22.5	5	88.3	4.5	20	3	47.1	6.7	25	4.5	88.3	5.6	9	4	28.3	2.3	15	15	176.6	1.0	7.5	7.5	44.2	1.0
22.5	5	88.3	4.5	20	3	47.1	6.7	25	4.5	88.3	5.6	9	4	28.3	2.3	15	17.5	206.1	0.9	7.5	7.5	44.2	1.0
22.5	5	88.3	4.5	20	3	47.1	6.7	25	4.5	88.3	5.6	9	4	28.3	2.3	15	17.5	206.1	0.9	7.5	7.5	44.2	1.0
22.5	5	88.3	4.5	20	3	47.1	6.7	25	4.5	88.3	5.6	9	4	28.3	2.3	15	17.5	206.1	0.9	10	2.5	19.6	4.0
22.5	5	88.3	4.5	20	3	47.1	6.7	25	4.5	88.3	5.6	9	4	28.3	2.3	15	17.5	206.1	0.9	10	2.5	19.6	4.0
22.5	5	88.3	4.5	20	3	47.1	6.7	25	4.5	88.3	5.6	9	4	28.3	2.3	15	17.5	206.1	0.9	10	2.5	19.6	4.0
22.5	5	88.3	4.5	20	3	47.1	6.7	25	4.5	88.3	5.6	9	4	28.3	2.3	15	17.5	206.1	0.9	10	2.5	19.6	4.0
22.5	5	88.3	4.5	20	3	47.1	6.7	25	4.5	88.3	5.6	9	4	28.3	2.3	15	17.5	206.1	0.9	10	2.5	19.6	4.0
22.5	5	88.3	4.5	30	4	94.2	7.5	25	4.5	88.3	5.6	9	4	28.3	2.3	15	17.5	206.1	0.9	10	2.5	19.6	4.0
20	3	47.1	6.7	30	4	94.2	7.5	25	4.5	88.3	5.6	9	4	28.3	2.3	15	17.5	206.1	0.9	10	2.5	19.6	4.0
20	3	47.1	6.7	30	4	94.2	7.5	25	4.5	88.3	5.6	9	4	28.3	2.3	15	17.5	206.1	0.9	10	2.5	19.6	4.0
20	3	47.1	6.7	30	4	94.2	7.5	25	4.5	88.3	5.6	9	4	28.3	2.3	15	17.5	206.1	0.9	10	2.5	19.6	4.0
20	3	47.1	6.7	30	4	94.2	7.5	25	4.5	88.3	5.6	9	4	28.3	2.3	15	17.5	206.1	0.9	10	2.5	19.6	4.0
20	3	47.1	6.7	30	4	94.2	7.5	25	4.5	88.3	5.6	9	4	28.3	2.3	15	17.5	206.1	0.9	10	2.5	19.6	4.0
20	3	47.1	6.7	30	4	94.2	7.5	30	4	94.2	7.5	9	4	28.3	2.3	15	17.5	206.1	0.9	10	2.5	19.6	4.0
20	3	47.1	6.7	30	4	94.2	7.5	30	4	94.2	7.5	9	4	28.3	2.3	15	17.5	206.1	0.9	10	2.5	19.6	4.0
25	3.5	68.7	7.1	30	4	94.2	7.5	30	4	94.2	7.5	9	4	28.3	2.3	15	17.5	206.1	0.9	10	2.5	19.6	4.0
25	3.5	68.7	7.1	30	4	94.2	7.5	30	4	94.2	7.5	9	4	28.3	2.3	15	17.5	206.1	0.9	10	2.5	19.6	4.0
25	3.5	68.7	7.1	30	4	94.2	7.5	30	4	94.2	7.5	9	4	28.3	2.3	15	17.5	206.1	0.9	10	2.5	19.6	4.0
25	3.5	68.7	7.1	30	4	94.2	7.5	30	4	94.2	7.5	9	4	28.3	2.3	15	17.5	206.1	0.9	10	2.5	19.6	4.0
25	3.5	68.7	7.1	30	4	94.2	7.5	30	4	94.2	7.5	7.5	7.5	44.2	1.0	15	17.5	206.1	0.9	10	2.5	19.6	4.0
25	3.5	68.7	7.1	30	4	94.2	7.5	30	4	94.2	7.5	7.5	7.5	44.2	1.0	15	17.5	206.1	0.9	10	2.5	19.6	4.0
25	3.5	68.7	7.1	30	4	94.2	7.5	30	4	94.2	7.5	7.5	7.5	44.2	1.0	15	17.5	206.1	0.9	10	2.5	19.6	4.0
25	3.5	68.7	7.1	30	4	94.2	7.5	30	4	94.2	7.5	7.5	7.5	44.2	1.0	15	17.5	206.1	0.9	10	2.5	19.6	4.0
25	3.5	68.7	7.1	30	4	94.2	7.5	30	4	94.2	7.5	7.5	7.5	44.2	1.0	15	17.5	206.1	0.9	10	2.5	19.6	4.0
25	3.5	68.7	7.1	30	4	94.2	7.5	30	4	94.2	7.5	7.5	7.5	44.2	1.0	15	17.5	206.1	0.9	10	2.5	19.6	4.0

25	3.5	68.7	7.1	30	4	94.2	7.5	30	4	94.2	7.5	7.5	7.5	44.2	1.0	15	17.5	206.1	0.9	12.5	12.5	122.7	1.0
25	3.5	68.7	7.1	30	4	94.2	7.5	30	4	94.2	7.5	7.5	7.5	44.2	1.0	15	17.5	206.1	0.9	12.5	12.5	122.7	1.0
25	6	117.8	4.2	30	4	94.2	7.5	30	4	94.2	7.5	7.5	7.5	44.2	1.0	10	2.5	19.6	4.0	12.5	12.5	122.7	1.0
15	6	70.7	2.5	30	4	94.2	7.5	30	4	94.2	7.5	7.5	7.5	44.2	1.0	10	2.5	19.6	4.0	12.5	12.5	122.7	1.0
15	6	70.7	2.5	30	4	94.2	7.5	30	4	94.2	7.5	7.5	7.5	44.2	1.0	10	2.5	19.6	4.0	12.5	12.5	122.7	1.0
15	6	70.7	2.5	30	4	94.2	7.5	30	4	94.2	7.5	7.5	7.5	44.2	1.0	10	2.5	19.6	4.0	12.5	12.5	122.7	1.0
15	6	70.7	2.5	30	4	94.2	7.5	30	4	94.2	7.5	7.5	7.5	44.2	1.0	10	2.5	19.6	4.0	12.5	12.5	122.7	1.0
15	6	70.7	2.5	30	4	94.2	7.5	30	4	94.2	7.5	7.5	7.5	44.2	1.0	10	2.5	19.6	4.0	12.5	12.5	122.7	1.0
15	6	70.7	2.5	30	4	94.2	7.5	30	4	94.2	7.5	7.5	7.5	44.2	1.0	10	2.5	19.6	4.0	12.5	12.5	122.7	1.0
15	6	70.7	2.5	30	4	94.2	7.5	30	4	94.2	7.5	7.5	7.5	44.2	1.0	10	2.5	19.6	4.0	12.5	12.5	122.7	1.0
15	6	70.7	2.5	30	4	94.2	7.5	30	4	94.2	7.5	7.5	7.5	44.2	1.0	10	2.5	19.6	4.0	12.5	12.5	122.7	1.0
15	6	70.7	2.5	30	4	94.2	7.5	40	4	125.6	10.0	7.5	7.5	44.2	1.0	10	2.5	19.6	4.0	12.5	12.5	122.7	1.0
7.5	4	23.6	1.9	30	4	94.2	7.5	40	4	125.6	10.0	7.5	7.5	44.2	1.0	10	2.5	19.6	4.0	12.5	12.5	122.7	1.0
7.5	4	23.6	1.9	25	5	98.1	5.0	40	4	125.6	10.0	10	5	39.3	2.0	10	2.5	19.6	4.0	12.5	12.5	122.7	1.0
7.5	4	23.6	1.9	25	5	98.1	5.0	40	4	125.6	10.0	10	5	39.3	2.0	10	2.5	19.6	4.0	12.5	12.5	122.7	1.0
7.5	4	23.6	1.9	25	5	98.1	5.0	40	4	125.6	10.0	10	5	39.3	2.0	15	15	176.6	1.0	12.5	12.5	122.7	1.0
7.5	4	23.6	1.9	25	5	98.1	5.0	40	4	125.6	10.0	10	5	39.3	2.0	15	15	176.6	1.0	12.5	12.5	122.7	1.0
7.5	4	23.6	1.9	25	5	98.1	5.0	40	4	125.6	10.0	10	5	39.3	2.0	15	15	176.6	1.0	22.5	22.5	397.4	1.0
7.5	4	23.6	1.9	25	5	98.1	5.0	40	4	125.6	10.0	10	5	39.3	2.0	15	15	176.6	1.0	22.5	22.5	397.4	1.0
7.5	4	23.6	1.9	25	5	98.1	5.0	40	4	125.6	10.0	10	5	39.3	2.0	15	15	176.6	1.0	22.5	22.5	397.4	1.0
7.5	4	23.6	1.9	25	5	98.1	5.0	40	4	125.6	10.0	10	5	39.3	2.0	15	15	176.6	1.0	22.5	22.5	397.4	1.0
7.5	4	23.6	1.9	25	5	98.1	5.0	40	4	125.6	10.0	10	5	39.3	2.0	15	15	176.6	1.0	22.5	22.5	397.4	1.0
7.5	4	23.6	1.9	25	5	98.1	5.0	40	4	125.6	10.0	10	5	39.3	2.0	15	15	176.6	1.0	22.5	22.5	397.4	1.0
7.5	4	23.6	1.9	25	5	98.1	5.0	40	4	125.6	10.0	10	5	39.3	2.0	15	15	176.6	1.0	22.5	22.5	397.4	1.0
7.5	4	23.6	1.9	25	5	98.1	5.0	40	4	125.6	10.0	10	5	39.3	2.0	15	15	176.6	1.0	22.5	22.5	397.4	1.0
7.5	4	23.6	1.9	25	5	98.1	5.0	40	4	125.6	10.0	10	5	39.3	2.0	15	15	176.6	1.0	22.5	22.5	397.4	1.0
7.5	4	23.6	1.9	25	5	98.1	5.0	40	4	125.6	10.0	10	5	39.3	2.0	15	15	176.6	1.0	22.5	22.5	397.4	1.0
7.5	4	23.6	1.9	25	5	98.1	5.0	40	4	125.6	10.0	10	5	39.3	2.0	15	15	176.6	1.0	22.5	22.5	397.4	1.0
7.5	4	23.6	1.9	25	5	98.1	5.0	40	4	125.6	10.0	10	5	39.3	2.0	15	15	176.6	1.0	22.5	22.5	397.4	1.0

Table H. Size of *C. albicans* NCPF 3153 cells treated with different concentrations of the C1 compound after 48 h of culture. Results from this table were used for preparing Fig 4B in the main body of the paper.

***Candida albicans*, 48 h**																							
**Control**			**C1 0.5 μg/ml**			**C1 1 μg/ml**			**C1 2 μg/ml**			**C1 4 μg/ml**			**C1 8 μg/ml**			**C1 16 μg/ml**			**C1 32 μg/ml**		
Diameter A [μm]	Diameter B [μm]	Cell size [μm^2]	Diameter A [μm]	Diameter B [μm]	Cell size [μm^2]	Diameter A [μm]	Diameter B [μm]	Cell size [μm^2]	Diameter A [μm]	Diameter B [μm]	Cell size [μm^2]	Diameter A [μm]	Diameter B [μm]	Cell size [μm^2]	Diameter A [μm]	Diameter B [μm]	Cell size [μm^2]	Diameter A [μm]	Diameter B [μm]	Cell size [μm^2]	Diameter A [μm]	Diameter B [μm]	Cell size [μm^2]
3	3.5	8.2	4	4.5	14.1	4.5	4.5	15.9	4	4	12.6	4	4	12.6	4	4	12.6	4	4	12.6	10	10	78.5
4.5	4.5	15.9	4	4.5	14.1	4.5	4.5	15.9	4	4	12.6	4	4	12.6	4	4	12.6	4	4	12.6	10	10	78.5
4	5	15.7	4	4.5	14.1	4.5	4.5	15.9	4	4	12.6	4	4	12.6	4	4	12.6	4	4	12.6	10	10	78.5
4.5	3	10.6	4	4.5	14.1	4.5	4.5	15.9	4	4	12.6	4	4	12.6	4	4	12.6	4	4	12.6	10	10	78.5
4.5	5	17.7	4	4.5	14.1	4.5	4.5	15.9	4	4	12.6	4	4	12.6	4	4	12.6	4	4	12.6	10	10	78.5
4.5	4.5	15.9	4	4.5	14.1	4.5	4.5	15.9	4	4	12.6	4	4	12.6	4	4	12.6	4	4	12.6	10	10	78.5
5	5	19.6	4	4.5	14.1	4.5	4.5	15.9	4	4	12.6	4	4	12.6	4	4	12.6	4	4	12.6	10	10	78.5
4.5	5	17.7	4	4.5	14.1	4.5	4.5	15.9	4	4	12.6	4	4	12.6	4	4	12.6	4	4	12.6	10	10	78.5
4	4	12.6	4	4.5	14.1	4.5	4.5	15.9	4	4	12.6	4	4	12.6	4	4	12.6	4	4	12.6	10	10	78.5
5	4.5	17.7	4	4.5	14.1	4.5	4.5	15.9	4	4	12.6	4	4	12.6	4	4	12.6	4	4	12.6	10	10	78.5
4.5	4.5	15.9	4	4.5	14.1	4.5	4.5	15.9	4	4	12.6	4	4	12.6	4	4	12.6	4	4	12.6	10	10	78.5
3.5	3.5	9.6	4	4.5	14.1	4.5	4.5	15.9	4	4	12.6	4	4	12.6	4	4	12.6	4	4	12.6	10	10	78.5
5.5	4.5	19.4	4	4.5	14.1	4.5	4.5	15.9	4	4	12.6	4	4	12.6	4	4	12.6	4	4	12.6	10	10	78.5
4.5	4.5	15.9	4	4.5	14.1	4.5	4.5	15.9	4	4	12.6	4	4	12.6	4	4	12.6	4	4	12.6	10	10	78.5
4	4	12.6	4	4.5	14.1	4.5	4.5	15.9	4	4	12.6	4	4	12.6	4	4	12.6	4	4	12.6	10	10	78.5
3	4	9.4	4	4.5	14.1	4.5	4.5	15.9	4	4	12.6	4	4	12.6	4	4	12.6	4	4	12.6	10	10	78.5
3	5	11.8	4	4.5	14.1	4.5	4.5	15.9	4	4	12.6	4	4	12.6	4	4	12.6	4	4	12.6	10	10	78.5
5.5	7.5	32.4	4	4.5	14.1	4.5	4.5	15.9	4	4	12.6	6	6.5	30.6	4	4	12.6	4	4	12.6	10	10	78.5
5	6.5	25.5	4	4.5	14.1	4.5	4.5	15.9	4	4	12.6	6	6.5	30.6	4	4	12.6	4	4	12.6	10	10	78.5
5	6.5	25.5	4	4.5	14.1	4.5	4.5	15.9	4	4	12.6	6	6.5	30.6	4	4	12.6	4	4	12.6	12	12	113.0
5	6	23.6	4	4.5	14.1	4.5	4.5	15.9	4	4	12.6	6	6.5	30.6	4	4	12.6	4	4	12.6	12	12	113.0
4.5	6	21.2	3.5	4.5	12.4	4.5	4.5	15.9	4	4	12.6	6	6.5	30.6	4	4	12.6	4.5	5	17.7	12	12	113.0
4.5	7.5	26.5	3.5	4.5	12.4	4.5	4.5	15.9	4	4	12.6	6	6.5	30.6	4	4	12.6	4.5	5	17.7	12	12	113.0
5.5	5.5	23.7	3.5	4.5	12.4	4.5	4.5	15.9	4	4	12.6	6	6.5	30.6	4	4	12.6	4.5	5	17.7	12	12	113.0
5	7	27.5	3.5	4.5	12.4	4.5	4.5	15.9	4	4.5	14.1	6	6.5	30.6	4	4	12.6	4.5	5	17.7	12	12	113.0
4.5	5	17.7	3.5	4.5	12.4	4.5	4	14.1	4	4.5	14.1	6	6.5	30.6	4	4	12.6	4.5	5	17.7	12	12	113.0

5.5	5	21.6	3.5	4.5	12.4	4.5	4	14.1	4	4.5	14.1	6	6.5	30.6	4	4	12.6	4.5	5	17.7	12	12	113.0
4.5	6	21.2	3.5	4.5	12.4	4.5	4	14.1	7	7	38.5	6	6.5	30.6	4	4	12.6	4.5	5	17.7	12	12	113.0
4.5	6.5	23.0	3.5	4.5	12.4	4.5	4	14.1	7	7	38.5	6	6.5	30.6	4	4	12.6	4.5	5	17.7	12	12	113.0
4.5	7	24.7	3.5	4.5	12.4	4.5	4	14.1	7	7	38.5	6	6.5	30.6	4	4	12.6	4.5	5	17.7	12	12	113.0
4.5	7	24.7	3.5	4.5	12.4	4.5	4	14.1	7	7	38.5	6	4.5	21.2	4	4	12.6	4.5	5	17.7	12	12	113.0
5	7.5	29.4	3.5	4.5	12.4	4.5	4	14.1	7	7	38.5	6	4.5	21.2	4	4	12.6	4.5	5	17.7	12	12	113.0
4.5	8	28.3	3.5	4.5	12.4	4.5	4	14.1	7	7	38.5	6	4.5	21.2	4	4.5	14.1	4.5	5	17.7	12	12	113.0
5.5	7.5	32.4	3.5	4.5	12.4	4.5	4	14.1	7	7	38.5	6	4.5	21.2	4	4.5	14.1	4.5	5	17.7	12	12	113.0
4.5	4.5	15.9	3.5	4.5	12.4	4.5	4	14.1	7	7	38.5	6	4.5	21.2	4	4.5	14.1	4.5	5	17.7	12	12	113.0
4	4.5	14.1	3.5	4.5	12.4	4.5	4	14.1	7	7	38.5	6	4.5	21.2	4	4.5	14.1	4.5	5	17.7	12	12	113.0
4.5	4.5	15.9	3.5	4.5	12.4	4.5	4	14.1	7	7	38.5	6	4.5	21.2	4	4.5	14.1	4.5	5	17.7	12	12	113.0
3.5	4.5	12.4	3.5	4.5	12.4	4.5	4	14.1	7	7	38.5	6	4.5	21.2	4	4.5	14.1	4.5	5	17.7	12	12	113.0
4.5	3.5	12.4	3.5	4.5	12.4	4.5	4	14.1	7	7	38.5	6	4.5	21.2	4	4.5	14.1	4.5	5	17.7	12	12	113.0
4	4	12.6	3.5	4.5	12.4	5.5	6	25.9	7	7	38.5	6	4.5	21.2	4	4.5	14.1	4.5	5	17.7	12	12	113.0
4.5	4	14.1	3.5	4.5	12.4	5.5	6	25.9	7	7	38.5	6	4.5	21.2	4	4.5	14.1	4.5	5	17.7	12	12	113.0
4.5	4.5	15.9	3.5	4.5	12.4	5.5	6	25.9	7	7	38.5	6	4.5	21.2	4	4.5	14.1	4.5	5	17.7	12	12	113.0
4	3.5	11.0	3.5	4.5	12.4	5.5	6	25.9	7	7	38.5	6	4.5	21.2	4	4.5	14.1	4.5	5	17.7	12	12	113.0
3.5	4	11.0	3.5	4.5	12.4	5.5	6	25.9	7	7	38.5	6	4.5	21.2	4	4.5	14.1	4.5	5	17.7	12	12	113.0
4	4	12.6	3.5	4.5	12.4	5.5	6	25.9	7	6.5	35.7	6	4.5	21.2	4	4.5	14.1	5.5	5	21.6	12	12	113.0
4.5	3.5	12.4	3.5	4.5	12.4	5.5	6	25.9	6.5	6.5	33.2	6	4.5	21.2	4	4.5	14.1	5.5	5	21.6	9	8.5	60.1
4	4.5	14.1	3.5	4.5	12.4	5.5	6	25.9	6.5	6.5	33.2	4	4.5	14.1	4	4.5	14.1	5.5	6	25.9	9	8.5	60.1
4.5	4	14.1	3.5	4.5	12.4	5.5	6	25.9	6.5	6.5	33.2	4	4.5	14.1	4	4.5	14.1	5.5	6	25.9	9	8.5	60.1
4.5	4.5	15.9	3.5	4.5	12.4	5.5	6	25.9	6.5	6.5	33.2	2.5	2.5	4.9	4	4.5	14.1	5.5	6	25.9	9	8.5	60.1
4	3.5	11.0	3.5	4.5	12.4	5.5	6	25.9	6.5	6.5	33.2	2.5	2.5	4.9	4	4.5	14.1	5.5	6	25.9	9	8.5	60.1
3.5	4	11.0	4	4	12.6	5.5	6	25.9	6.5	6.5	33.2	2.5	2.5	4.9	4	4.5	14.1	5.5	6	25.9	9	8.5	60.1
4.5	4.5	15.9	4	4	12.6	5.5	6	25.9	6.5	6.5	33.2	2.5	2.5	4.9	4	4.5	14.1	5.5	6	25.9	9	8.5	60.1
4.5	4.5	15.9	4	4	12.6	5.5	6	25.9	4	4.5	14.1	2.5	2.5	4.9	4	4.5	14.1	5.5	6	25.9	9	8.5	60.1
4	4	12.6	4	4	12.6	5.5	6	25.9	4	4.5	14.1	2.5	2.5	4.9	2	2.5	3.9	5.5	6	25.9	9	8.5	60.1
3.5	3.5	9.6	4	4	12.6	5.5	6	25.9	4	4.5	14.1	2.5	2.5	4.9	2	2.5	3.9	5.5	6	25.9	9	8.5	60.1
3.5	4.5	12.4	4	4	12.6	5.5	6	25.9	8	7.5	47.1	2.5	2.5	4.9	2	2.5	3.9	5.5	6	25.9	9	8.5	60.1
4	3.5	11.0	4	4	12.6	5.5	6	25.9	8	7.5	47.1	2.5	2.5	4.9	2	2.5	3.9	5.5	6	25.9	9	8.5	60.1
4.5	3.5	12.4	4	4	12.6	5.5	6	25.9	8	7.5	47.1	2.5	2.5	4.9	2	2.5	3.9	5.5	6	25.9	9	8.5	60.1
4	3.5	11.0	4	4	12.6	5.5	6	25.9	8	7.5	47.1	2.5	2.5	4.9	2	2.5	3.9	5.5	6	25.9	9	8.5	60.1
4.5	4	14.1	4	4	12.6	5.5	6	25.9	8	7.5	47.1	2.5	2.5	4.9	2	2.5	3.9	5.5	6	25.9	9	9	63.6
3.5	4.5	12.4	4	4	12.6	5.5	6	25.9	8	7.5	47.1	2.5	2.5	4.9	2	2.5	3.9	5.5	6	25.9	9	9	63.6
3.5	4	11.0	4	4	12.6	5.5	6	25.9	8	7.5	47.1	2.5	2.5	4.9	2	2.5	3.9	5.5	6	25.9	9	9	63.6

3.5	4	11.0	4	4	12.6	5.5	6	25.9	8	7.5	47.1	2.5	2.5	4.9	2	2.5	3.9	5.5	6	25.9	9	9	63.6
4.5	4	14.1	4	4	12.6	5.5	6	25.9	8	7.5	47.1	9	10	70.7	16	17.5	219.8	5.5	6	25.9	9	9	63.6
4.5	4.5	15.9	4	4	12.6	5.5	6	25.9	8	7.5	47.1	9	10	70.7	16	17.5	219.8	5.5	6	25.9	9	9	63.6
4	4	12.6	4	4	12.6	5.5	6	25.9	8	7.5	47.1	9	10	70.7	16	17.5	219.8	5.5	6	25.9	9	9	63.6
3.5	3.5	9.6	4	4	12.6	5.5	6	25.9	8	7.5	47.1	9	10	70.7	16	17.5	219.8	3	3	7.1	9	9	63.6
4.5	4.5	15.9	4	4	12.6	5.5	6.5	28.1	8	7.5	47.1	9	10	70.7	16	17.5	219.8	3	3	7.1	9	9	63.6
4	4.5	14.1	4	4	12.6	5.5	6.5	28.1	8	7.5	47.1	9	10	70.7	16	17.5	219.8	3	3	7.1	9	9	63.6
4.5	5	17.7	5	5.5	21.6	5.5	6.5	28.1	8	7.5	47.1	9	10	70.7	16	17.5	219.8	3	3	7.1	9	9	63.6
3.5	5	13.7	5	5.5	21.6	5.5	6.5	28.1	8	7.5	47.1	9	10	70.7	16	17.5	219.8	3	3	7.1	9	9	63.6
3.5	4	11.0	5	5.5	21.6	5.5	6.5	28.1	8	7.5	47.1	9	10	70.7	16	17.5	219.8	3	3	7.1	9	9	63.6
4.5	4.5	15.9	5	5.5	21.6	5.5	6.5	28.1	8	7.5	47.1	9	10	70.7	16	17.5	219.8	3	3	7.1	9	9	63.6
4	5	15.7	5	5.5	21.6	5.5	6.5	28.1	8	7.5	47.1	9	10	70.7	16	17.5	219.8	3	3	7.1	9	9	63.6
4	5	15.7	5	5.5	21.6	5.5	6.5	28.1	8	7.5	47.1	9	10	70.7	16	17.5	219.8	3	3	7.1	9	9	63.6
3.5	4.5	12.4	5	5.5	21.6	5.5	6.5	28.1	8	7.5	47.1	9	10	70.7	16	17.5	219.8	3	3	7.1	12.5	12.5	122.7
4.5	4	14.1	5	5.5	21.6	5.5	6.5	28.1	8	7.5	47.1	9	10	70.7	16	17.5	219.8	3	3	7.1	12.5	12.5	122.7
4	4.5	14.1	5	5.5	21.6	5.5	6.5	28.1	3	4	9.4	9	10	70.7	16	17.5	219.8	3	3	7.1	12.5	12.5	122.7
4	5	15.7	5	5.5	21.6	5.5	6.5	28.1	3	4	9.4	9	10	70.7	16	17.5	219.8	3	3	7.1	12.5	12.5	122.7
4.5	3	10.6	5	5.5	21.6	5.5	6.5	28.1	3	4	9.4	9	10	70.7	16	17.5	219.8	3	3	7.1	12.5	12.5	122.7
4.5	5	17.7	5	5.5	21.6	5.5	6.5	28.1	5.5	6	25.9	9	10	70.7	16	17.5	219.8	3	3	7.1	12.5	12.5	122.7
6	4.5	21.2	5	5.5	21.6	5.5	6.5	28.1	5.5	6	25.9	9	10	70.7	16	17.5	219.8	3	3	7.1	12.5	12.5	122.7
5	5	19.6	5	5.5	21.6	5.5	6.5	28.1	5.5	6	25.9	9	10	70.7	16	17.5	219.8	2.5	3	5.9	12.5	12.5	122.7
4.5	5	17.7	5	5.5	21.6	5.5	6.5	28.1	5.5	6	25.9	9	10	70.7	16	17.5	219.8	2.5	3	5.9	12.5	12.5	122.7
4	4	12.6	5	5.5	21.6	5.5	6.5	28.1	5.5	6	25.9	9	10	70.7	16	17.5	219.8	2.5	3	5.9	12.5	12.5	122.7
5	4.5	17.7	5	5.5	21.6	6.5	6.5	33.2	5.5	6	25.9	9	10	70.7	16	17.5	219.8	2.5	3	5.9	12.5	12.5	122.7
4.5	4.5	15.9	5	5.5	21.6	6.5	6.5	33.2	5.5	6	25.9	9	10	70.7	2.5	2.5	4.9	2.5	3	5.9	12.5	12.5	122.7
3.5	3.5	9.6	5	5.5	21.6	6.5	6.5	33.2	5.5	6	25.9	9	10	70.7	2.5	2.5	4.9	2.5	3	5.9	12.5	12.5	122.7
5.5	4.5	19.4	5	5.5	21.6	6.5	6.5	33.2	5.5	6	25.9	9	10	70.7	2.5	2.5	4.9	2.5	3	5.9	12.5	12.5	122.7
4.5	4.5	15.9	5	5.5	21.6	6.5	6.5	33.2	5.5	6	25.9	9	10	70.7	2.5	2.5	4.9	2.5	3	5.9	12.5	12.5	122.7
4	4	12.6	5	5.5	21.6	6.5	6.5	33.2	5.5	6	25.9	2.5	3	5.9	2.5	2.5	4.9	2.5	3	5.9	12	12.5	117.8
4	4	12.6	5	5.5	21.6	6.5	6.5	33.2	5.5	6	25.9	2.5	3	5.9	2.5	2.5	4.9	2.5	3	5.9	12	12.5	117.8
4	4	12.6	5	5.5	21.6	6.5	6.5	33.2	5.5	6	25.9	2.5	3	5.9	2.5	2.5	4.9	2.5	3	5.9	12	12.5	117.8
4	4	12.6	5	5.5	21.6	6.5	6.5	33.2	5.5	6	25.9	2.5	3	5.9	2.5	2.5	4.9	2.5	3	5.9	12	12.5	117.8
4	4	12.6	5	5.5	21.6	6.5	6.5	33.2	5.5	6	25.9	2.5	3	5.9	2.5	2.5	4.9	2.5	3	5.9	12	12.5	117.8
4	4	12.6	5	5.5	21.6	6.5	6.5	33.2	5.5	6	25.9	2.5	3	5.9	2.5	2.5	4.9	2.5	3	5.9	12	12.5	117.8
4	4.5	14.1	5	5.5	21.6	6.5	6.5	33.2	5.5	6	25.9	2.5	3	5.9	2.5	2.5	4.9	2.5	3	5.9	12	12.5	117.8
4	4	12.6	5	5.5	21.6	6.5	6.5	33.2	5.5	6	25.9	2.5	3	5.9	2.5	2.5	4.9	2.5	3	5.9	12	12.5	117.8

4	4	12.6	3	3	7.1	6.5	6.5	33.2	3.5	3.5	9.6	2.5	3	5.9	2.5	2.5	4.9	2.5	3	5.9	12	12.5	117.8
4	3.5	11.0	4.5	6	21.2	6.5	6.5	33.2	3.5	3.5	9.6	2.5	3	5.9	2.5	2.5	4.9	7.5	8	47.1	12	12.5	117.8
4	4	12.6	4.5	6	21.2	6.5	6.5	33.2	3.5	3.5	9.6	2.5	3	5.9	2.5	2.5	4.9	7.5	8	47.1	2.5	2.5	4.9
4	4	12.6	4.5	6	21.2	6.5	6.5	33.2	3.5	3.5	9.6	2	2.5	3.9	2.5	2.5	4.9	7.5	8	47.1	2.5	2.5	4.9
4	3	9.4	4.5	6	21.2	6.5	6.5	33.2	4	3.5	11.0	2	2.5	3.9	2.5	2.5	4.9	7.5	8	47.1	2.5	2.5	4.9
4	4	12.6	4.5	6	21.2	6.5	6.5	33.2	4	3.5	11.0	2	2.5	3.9	2.5	2.5	4.9	7.5	8	47.1	2.5	2.5	4.9
5.5	6	25.9	4.5	6	21.2	6.5	6.5	33.2	4	3.5	11.0	2	2.5	3.9	2.5	2.5	4.9	7.5	8	47.1	2.5	2.5	4.9
5.5	5.5	23.7	4.5	6	21.2	6.5	6.5	33.2	4	3.5	11.0	2	2.5	3.9	2.5	2.5	4.9	7.5	8	47.1	2.5	2.5	4.9
5.5	5.5	23.7	4.5	6	21.2	6.5	6.5	33.2	4	3.5	11.0	2	2.5	3.9	9	8.5	60.1	7.5	8	47.1	2.5	2.5	4.9
6	5.5	25.9	4.5	6	21.2	6.5	6.5	33.2	4	3.5	11.0	2	2.5	3.9	9	8.5	60.1	7.5	8	47.1	2.5	2.5	4.9
6	6	28.3	4.5	6	21.2	6.5	6.5	33.2	4	3.5	11.0	2	2.5	3.9	9	8.5	60.1	7.5	8	47.1	2.5	2.5	4.9
6	6	28.3	4.5	6	21.2	6.5	6.5	33.2	4	3.5	11.0	9	9	63.6	9	8.5	60.1	7.5	8	47.1	2.5	2.5	4.9
6	5.5	25.9	4.5	6	21.2	6.5	6.5	33.2	4	3.5	11.0	9	9	63.6	9	8.5	60.1	7.5	8	47.1	2.5	2.5	4.9
6	6	28.3	4.5	6	21.2	9	8.5	60.1	4	3.5	11.0	9	9	63.6	9	8.5	60.1	7.5	8	47.1	2.5	2.5	4.9
5.5	5.5	23.7	4.5	6	21.2	9	8.5	60.1	4	3.5	11.0	9	9	63.6	9	8.5	60.1	7.5	8	47.1	2.5	2.5	4.9
5.5	5.5	23.7	4.5	6	21.2	9	8.5	60.1	6	7	33.0	9	9	63.6	9	8.5	60.1	7.5	8	47.1	2.5	2.5	4.9
4.5	6	21.2	4.5	5.5	19.4	9	8.5	60.1	6	7	33.0	9	9	63.6	9	8.5	60.1	7.5	8	47.1	2.5	2.5	4.9
4.5	6	21.2	4.5	5.5	19.4	9	8.5	60.1	6	7	33.0	9	9	63.6	9	8.5	60.1	7.5	7.5	44.2	2.5	2.5	4.9
4.5	7.5	26.5	4.5	5.5	19.4	9	8.5	60.1	6	7	33.0	9	9	63.6	9	8.5	60.1	7.5	7.5	44.2	2.5	2.5	4.9
4.5	7.5	26.5	4.5	5.5	19.4	9	8.5	60.1	6	7	33.0	9	9	63.6	9	8.5	60.1	7.5	7.5	44.2	2.5	2.5	4.9
4.5	8	28.3	4.5	5.5	19.4	9	8.5	60.1	6	7	33.0	9	9	63.6	9	8.5	60.1	7.5	7.5	44.2	2.5	2.5	4.9
4.5	8	28.3	4.5	5.5	19.4	9	8.5	60.1	6	7	33.0	9	9	63.6	9	8.5	60.1	7.5	7.5	44.2	2.5	2.5	4.9
4.5	7.5	26.5	4.5	5.5	19.4	9	8.5	60.1	6	7	33.0	9	9	63.6	9	8.5	60.1	7.5	7.5	44.2	2.5	2.5	4.9
4.5	7.5	26.5	4.5	3	10.6	9	8.5	60.1	6	7	33.0	9	9	63.6	9	8.5	60.1	7.5	7.5	44.2	2.5	2.5	4.9
4.5	8	28.3	4.5	3.5	12.4	9	8.5	60.1	6	7	33.0	9	9	63.6	9	8.5	60.1	7.5	7.5	44.2	2.5	2.5	4.9
4.5	8	28.3	3	3.5	8.2	9	8.5	60.1	6	7	33.0	9	9	63.6	9.5	10	74.6	7.5	7.5	44.2	2.5	2.5	4.9
4.5	8	28.3	3	3.5	8.2	9	8.5	60.1	6	7	33.0	9	9	63.6	9.5	10	74.6	7.5	7.5	44.2	2.5	2.5	4.9
4.5	7.5	26.5	3	3.5	8.2	9	8.5	60.1	6	7	33.0	9	9	63.6	9.5	10	74.6	10.5	10	82.4	2.5	2.5	4.9
4.5	7	24.7	3	3.5	8.2	9	8.5	60.1	6	7	33.0	9	9	63.6	9.5	10	74.6	10.5	10	82.4	2.5	2.5	4.9
4.5	7	24.7	3	3.5	8.2	9	8	56.5	6	7	33.0	9	9	63.6	9.5	10	74.6	10.5	10	82.4	2.5	2.5	4.9
4.5	7.5	26.5	3	3.5	8.2	9	8	56.5	6	7	33.0	6.5	6.5	33.2	9.5	10	74.6	10.5	10	82.4	2.5	2.5	4.9
4.5	7	24.7	3	3.5	8.2	9	8	56.5	6	7	33.0	6.5	6.5	33.2	9.5	10	74.6	10.5	10	82.4	2.5	2.5	4.9
4.5	7.5	26.5	3	3.5	8.2	9	8	56.5	6	7	33.0	6.5	6.5	33.2	9.5	10	74.6	10.5	10	82.4	2.5	2.5	4.9
4.5	8	28.3	3	3.5	8.2	9	8	56.5	6	7	33.0	6.5	6.5	33.2	9.5	10	74.6	10.5	10	82.4	3	2.5	5.9
4.5	8	28.3	3	3.5	8.2	9	8	56.5	6	7	33.0	6.5	6.5	33.2	9.5	10	74.6	10.5	10	82.4	3	2.5	5.9
4.5	8	28.3	3	3.5	8.2	9	8	56.5	6	7	33.0	6.5	6.5	33.2	9.5	10	74.6	10.5	10	82.4	3	2.5	5.9

4.5	7.5	26.5	3	3.5	8.2	9	8	56.5	6	7	33.0	6.5	6.5	33.2	9.5	10	74.6	10.5	10	82.4	3	2.5	5.9
4.5	7.5	26.5	3	3.5	8.2	9	8	56.5	6	5	23.6	6.5	6.5	33.2	9.5	10	74.6	10.5	10	82.4	3	2.5	5.9
4.5	8	28.3	3	3.5	8.2	9	9	63.6	5	5.5	21.6	6.5	6.5	33.2	9.5	10	74.6	10.5	10	82.4	3	2.5	5.9
4.5	8	28.3	3	3.5	8.2	9	9	63.6	5	5.5	21.6	6.5	6.5	33.2	9.5	10	74.6	10.5	10	82.4	3	2.5	5.9
4.5	7	24.7	3	3.5	8.2	9	9	63.6	5	5.5	21.6	6.5	6.5	33.2	9.5	10	74.6	10.5	10	82.4	3	2.5	5.9
4.5	7	24.7	3	3.5	8.2	9	9	63.6	5	5.5	21.6	6	7	33.0	9.5	10	74.6	10.5	10	82.4	3	2.5	5.9
4.5	7.5	26.5	3	3.5	8.2	9	9	63.6	5	5.5	21.6	6	7	33.0	9.5	10	74.6	10.5	10	82.4	3	2.5	5.9
4.5	8	28.3	3	3.5	8.2	9	9	63.6	5	5.5	21.6	6	7	33.0	9.5	10	74.6	10.5	10	82.4	3	2.5	5.9
4.5	7	24.7	3	3.5	8.2	9	9	63.6	5	5.5	21.6	6	7	33.0	9.5	10	74.6	10.5	10	82.4	3	2.5	5.9
4.5	7	24.7	3	3.5	8.2	9	9	63.6	5	5.5	21.6	6	7	33.0	9.5	10	74.6	13	10	102.1	3	2.5	5.9
4.5	7.5	26.5	3	3.5	8.2	9	9	63.6	5	5.5	21.6	6	7	33.0	9.5	10	74.6	13	12	122.5	3	2.5	5.9
4.5	8	28.3	3	3.5	8.2	4.5	4	14.1	5	5.5	21.6	6	7	33.0	9.5	10	74.6	13	12	122.5	3	2.5	5.9
4.5	7	24.7	4	3.5	11.0	4.5	4	14.1	5	5.5	21.6	6	7	33.0	8	7.5	47.1	13	12	122.5	3	2.5	5.9
4.5	7.5	26.5	4	3.5	11.0	4.5	4	14.1	5	5.5	21.6	6	7	33.0	8	7.5	47.1	13	12	122.5	3	2.5	5.9
4	3	9.4	4	3.5	11.0	4.5	4	14.1	5	5.5	21.6	6	7	33.0	8	7.5	47.1	13	12	122.5	3	2.5	5.9
4	3	9.4	4	3.5	11.0	4.5	4	14.1	5	5.5	21.6	6	7	33.0	8	7.5	47.1	13	12	122.5	3	2.5	5.9
4	3	9.4	4	3.5	11.0	4.5	4	14.1	5	5.5	21.6	6	7	33.0	8	7.5	47.1	13	12	122.5	3	2.5	5.9
4	3	9.4	4	3.5	11.0	4.5	4	14.1	9	9	63.6	6	7	33.0	8	7.5	47.1	13	12	122.5	3	2.5	5.9
4	3	9.4	4	3.5	11.0	4.5	4	14.1	9	9	63.6	6	7	33.0	8	8	50.2	13	12	122.5	2	2	3.1
4	3	9.4	4	3.5	11.0	4.5	4	14.1	9	9	63.6	6	7	33.0	8	8	50.2	13	12	122.5	21	21	346.2
4	3	9.4	4	3.5	11.0	4.5	4	14.1	9	9	63.6	6	7	33.0	8	8	50.2	13	12	122.5	21	21	346.2
4	3	9.4	4	3.5	11.0	4.5	4	14.1	9	9	63.6	6	7	33.0	8	8	50.2	13	12	122.5	21	21	346.2
4	3	9.4	4	3.5	11.0	4.5	4	14.1	9	9	63.6	6	7	33.0	8	8	50.2	13	12	122.5	21	21	346.2
4	3	9.4	4	3.5	11.0	4.5	4	14.1	9	9	63.6	6	7	33.0	9	8.5	60.1	13	12	122.5	21	21	346.2
3	3	7.1	4	3.5	11.0	4.5	4	14.1	9	9	63.6	7.5	8	47.1	9	8.5	60.1	13	12	122.5	21	21	346.2
3	3	7.1	4	3.5	11.0	4.5	4	14.1	9	9	63.6	7.5	8	47.1	9	8.5	60.1	13	12	122.5	21	21	346.2
4.5	4.5	15.9	4	3.5	11.0	4.5	4	14.1	9	9	63.6	7.5	8	47.1	9	8.5	60.1	13	12	122.5	21	21	346.2
4.5	4.5	15.9	4	3.5	11.0	4.5	4	14.1	9	9	63.6	7.5	8	47.1	9	8.5	60.1	13	12	122.5	21	21	346.2
4.5	4.5	15.9	4	3.5	11.0	4.5	4	14.1	9	9	63.6	7.5	8	47.1	9	8.5	60.1	14	13.5	148.4	21	21	346.2
4.5	4.5	15.9	4	3.5	11.0	4.5	4	14.1	9	9	63.6	7.5	8	47.1	9	8.5	60.1	14	13.5	148.4	21	21	346.2
4.5	4.5	15.9	4	3.5	11.0	4.5	4	14.1	9	9	63.6	7.5	8	47.1	9	8.5	60.1	14	13.5	148.4	21	21	346.2
4.5	4.5	15.9	6	6	28.3	4.5	4	14.1	9	9	63.6	7.5	8	47.1	9	8.5	60.1	14	13.5	148.4	21	21	346.2
4.5	4.5	15.9	6	6	28.3	2.5	3	5.9	9	9	63.6	7.5	8	47.1	9	8.5	60.1	14	13.5	148.4	21	21	346.2
4.5	4.5	15.9	6	6	28.3	2.5	3	5.9	10.5	2	16.5	7.5	8	47.1	9	8.5	60.1	14	13.5	148.4	21	21	346.2
4.5	4	14.1	6	6	28.3	2.5	3	5.9	10.5	2	16.5	7.5	8	47.1	9	8.5	60.1	14	13.5	148.4	20	21	329.7
4.5	4.5	15.9	6	6	28.3	2.5	3	5.9	10.5	2	16.5	7.5	8	47.1	9	8.5	60.1	14	13.5	148.4	20	21	329.7

4.5	4.5	15.9	6	6	28.3	2.5	3	5.9	10.5	2	16.5	7.5	8	47.1	9	7.5	53.0	14	13.5	148.4	20	21	329.7
4	4.5	14.1	8	7.5	47.1	2.5	3	5.9	10.5	2	16.5	7.5	8	47.1	8.5	7.5	50.0	14	13.5	148.4	20	21	329.7
4.5	3.5	12.4	8	7.5	47.1	2.5	3	5.9	10.5	2	16.5	7.5	8	47.1	8.5	7.5	50.0	14	13.5	148.4	20	21	329.7
4.5	4.5	15.9	8	7.5	47.1	2.5	3	5.9	10.5	2	16.5	7.5	8	47.1	8.5	7.5	50.0	14	13.5	148.4	20	21	329.7
4.5	4.5	15.9	8	7.5	47.1	2.5	3	5.9	10.5	2	16.5	7.5	7	41.2	8.5	7.5	50.0	14	13.5	148.4	20	21	329.7
4.5	4.5	15.9	8	7.5	47.1	2.5	3	5.9	10.5	2	16.5	7.5	7	41.2	8.5	7.5	50.0	14	13.5	148.4	20	21	329.7
4.5	4.5	15.9	8	7.5	47.1	2.5	2.5	4.9	10.5	2	16.5	7.5	7	41.2	8.5	7.5	50.0	14	13.5	148.4	2	14.5	22.8
3.5	3	8.2	8	7.5	47.1	2.5	2.5	4.9	10.5	2	16.5	7.5	7	41.2	8.5	7.5	50.0	14	13.5	148.4	2	14.5	22.8
3.5	3.5	9.6	8	7.5	47.1	2.5	2.5	4.9	10.5	2	16.5	7.5	7	41.2	8.5	7.5	50.0	20.5	20	321.9	2	14.5	22.8
3.5	3.5	9.6	8	7.5	47.1	2.5	2.5	4.9	11	12.5	107.9	7.5	7	41.2	8.5	7.5	50.0	20.5	20	321.9	2	14.5	22.8
3.5	3.5	9.6	8	7.5	47.1	2.5	2.5	4.9	11	12.5	107.9	5	11.5	45.1	14	13	142.9	20.5	20	321.9	15	14.5	170.7
4.5	3.5	12.4	8	7.5	47.1	2.5	2.5	4.9	11	12.5	107.9	11	11.5	99.3	14	13	142.9	20.5	20	321.9	15	14.5	170.7
4.5	3.5	12.4	8	7.5	47.1	2.5	2.5	4.9	11	12.5	107.9	11	11.5	99.3	14	13	142.9	20.5	20	321.9	15	14.5	170.7
4.5	3.5	12.4	8	7.5	47.1	2.5	2.5	4.9	11	12.5	107.9	11	11.5	99.3	14	13	142.9	20.5	20	321.9	15	14.5	170.7
4.5	3.5	12.4	8	7.5	47.1	10	9	70.7	11	12.5	107.9	11	11.5	99.3	14	13	142.9	20.5	20	321.9	15	14.5	170.7
4	3.5	11.0	8	7.5	47.1	10	9	70.7	11	12.5	107.9	11	11.5	99.3	14	13	142.9	20.5	20	321.9	14.5	14.5	165.0
4	3.5	11.0	8	7.5	47.1	10	9	70.7	11	12.5	107.9	11	11.5	99.3	14	13	142.9	20.5	20	321.9	14.5	14.5	165.0
4	3.5	11.0	8	7.5	47.1	10	9	70.7	11	12.5	107.9	11	11.5	99.3	14	13	142.9	20.5	20	321.9	14.5	14.5	165.0
4	3.5	11.0	8	7.5	47.1	10	9	70.7	9	9	63.6	16	19	238.6	14	13	142.9	20.5	20	321.9	14.5	14.5	165.0
4	3.5	11.0	7	6.5	35.7	10	9	70.7	9	9	63.6	10.5	10	82.4	14	13	142.9	20.5	20	321.9	4.1	14.5	46.7
4	3.5	11.0	7	6.5	35.7	10	9	70.7	9	9	63.6	10.5	10	82.4	14	13	142.9	20.5	20	321.9	4.1	14.5	46.7
4	3.5	11.0	7	6.5	35.7	10	9	70.7	9	9	63.6	10.5	10	82.4	14	13	142.9	15.5	15	182.5	4.1	14.5	46.7
4	3.5	11.0	7	6.5	35.7	10	9	70.7	9	9	63.6	10.5	10	82.4	15.5	15	182.5	15.5	15	182.5	4.1	14.5	46.7
4	3.5	11.0	7	6.5	35.7	10	9	70.7	9	9	63.6	10.5	10	82.4	15.5	15	182.5	15.5	15	182.5	4.1	14.5	46.7
4	3	9.4	7	6.5	35.7	10	9	70.7	9	9	63.6	10.5	10	82.4	15.5	15	182.5	15.5	15	182.5	4.1	14.5	46.7
3	3	7.1	7	6.5	35.7	10	9	70.7	9	9	63.6	10.5	10	82.4	15.5	15	182.5	15.5	15	182.5	4.1	14.5	46.7
3	3	7.1	6.5	6.5	33.2	10	9	70.7	10	10	78.5	10.5	10	82.4	15.5	15	182.5	15.5	15	182.5	4.1	14.5	46.7
3	3	7.1	6.5	6.5	33.2	9	8.5	60.1	10	10	78.5	10.5	10	82.4	15.5	15	182.5	15.5	15	182.5	4.1	14.5	46.7
4.5	4.5	15.9	6.5	6.5	33.2	9	8.5	60.1	10	10	78.5	10.5	10	82.4	15.5	15	182.5	15.5	15	182.5	4.1	14.5	46.7
4.5	4.5	15.9	6.5	6	30.6	9	8.5	60.1	10	10	78.5	10.5	10	82.4	15.5	15	182.5	15.5	15	182.5	4.1	14.5	46.7

Table I. Size of *C. parapsilosis* ATCC 22019 cells treated with different concentrations of the C1 compound after 48 h of culture. Results from this table were used for preparing Fig 4D and 4F in the main body of the paper.

***Candida parapsilosis* 48h**																											
**Control**				**C1 0.5 µg/ml**				**C1 1 µg/ml**				**C1 2 µg/ml**				**C1 4 µg/ml**				**C1 8 µg/ml**				**C1 16 µg/ml**			
A [µm]	B [µm]	Cell size [µm^2]	A/B	A [µm]	B [µm]	Cell size [µm^2]	A/B	A [µm]	B [µm]	Cell size [µm^2]	A/B	A [µm]	B [µm]	Cell size [µm^2]	A/B	A [µm]	B [µm]	Cell size [µm^2]	A/B	A [µm]	B [µm]	Cell size [µm^2]	A/B	A [µm]	B [µm]	Cell size [µm^2]	A/B
17.5	4.5	61.8	3.9	20	5	78.5	4.0	10	7.5	58.9	1.3	11.5	11.5	103.8	1.0	7.5	5	29.4	1.5	5	5	19.6	1.0	10	10	78.5	1.0
17.5	4.5	61.8	3.9	20	5	78.5	4.0	10	7.5	58.9	1.3	11.5	11.5	103.8	1.0	7.5	5	29.4	1.5	5	5	19.6	1.0	10	10	78.5	1.0
17.5	4.5	61.8	3.9	20	5	78.5	4.0	10	7.5	58.9	1.3	11.5	11.5	103.8	1.0	7.5	5	29.4	1.5	5	5	19.6	1.0	10	10	78.5	1.0
17.5	4.5	61.8	3.9	20	5	78.5	4.0	10	7.5	58.9	1.3	11.5	11.5	103.8	1.0	7.5	5	29.4	1.5	5	5	19.6	1.0	10	10	78.5	1.0
17.5	4.5	61.8	3.9	20	5	78.5	4.0	10	7.5	58.9	1.3	11.5	11.5	103.8	1.0	7.5	5	29.4	1.5	5	5	19.6	1.0	10	10	78.5	1.0
17.5	4.5	61.8	3.9	20	5	78.5	4.0	10	7.5	58.9	1.3	11.5	11.5	103.8	1.0	7.5	5	29.4	1.5	5	5	19.6	1.0	10	10	78.5	1.0
17.5	4.5	61.8	3.9	20	5	78.5	4.0	10	7.5	58.9	1.3	11.5	11.5	103.8	1.0	7.5	5	29.4	1.5	5	5	19.6	1.0	10	10	78.5	1.0
17.5	4.5	61.8	3.9	20	5	78.5	4.0	10	7.5	58.9	1.3	11.5	11.5	103.8	1.0	7.5	5	29.4	1.5	5	5	19.6	1.0	10	10	78.5	1.0
17.5	4.5	61.8	3.9	20	5	78.5	4.0	10	7.5	58.9	1.3	11.5	11.5	103.8	1.0	7.5	5	29.4	1.5	5	5	19.6	1.0	10	10	78.5	1.0
17.5	4.5	61.8	3.9	20	5	78.5	4.0	10	7.5	58.9	1.3	11.5	11.5	103.8	1.0	7.5	5	29.4	1.5	5	5	19.6	1.0	10	10	78.5	1.0
17.5	4.5	61.8	3.9	20	5	78.5	4.0	10	7.5	58.9	1.3	11.5	11.5	103.8	1.0	7.5	5	29.4	1.5	5	5	19.6	1.0	10	10	78.5	1.0
17.5	4.5	61.8	3.9	20	5	78.5	4.0	10	7.5	58.9	1.3	11.5	11.5	103.8	1.0	7.5	5	29.4	1.5	5	5	19.6	1.0	10	10	78.5	1.0
17.5	4.5	61.8	3.9	20	5	78.5	4.0	10	7.5	58.9	1.3	11.5	11.5	103.8	1.0	7.5	5	29.4	1.5	5	5	19.6	1.0	10	10	78.5	1.0
17.5	4.5	61.8	3.9	20	5	78.5	4.0	10	7.5	58.9	1.3	11.5	11.5	103.8	1.0	7.5	5	29.4	1.5	5	5	19.6	1.0	10	10	78.5	1.0
17.5	4.5	61.8	3.9	20	5	78.5	4.0	10	7.5	58.9	1.3	11.5	11.5	103.8	1.0	7.5	5	29.4	1.5	5	5	19.6	1.0	10	10	78.5	1.0
17.5	4.5	61.8	3.9	20	5	78.5	4.0	10	7.5	58.9	1.3	11.5	11.5	103.8	1.0	11.5	10	90.3	1.2	5	5	19.6	1.0	10	10	78.5	1.0
17.5	4.5	61.8	3.9	20	5	78.5	4.0	10	7.5	58.9	1.3	11.5	11.5	103.8	1.0	11.5	10	90.3	1.2	5	5	19.6	1.0	10	10	78.5	1.0
17.5	4.5	61.8	3.9	20	5	78.5	4.0	10	7.5	58.9	1.3	11.5	11.5	103.8	1.0	11.5	10	90.3	1.2	5	5	19.6	1.0	10	10	78.5	1.0
17.5	4.5	61.8	3.9	20	5	78.5	4.0	17.5	13	178.6	1.3	11.5	11.5	103.8	1.0	11.5	10	90.3	1.2	5	5	19.6	1.0	10	10	78.5	1.0
17.5	4.5	61.8	3.9	20	5	78.5	4.0	17.5	13	178.6	1.3	11.5	11.5	103.8	1.0	11.5	10	90.3	1.2	5	5	19.6	1.0	10	10	78.5	1.0
17.5	4.5	61.8	3.9	20	5	78.5	4.0	17.5	13	178.6	1.3	11.5	11.5	103.8	1.0	11.5	10	90.3	1.2	5	5	19.6	1.0	10	10	78.5	1.0
17.5	4.5	61.8	3.9	20	5	78.5	4.0	17.5	13	178.6	1.3	11.5	11.5	103.8	1.0	11.5	10	90.3	1.2	5	5	19.6	1.0	10	10	78.5	1.0
17.5	4.5	61.8	3.9	20	5	78.5	4.0	17.5	13	178.6	1.3	11.5	11.5	103.8	1.0	11.5	10	90.3	1.2	5	5	19.6	1.0	10	10	78.5	1.0
17.5	4.5	61.8	3.9	20	5	78.5	4.0	17.5	13	178.6	1.3	11.5	11.5	103.8	1.0	11.5	10	90.3	1.2	5	5	19.6	1.0	10	10	78.5	1.0
17.5	4.5	61.8	3.9	20	5	78.5	4.0	17.5	13	178.6	1.3	11.5	11.5	103.8	1.0	11.5	10	90.3	1.2	5	5	19.6	1.0	10	10	78.5	1.0

17.5	4.5	61.8	3.9	20	5	78.5	4.0	17.5	13	178.6	1.3	10	10	78.5	1.0	11.5	10	90.3	1.2	5	5	19.6	1.0	10	10	78.5	1.0
17.5	4.5	61.8	3.9	20	5	78.5	4.0	17.5	13	178.6	1.3	10	10	78.5	1.0	11.5	10	90.3	1.2	20	20	314.0	1.0	9	8	56.5	1.1
17.5	4.5	61.8	3.9	30	4	94.2	7.5	17.5	13	178.6	1.3	10	10	78.5	1.0	11.5	10	90.3	1.2	20	20	314.0	1.0	9	8	56.5	1.1
20	5	78.5	4.0	30	4	94.2	7.5	17.5	13	178.6	1.3	10	10	78.5	1.0	11.5	10	90.3	1.2	20	20	314.0	1.0	9	8	56.5	1.1
20	5	78.5	4.0	30	4	94.2	7.5	17.5	13	178.6	1.3	10	10	78.5	1.0	11.5	10	90.3	1.2	20	20	314.0	1.0	9	8	56.5	1.1
20	5	78.5	4.0	30	4	94.2	7.5	17.5	13	178.6	1.3	10	10	78.5	1.0	11.5	10	90.3	1.2	20	20	314.0	1.0	9	8	56.5	1.1
20	5	78.5	4.0	30	4	94.2	7.5	17.5	13	178.6	1.3	10	10	78.5	1.0	11.5	10	90.3	1.2	20	20	314.0	1.0	9	8	56.5	1.1
20	5	78.5	4.0	30	4	94.2	7.5	17.5	13	178.6	1.3	10	10	78.5	1.0	11.5	10	90.3	1.2	20	20	314.0	1.0	9	8	56.5	1.1
20	5	78.5	4.0	30	4	94.2	7.5	17.5	13	178.6	1.3	10	10	78.5	1.0	11.5	10	90.3	1.2	20	20	314.0	1.0	9	8	56.5	1.1
20	5	78.5	4.0	30	4	94.2	7.5	17.5	13	178.6	1.3	10	10	78.5	1.0	16	15.5	194.7	1.0	20	20	314.0	1.0	9	8	56.5	1.1
20	5	78.5	4.0	30	4	94.2	7.5	17.5	13	178.6	1.3	10	10	78.5	1.0	16	15.5	194.7	1.0	20	20	314.0	1.0	9	8	56.5	1.1
20	5	78.5	4.0	30	4	94.2	7.5	17.5	13	178.6	1.3	10	10	78.5	1.0	16	15.5	194.7	1.0	20	20	314.0	1.0	9	8	56.5	1.1
20	5	78.5	4.0	30	4	94.2	7.5	17.5	13	178.6	1.3	10	10	78.5	1.0	16	15.5	194.7	1.0	20	20	314.0	1.0	9	8	56.5	1.1
20	5	78.5	4.0	30	4	94.2	7.5	17.5	13	178.6	1.3	10	10	78.5	1.0	16	15.5	194.7	1.0	20	20	314.0	1.0	9	8	56.5	1.1
20	5	78.5	4.0	30	4	94.2	7.5	17.5	13	178.6	1.3	10	10	78.5	1.0	16	15.5	194.7	1.0	20	20	314.0	1.0	9	8	56.5	1.1
20	5	78.5	4.0	30	4	94.2	7.5	17.5	13	178.6	1.3	10	10	78.5	1.0	16	15.5	194.7	1.0	20	20	314.0	1.0	9	8	56.5	1.1
20	5	78.5	4.0	30	4	94.2	7.5	17.5	13	178.6	1.3	10	10	78.5	1.0	16	15.5	194.7	1.0	20	20	314.0	1.0	9	8	56.5	1.1
20	5	78.5	4.0	30	4	94.2	7.5	12.5	7.5	73.6	1.7	10	10	78.5	1.0	16	15.5	194.7	1.0	20	20	314.0	1.0	9	8	56.5	1.1
20	5	78.5	4.0	30	4	94.2	7.5	12.5	7.5	73.6	1.7	10	10	78.5	1.0	16	15.5	194.7	1.0	20	20	314.0	1.0	9	8	56.5	1.1
20	5	78.5	4.0	30	4	94.2	7.5	12.5	7.5	73.6	1.7	10	10	78.5	1.0	16	15.5	194.7	1.0	20	20	314.0	1.0	9	8	56.5	1.1
20	5	78.5	4.0	30	4	94.2	7.5	12.5	7.5	73.6	1.7	10	12.5	98.1	0.8	16	15.5	194.7	1.0	20	20	314.0	1.0	9	8	56.5	1.1
15	7.5	88.3	2.0	12.5	4	39.3	3.1	12.5	7.5	73.6	1.7	10	12.5	98.1	0.8	16	15.5	194.7	1.0	20	20	314.0	1.0	9	8	56.5	1.1
15	7.5	88.3	2.0	12.5	4	39.3	3.1	12.5	7.5	73.6	1.7	10	12.5	98.1	0.8	16	15.5	194.7	1.0	20	20	314.0	1.0	9	8	56.5	1.1
15	7.5	88.3	2.0	12.5	4	39.3	3.1	12.5	7.5	73.6	1.7	10	12.5	98.1	0.8	16	15.5	194.7	1.0	20	20	314.0	1.0	9	8	56.5	1.1
15	7.5	88.3	2.0	12.5	4	39.3	3.1	12.5	7.5	73.6	1.7	10	12.5	98.1	0.8	16	15.5	194.7	1.0	20	20	314.0	1.0	9	8	56.5	1.1
15	7.5	88.3	2.0	12.5	4	39.3	3.1	12.5	7.5	73.6	1.7	10	12.5	98.1	0.8	16	15.5	194.7	1.0	20	20	314.0	1.0	9	8	56.5	1.1
15	7.5	88.3	2.0	12.5	4	39.3	3.1	12.5	7.5	73.6	1.7	10	12.5	98.1	0.8	16	15.5	194.7	1.0	20	20	314.0	1.0	9	8	56.5	1.1
15	7.5	88.3	2.0	12.5	4	39.3	3.1	12.5	7.5	73.6	1.7	10	12.5	98.1	0.8	16	15.5	194.7	1.0	20	20	314.0	1.0	9	8	56.5	1.1
15	7.5	88.3	2.0	12.5	4	39.3	3.1	12.5	7.5	73.6	1.7	10	12.5	98.1	0.8	17.5	17	233.5	1.0	20	20	314.0	1.0	9	8	56.5	1.1
15	7.5	88.3	2.0	12.5	4	39.3	3.1	12.5	7.5	73.6	1.7	10	12.5	98.1	0.8	17.5	17	233.5	1.0	20	20	314.0	1.0	9	8	56.5	1.1
15	7.5	88.3	2.0	12.5	4	39.3	3.1	12.5	7.5	73.6	1.7	10	12.5	98.1	0.8	17.5	17	233.5	1.0	20	20	314.0	1.0	15	16	188.4	0.9
15	7.5	88.3	2.0	12.5	4	39.3	3.1	12.5	7.5	73.6	1.7	10	12.5	98.1	0.8	17.5	17	233.5	1.0	20	20	314.0	1.0	15	16	188.4	0.9
15	7.5	88.3	2.0	12.5	4	39.3	3.1	12.5	7.5	73.6	1.7	10	12.5	98.1	0.8	17.5	17	233.5	1.0	20	20	314.0	1.0	15	16	188.4	0.9
15	7.5	88.3	2.0	12.5	4	39.3	3.1	12.5	7.5	73.6	1.7	10	12.5	98.1	0.8	17.5	17	233.5	1.0	20	20	314.0	1.0	15	16	188.4	0.9
15	7.5	88.3	2.0	12.5	4	39.3	3.1	12.5	7.5	73.6	1.7	10	12.5	98.1	0.8	17.5	17	233.5	1.0	20	20	314.0	1.0	15	16	188.4	0.9
15	7.5	88.3	2.0	12.5	4	39.3	3.1	12.5	7.5	73.6	1.7	10	12.5	98.1	0.8	17.5	17	233.5	1.0	20	20	314.0	1.0	15	16	188.4	0.9

15	7.5	88.3	2.0	12.5	4	39.3	3.1	12.5	7.5	73.6	1.7	10	12.5	98.1	0.8	17.5	17	233.5	1.0	20	20	314.0	1.0	15	16	188.4	0.9
10	5	39.3	2.0	12.5	4	39.3	3.1	12.5	7.5	73.6	1.7	10	12.5	98.1	0.8	17.5	17	233.5	1.0	20	20	314.0	1.0	15	16	188.4	0.9
10	5	39.3	2.0	12.5	4	39.3	3.1	12.5	7.5	73.6	1.7	7.5	7	41.2	1.1	17.5	17	233.5	1.0	20	20	314.0	1.0	15	16	188.4	0.9
10	5	39.3	2.0	12.5	4	39.3	3.1	12.5	7.5	73.6	1.7	7.5	7	41.2	1.1	17.5	17	233.5	1.0	17.5	17.5	240.4	1.0	15	16	188.4	0.9
10	5	39.3	2.0	25	3.5	68.7	7.1	7.5	4.5	26.5	1.7	7.5	7	41.2	1.1	17.5	17	233.5	1.0	17.5	17.5	240.4	1.0	15	16	188.4	0.9
10	5	39.3	2.0	25	3.5	68.7	7.1	7.5	4.5	26.5	1.7	7.5	7	41.2	1.1	17.5	17	233.5	1.0	17.5	17.5	240.4	1.0	15	16	188.4	0.9
10	5	39.3	2.0	25	3.5	68.7	7.1	7.5	4.5	26.5	1.7	7.5	7	41.2	1.1	17.5	17	233.5	1.0	17.5	17.5	240.4	1.0	15	16	188.4	0.9
10	5	39.3	2.0	25	3.5	68.7	7.1	7.5	4.5	26.5	1.7	7.5	7	41.2	1.1	17.5	17	233.5	1.0	17.5	17.5	240.4	1.0	15	16	188.4	0.9
10	5	39.3	2.0	25	3.5	68.7	7.1	7.5	4.5	26.5	1.7	7.5	7	41.2	1.1	17.5	17	233.5	1.0	17.5	17.5	240.4	1.0	15	16	188.4	0.9
10	5	39.3	2.0	25	3.5	68.7	7.1	7.5	4.5	26.5	1.7	7.5	7	41.2	1.1	17.5	17	233.5	1.0	17.5	17.5	240.4	1.0	15	16	188.4	0.9
10	5	39.3	2.0	25	3.5	68.7	7.1	7.5	4.5	26.5	1.7	7.5	7	41.2	1.1	17.5	17	233.5	1.0	17.5	17.5	240.4	1.0	15	16	188.4	0.9
10	5	39.3	2.0	25	3.5	68.7	7.1	7.5	4.5	26.5	1.7	7.5	7	41.2	1.1	17.5	17	233.5	1.0	17.5	17.5	240.4	1.0	15	16	188.4	0.9
10	5	39.3	2.0	25	3.5	68.7	7.1	7.5	4.5	26.5	1.7	7.5	7	41.2	1.1	17.5	17	233.5	1.0	17.5	17.5	240.4	1.0	15	16	188.4	0.9
10	5	39.3	2.0	25	3.5	68.7	7.1	7.5	4.5	26.5	1.7	7.5	7	41.2	1.1	17.5	17	233.5	1.0	17.5	17.5	240.4	1.0	15	16	188.4	0.9
10	5	39.3	2.0	25	3.5	68.7	7.1	7.5	4.5	26.5	1.7	7.5	7	41.2	1.1	17.5	17	233.5	1.0	17.5	17.5	240.4	1.0	15	16	188.4	0.9
10	5	39.3	2.0	25	3.5	68.7	7.1	7.5	4.5	26.5	1.7	7.5	7	41.2	1.1	17.5	17	233.5	1.0	17.5	17.5	240.4	1.0	15	16	188.4	0.9
10	5	39.3	2.0	25	3.5	68.7	7.1	7.5	4.5	26.5	1.7	7.5	7	41.2	1.1	14	13.5	148.4	1.0	17.5	17.5	240.4	1.0	15	16	188.4	0.9
10	5	39.3	2.0	25	3.5	68.7	7.1	7.5	4.5	26.5	1.7	7.5	7	41.2	1.1	14	13.5	148.4	1.0	17.5	17.5	240.4	1.0	15	16	188.4	0.9
10	5	39.3	2.0	22.5	5	88.3	4.5	7.5	4.5	26.5	1.7	7.5	7	41.2	1.1	14	13.5	148.4	1.0	17.5	17.5	240.4	1.0	15	16	188.4	0.9
10	5	39.3	2.0	22.5	5	88.3	4.5	7.5	4.5	26.5	1.7	7.5	7	41.2	1.1	14	13.5	148.4	1.0	17.5	17.5	240.4	1.0	20	20	314.0	1.0
10	5	39.3	2.0	22.5	5	88.3	4.5	7.5	4.5	26.5	1.7	7.5	7	41.2	1.1	14	13.5	148.4	1.0	17.5	17.5	240.4	1.0	20	20	314.0	1.0
15	6	70.7	2.5	22.5	5	88.3	4.5	7.5	4.5	26.5	1.7	7.5	7	41.2	1.1	14	13.5	148.4	1.0	17.5	17.5	240.4	1.0	20	20	314.0	1.0
15	6	70.7	2.5	22.5	5	88.3	4.5	15	10	117.8	1.5	7.5	7	41.2	1.1	14	13.5	148.4	1.0	17.5	17.5	240.4	1.0	20	20	314.0	1.0
15	6	70.7	2.5	22.5	5	88.3	4.5	15	10	117.8	1.5	7.5	7	41.2	1.1	14	13.5	148.4	1.0	17.5	17.5	240.4	1.0	20	20	314.0	1.0
15	6	70.7	2.5	22.5	5	88.3	4.5	15	10	117.8	1.5	7.5	7	41.2	1.1	14	13.5	148.4	1.0	17.5	17.5	240.4	1.0	20	20	314.0	1.0
15	6	70.7	2.5	22.5	5	88.3	4.5	15	10	117.8	1.5	7.5	7	41.2	1.1	14	13.5	148.4	1.0	17.5	17.5	240.4	1.0	20	20	314.0	1.0
15	6	70.7	2.5	22.5	5	88.3	4.5	15	10	117.8	1.5	9.5	6.5	48.5	1.5	14	13.5	148.4	1.0	17.5	17.5	240.4	1.0	20	20	314.0	1.0
15	6	70.7	2.5	22.5	5	88.3	4.5	15	10	117.8	1.5	9.5	6.5	48.5	1.5	14	13.5	148.4	1.0	17.5	17.5	240.4	1.0	20	20	314.0	1.0
15	6	70.7	2.5	22.5	5	88.3	4.5	15	10	117.8	1.5	9.5	6.5	48.5	1.5	14	13.5	148.4	1.0	17.5	17.5	240.4	1.0	20	20	314.0	1.0
15	6	70.7	2.5	22.5	5	88.3	4.5	15	10	117.8	1.5	9.5	6.5	48.5	1.5	14	13.5	148.4	1.0	17.5	17.5	240.4	1.0	20	20	314.0	1.0
15	6	70.7	2.5	22.5	5	88.3	4.5	15	10	117.8	1.5	9.5	6.5	48.5	1.5	14	13.5	148.4	1.0	17.5	17.5	240.4	1.0	20	20	314.0	1.0
15	6	70.7	2.5	22.5	5	88.3	4.5	15	10	117.8	1.5	9.5	6.5	48.5	1.5	14	13.5	148.4	1.0	17.5	17.5	240.4	1.0	20	20	314.0	1.0
15	6	70.7	2.5	22.5	5	88.3	4.5	15	10	117.8	1.5	9.5	6.5	48.5	1.5	14	13.5	148.4	1.0	17.5	17.5	240.4	1.0	20	20	314.0	1.0
15	6	70.7	2.5	15	2.5	29.4	6.0	15	10	117.8	1.5	9.5	6.5	48.5	1.5	14	13.5	148.4	1.0	17.5	17.5	240.4	1.0	20	20	314.0	1.0
15	6	70.7	2.5	15	2.5	29.4	6.0	15	10	117.8	1.5	9.5	6.5	48.5	1.5	14	13.5	148.4	1.0	17.5	17.5	240.4	1.0	20	20	314.0	1.0
15	6	70.7	2.5	15	2.5	29.4	6.0	15	10	117.8	1.5	9.5	6.5	48.5	1.5	14	13.5	148.4	1.0	17.5	17.5	240.4	1.0	20	20	314.0	1.0

15	6	70.7	2.5	15	2.5	29.4	6.0	15	10	117.8	1.5	9.5	6.5	48.5	1.5	14	13.5	148.4	1.0	17.5	17.5	240.4	1.0	20	20	314.0	1.0
15	6	70.7	2.5	15	2.5	29.4	6.0	15	10	117.8	1.5	9.5	6.5	48.5	1.5	14	13.5	148.4	1.0	17.5	17.5	240.4	1.0	20	20	314.0	1.0
15	6	70.7	2.5	15	2.5	29.4	6.0	15	10	117.8	1.5	9.5	6.5	48.5	1.5	14	13.5	148.4	1.0	17.5	17.5	240.4	1.0	20	20	314.0	1.0
15	6	70.7	2.5	15	2.5	29.4	6.0	15	10	117.8	1.5	9.5	6.5	48.5	1.5	14	13.5	148.4	1.0	17.5	17.5	240.4	1.0	20	20	314.0	1.0
15	6	70.7	2.5	15	2.5	29.4	6.0	15	10	117.8	1.5	9.5	6.5	48.5	1.5	14	13.5	148.4	1.0	17.5	17.5	240.4	1.0	20	20	314.0	1.0
15	6	70.7	2.5	15	2.5	29.4	6.0	15	10	117.8	1.5	9.5	6.5	48.5	1.5	14	13.5	148.4	1.0	17.5	17.5	240.4	1.0	20	20	314.0	1.0
10	5	39.3	2.0	15	2.5	29.4	6.0	15	10	117.8	1.5	9.5	6.5	48.5	1.5	20	18.5	290.5	1.1	12.5	13	127.6	1.0	20	20	314.0	1.0
10	5	39.3	2.0	15	2.5	29.4	6.0	15	10	117.8	1.5	9.5	6.5	48.5	1.5	20	18.5	290.5	1.1	12.5	13	127.6	1.0	20	20	314.0	1.0
10	5	39.3	2.0	15	2.5	29.4	6.0	15	10	117.8	1.5	9.5	6.5	48.5	1.5	20	18.5	290.5	1.1	12.5	13	127.6	1.0	12.5	12.5	122.7	1.0
10	5	39.3	2.0	15	2.5	29.4	6.0	14	10	109.9	1.4	9.5	6.5	48.5	1.5	20	18.5	290.5	1.1	12.5	13	127.6	1.0	12.5	12.5	122.7	1.0
10	5	39.3	2.0	15	2.5	29.4	6.0	14	10	109.9	1.4	9.5	6.5	48.5	1.5	20	18.5	290.5	1.1	12.5	13	127.6	1.0	12.5	12.5	122.7	1.0
10	5	39.3	2.0	15	2.5	29.4	6.0	14	10	109.9	1.4	9.5	6.5	48.5	1.5	20	18.5	290.5	1.1	12.5	13	127.6	1.0	12.5	12.5	122.7	1.0
10	5	39.3	2.0	15	2.5	29.4	6.0	14	10	109.9	1.4	9.5	6.5	48.5	1.5	20	18.5	290.5	1.1	12.5	13	127.6	1.0	12.5	12.5	122.7	1.0
10	5	39.3	2.0	15	2.5	29.4	6.0	14	10	109.9	1.4	9.5	6.5	48.5	1.5	20	18.5	290.5	1.1	12.5	13	127.6	1.0	12.5	12.5	122.7	1.0
10	5	39.3	2.0	15	2.5	29.4	6.0	14	10	109.9	1.4	15	12.5	147.2	1.2	20	18.5	290.5	1.1	12.5	13	127.6	1.0	12.5	12.5	122.7	1.0
10	5	39.3	2.0	15	2.5	29.4	6.0	14	10	109.9	1.4	15	12.5	147.2	1.2	20	18.5	290.5	1.1	12.5	13	127.6	1.0	12.5	12.5	122.7	1.0
10	5	39.3	2.0	15	2.5	29.4	6.0	14	10	109.9	1.4	15	12.5	147.2	1.2	20	18.5	290.5	1.1	12.5	13	127.6	1.0	12.5	12.5	122.7	1.0
10	5	39.3	2.0	15	2.5	29.4	6.0	14	10	109.9	1.4	15	12.5	147.2	1.2	20	18.5	290.5	1.1	12.5	13	127.6	1.0	12.5	12.5	122.7	1.0
10	5	39.3	2.0	15	2.5	29.4	6.0	14	10	109.9	1.4	15	12.5	147.2	1.2	20	18.5	290.5	1.1	12.5	13	127.6	1.0	12.5	12.5	122.7	1.0
10	5	39.3	2.0	15	2.5	29.4	6.0	14	10	109.9	1.4	15	12.5	147.2	1.2	20	18.5	290.5	1.1	12.5	13	127.6	1.0	12.5	12.5	122.7	1.0
10	5	39.3	2.0	15	2.5	29.4	6.0	14	10	109.9	1.4	15	12.5	147.2	1.2	20	18.5	290.5	1.1	12.5	13	127.6	1.0	12.5	12.5	122.7	1.0
10	5	39.3	2.0	15	2.5	29.4	6.0	14	10	109.9	1.4	15	12.5	147.2	1.2	20	18.5	290.5	1.1	12.5	13	127.6	1.0	12.5	12.5	122.7	1.0
10	5	39.3	2.0	15	2.5	29.4	6.0	14	10	109.9	1.4	15	12.5	147.2	1.2	20	18.5	290.5	1.1	12.5	13	127.6	1.0	12.5	12.5	122.7	1.0
10	5	39.3	2.0	15	2.5	29.4	6.0	14	10	109.9	1.4	15	12.5	147.2	1.2	20	18.5	290.5	1.1	12.5	13	127.6	1.0	12.5	12.5	122.7	1.0
10	5	39.3	2.0	15	2.5	29.4	6.0	14	10	109.9	1.4	15	12.5	147.2	1.2	20	18.5	290.5	1.1	12.5	13	127.6	1.0	12.5	12.5	122.7	1.0
10	5	39.3	2.0	20	3	47.1	6.7	14	10	109.9	1.4	15	12.5	147.2	1.2	20	18.5	290.5	1.1	12.5	13	127.6	1.0	12.5	12.5	122.7	1.0
10	5	39.3	2.0	20	3	47.1	6.7	14	10	109.9	1.4	15	12.5	147.2	1.2	20	18.5	290.5	1.1	12.5	13	127.6	1.0	12.5	12.5	122.7	1.0
10	5	39.3	2.0	20	3	47.1	6.7	14	10	109.9	1.4	15	12.5	147.2	1.2	22.5	22.5	397.4	1.0	12.5	13	127.6	1.0	12.5	12.5	122.7	1.0
10	5	39.3	2.0	20	3	47.1	6.7	14	10	109.9	1.4	15	12.5	147.2	1.2	22.5	22.5	397.4	1.0	12.5	13	127.6	1.0	12.5	12.5	122.7	1.0
12.5	10	98.1	1.3	20	3	47.1	6.7	14	10	109.9	1.4	15	12.5	147.2	1.2	22.5	22.5	397.4	1.0	15	15	176.6	1.0	12.5	12.5	122.7	1.0
12.5	10	98.1	1.3	20	3	47.1	6.7	14	10	109.9	1.4	15	12.5	147.2	1.2	22.5	22.5	397.4	1.0	15	15	176.6	1.0	12.5	12.5	122.7	1.0
12.5	10	98.1	1.3	20	3	47.1	6.7	14	10	109.9	1.4	15	12.5	147.2	1.2	22.5	22.5	397.4	1.0	15	15	176.6	1.0	12.5	12.5	122.7	1.0
12.5	10	98.1	1.3	20	3	47.1	6.7	14	10	109.9	1.4	15	12.5	147.2	1.2	22.5	22.5	397.4	1.0	15	15	176.6	1.0	12.5	12.5	122.7	1.0
12.5	10	98.1	1.3	20	3	47.1	6.7	14	10	109.9	1.4	15	12.5	147.2	1.2	22.5	22.5	397.4	1.0	15	15	176.6	1.0	12.5	12.5	122.7	1.0
12.5	10	98.1	1.3	20	3	47.1	6.7	14	10	109.9	1.4	15	12.5	147.2	1.2	22.5	22.5	397.4	1.0	15	15	176.6	1.0	12.5	12.5	122.7	1.0
12.5	10	98.1	1.3	20	3	47.1	6.7	7.5	7.5	44.2	1.0	15	12.5	147.2	1.2	22.5	22.5	397.4	1.0	15	15	176.6	1.0	12.5	12.5	122.7	1.0

12.5	10	98.1	1.3	20	3	47.1	6.7	7.5	7.5	44.2	1.0	15	12.5	147.2	1.2	22.5	22.5	397.4	1.0	15	15	176.6	1.0	12.5	12.5	122.7	1.0
12.5	10	98.1	1.3	20	3	47.1	6.7	7.5	7.5	44.2	1.0	15	12.5	147.2	1.2	22.5	22.5	397.4	1.0	15	15	176.6	1.0	12.5	12.5	122.7	1.0
12.5	10	98.1	1.3	20	3	47.1	6.7	7.5	7.5	44.2	1.0	15	12.5	147.2	1.2	22.5	22.5	397.4	1.0	15	15	176.6	1.0	12.5	12.5	122.7	1.0
12.5	10	98.1	1.3	20	3	47.1	6.7	7.5	7.5	44.2	1.0	15	12.5	147.2	1.2	22.5	22.5	397.4	1.0	15	15	176.6	1.0	16	15	188.4	1.1
12.5	10	98.1	1.3	20	3	47.1	6.7	7.5	7.5	44.2	1.0	15	12.5	147.2	1.2	22.5	22.5	397.4	1.0	15	15	176.6	1.0	16	15	188.4	1.1
12.5	10	98.1	1.3	20	3	47.1	6.7	7.5	7.5	44.2	1.0	14	10.5	115.4	1.3	22.5	22.5	397.4	1.0	15	15	176.6	1.0	16	15	188.4	1.1
12.5	10	98.1	1.3	20	3	47.1	6.7	7.5	7.5	44.2	1.0	14	10.5	115.4	1.3	22.5	22.5	397.4	1.0	15	15	176.6	1.0	16	15	188.4	1.1
12.5	10	98.1	1.3	20	3	47.1	6.7	7.5	7.5	44.2	1.0	14	10.5	115.4	1.3	22.5	22.5	397.4	1.0	15	15	176.6	1.0	16	15	188.4	1.1
12.5	10	98.1	1.3	20	3	47.1	6.7	7.5	7.5	44.2	1.0	14	10.5	115.4	1.3	22.5	22.5	397.4	1.0	15	15	176.6	1.0	16	15	188.4	1.1
12.5	10	98.1	1.3	20	3	47.1	6.7	7.5	7.5	44.2	1.0	14	10.5	115.4	1.3	22.5	22.5	397.4	1.0	15	15	176.6	1.0	16	15	188.4	1.1
12.5	10	98.1	1.3	20	3	47.1	6.7	7.5	7.5	44.2	1.0	14	10.5	115.4	1.3	22.5	22.5	397.4	1.0	15	15	176.6	1.0	16	15	188.4	1.1
12.5	10	98.1	1.3	20	3	47.1	6.7	7.5	7.5	44.2	1.0	14	10.5	115.4	1.3	22.5	22.5	397.4	1.0	15	15	176.6	1.0	16	15	188.4	1.1
15	10	117.8	1.5	20	3	47.1	6.7	7.5	7.5	44.2	1.0	14	10.5	115.4	1.3	22.5	22.5	397.4	1.0	15	15	176.6	1.0	16	15	188.4	1.1
15	10	117.8	1.5	20	3	47.1	6.7	7.5	7.5	44.2	1.0	14	10.5	115.4	1.3	22.5	22.5	397.4	1.0	15	17.5	206.1	0.9	16	15	188.4	1.1
15	10	117.8	1.5	20	3	47.1	6.7	7.5	7.5	44.2	1.0	14	10.5	115.4	1.3	22.5	22.5	397.4	1.0	15	17.5	206.1	0.9	16	15	188.4	1.1
15	10	117.8	1.5	20	3	47.1	6.7	7.5	7.5	44.2	1.0	14	10.5	115.4	1.3	10	9	70.7	1.1	15	17.5	206.1	0.9	16	15	188.4	1.1
15	10	117.8	1.5	20	3	47.1	6.7	7.5	7.5	44.2	1.0	14	10.5	115.4	1.3	10	9	70.7	1.1	15	17.5	206.1	0.9	16	15	188.4	1.1
15	10	117.8	1.5	20	3	47.1	6.7	7.5	7.5	44.2	1.0	14	10.5	115.4	1.3	10	9	70.7	1.1	15	17.5	206.1	0.9	16	15	188.4	1.1
15	10	117.8	1.5	20	3	47.1	6.7	7.5	7.5	44.2	1.0	14	10.5	115.4	1.3	10	9	70.7	1.1	15	17.5	206.1	0.9	16	15	188.4	1.1
15	10	117.8	1.5	20	3	47.1	6.7	7.5	7.5	44.2	1.0	13.5	14	148.4	1.0	10	9	70.7	1.1	15	17.5	206.1	0.9	16	15	188.4	1.1
15	10	117.8	1.5	30	4	94.2	7.5	9	6	42.4	1.5	13.5	14	148.4	1.0	10	9	70.7	1.1	15	17.5	206.1	0.9	16	15	188.4	1.1
15	10	117.8	1.5	30	4	94.2	7.5	9	6	42.4	1.5	13.5	14	148.4	1.0	10	9	70.7	1.1	15	17.5	206.1	0.9	16	15	188.4	1.1
15	10	117.8	1.5	30	4	94.2	7.5	9	6	42.4	1.5	13.5	14	148.4	1.0	10	9	70.7	1.1	15	17.5	206.1	0.9	16	15	188.4	1.1
15	10	117.8	1.5	30	4	94.2	7.5	9	6	42.4	1.5	13.5	14	148.4	1.0	10	9	70.7	1.1	15	17.5	206.1	0.9	16	15	188.4	1.1
15	10	117.8	1.5	30	4	94.2	7.5	9	6	42.4	1.5	13.5	14	148.4	1.0	10	9	70.7	1.1	15	17.5	206.1	0.9	16	15	188.4	1.1
15	10	117.8	1.5	30	4	94.2	7.5	9	6	42.4	1.5	13.5	14	148.4	1.0	10	9	70.7	1.1	15	17.5	206.1	0.9	16	15.5	194.7	1.0
15	10	117.8	1.5	30	4	94.2	7.5	9	6	42.4	1.5	13.5	14	148.4	1.0	10	9	70.7	1.1	15	17.5	206.1	0.9	16	15.5	194.7	1.0
15	10	117.8	1.5	30	4	94.2	7.5	9	6	42.4	1.5	13.5	14	148.4	1.0	10	9	70.7	1.1	15	17.5	206.1	0.9	16	15.5	194.7	1.0
15	10	117.8	1.5	30	4	94.2	7.5	9	6	42.4	1.5	13.5	14	148.4	1.0	10	9	70.7	1.1	15	17.5	206.1	0.9	16	15.5	194.7	1.0
15	10	117.8	1.5	30	4	94.2	7.5	9	6	42.4	1.5	13.5	14	148.4	1.0	10	9	70.7	1.1	15	17.5	206.1	0.9	16	15.5	194.7	1.0

15	10	117.8	1.5	30	4	94.2	7.5	9	6	42.4	1.5	13.5	14	148.4	1.0	10	9	70.7	1.1	15	17.5	206.1	0.9	16	15.5	194.7	1.0
15	10	117.8	1.5	30	4	94.2	7.5	9	6	42.4	1.5	13.5	14	148.4	1.0	10	9	70.7	1.1	15	17.5	206.1	0.9	16	15.5	194.7	1.0
15	10	117.8	1.5	30	4	94.2	7.5	9	6	42.4	1.5	13.5	14	148.4	1.0	10	9	70.7	1.1	15	17.5	206.1	0.9	16	15.5	194.7	1.0
15	10	117.8	1.5	30	4	94.2	7.5	9	6	42.4	1.5	13.5	14	148.4	1.0	10	9	70.7	1.1	15	17.5	206.1	0.9	5	2.5	9.8	2.0
15	9.5	111.9	1.6	30	4	94.2	7.5	9	6	42.4	1.5	13.5	2	21.2	6.8	10	9	70.7	1.1	15	17.5	206.1	0.9	5	2.5	9.8	2.0
15	9.5	111.9	1.6	30	4	94.2	7.5	9	6	42.4	1.5	12.5	2	19.6	6.3	10	9	70.7	1.1	15	17.5	206.1	0.9	5	2.5	9.8	2.0
15	9.5	111.9	1.6	30	4	94.2	7.5	9	6	42.4	1.5	12.5	2	19.6	6.3	10	9	70.7	1.1	15	17.5	206.1	0.9	5	2.5	9.8	2.0
15	9.5	111.9	1.6	30	4	94.2	7.5	9	6	42.4	1.5	12.5	2	19.6	6.3	10	9	70.7	1.1	15	17.5	206.1	0.9	5	2.5	9.8	2.0
15	9.5	111.9	1.6	30	4	94.2	7.5	9	6	42.4	1.5	12.5	2	19.6	6.3	10	9	70.7	1.1	15	17.5	206.1	0.9	5	2.5	9.8	2.0
15	9.5	111.9	1.6	30	4	94.2	7.5	9	6	42.4	1.5	12.5	2	19.6	6.3	10	9	70.7	1.1	15	17.5	206.1	0.9	5	2.5	9.8	2.0
15	9.5	111.9	1.6	30	4	94.2	7.5	9	6	42.4	1.5	12.5	2	19.6	6.3	10	9	70.7	1.1	10	2.5	19.6	4.0	5	2.5	9.8	2.0
15	9.5	111.9	1.6	30	4	94.2	7.5	9	6	42.4	1.5	12.5	2	19.6	6.3	15	15	176.6	1.0	10	2.5	19.6	4.0	5	2.5	9.8	2.0
15	9.5	111.9	1.6	30	4	94.2	7.5	9	6	42.4	1.5	12.5	2	19.6	6.3	15	15	176.6	1.0	10	2.5	19.6	4.0	5	2.5	9.8	2.0
15	9.5	111.9	1.6	30	4	94.2	7.5	9	6	42.4	1.5	12.5	2	19.6	6.3	15	15	176.6	1.0	10	2.5	19.6	4.0	5	2.5	9.8	2.0
15	9.5	111.9	1.6	30	4	94.2	7.5	9	6	42.4	1.5	12.5	2	19.6	6.3	15	15	176.6	1.0	10	2.5	19.6	4.0	5	2.5	9.8	2.0
15	9.5	111.9	1.6	30	4	94.2	7.5	9	6	42.4	1.5	12.5	2	19.6	6.3	15	15	176.6	1.0	10	2.5	19.6	4.0	5	2.5	9.8	2.0
15	9.5	111.9	1.6	30	4	94.2	7.5	9	6	42.4	1.5	12.5	6.5	63.8	1.9	15	15	176.6	1.0	10	2.5	19.6	4.0	5	2.5	9.8	2.0
15	9.5	111.9	1.6	30	4	94.2	7.5	9	6	42.4	1.5	12.5	6.5	63.8	1.9	15	15	176.6	1.0	10	2.5	19.6	4.0	10	9	70.7	1.1
15	9.5	111.9	1.6	30	4	94.2	7.5	9	6	42.4	1.5	12.5	6.5	63.8	1.9	15	15	176.6	1.0	10	2.5	19.6	4.0	10	9	70.7	1.1
15	9.5	111.9	1.6	30	4	94.2	7.5	9	6	42.4	1.5	12.5	6.5	63.8	1.9	15	15	176.6	1.0	10	2.5	19.6	4.0	10	9	70.7	1.1
15	9.5	111.9	1.6	30	4	94.2	7.5	9	6	42.4	1.5	12.5	6.5	63.8	1.9	15	15	176.6	1.0	10	2.5	19.6	4.0	10	9	70.7	1.1
15	9.5	111.9	1.6	25	5	98.1	5.0	11.5	7.5	67.7	1.5	12.5	6.5	63.8	1.9	15	15	176.6	1.0	10	2.5	19.6	4.0	10	9	70.7	1.1
15	9.5	111.9	1.6	25	5	98.1	5.0	11.5	7.5	67.7	1.5	12.5	6.5	63.8	1.9	15	15	176.6	1.0	10	2.5	19.6	4.0	10	9	70.7	1.1
15	9.5	111.9	1.6	25	5	98.1	5.0	11.5	7.5	67.7	1.5	12.5	6.5	63.8	1.9	15	15	176.6	1.0	15	15	176.6	1.0	10	9	70.7	1.1
17.5	5	68.7	3.5	25	5	98.1	5.0	11.5	7.5	67.7	1.5	12.5	6.5	63.8	1.9	15	15	176.6	1.0	15	15	176.6	1.0	10	9	70.7	1.1
17.5	5	68.7	3.5	25	5	98.1	5.0	11.5	7.5	67.7	1.5	12.5	6.5	63.8	1.9	15	15	176.6	1.0	15	15	176.6	1.0	10	9	70.7	1.1
17.5	5	68.7	3.5	25	5	98.1	5.0	11.5	7.5	67.7	1.5	12.5	6.5	63.8	1.9	15	15	176.6	1.0	15	15	176.6	1.0	10	9	70.7	1.1
17.5	5	68.7	3.5	25	5	98.1	5.0	11.5	7.5	67.7	1.5	10	7.5	58.9	1.3	10	9.5	74.6	1.1	15	15	176.6	1.0	10	9	70.7	1.1

17.5	5	68.7	3.5	25	5	98.1	5.0	11.5	7.5	67.7	1.5	10	7.5	58.9	1.3	10	9.5	74.6	1.1	15	15	176.6	1.0	10	9	70.7	1.1
17.5	5	68.7	3.5	25	5	98.1	5.0	11.5	7.5	67.7	1.5	10	7.5	58.9	1.3	10	9.5	74.6	1.1	15	15	176.6	1.0	10	9	70.7	1.1
17.5	5	68.7	3.5	25	5	98.1	5.0	11.5	7.5	67.7	1.5	10	7.5	58.9	1.3	10	9.5	74.6	1.1	15	15	176.6	1.0	22.5	22.5	397.4	1.0
17.5	5	68.7	3.5	25	5	98.1	5.0	11.5	7.5	67.7	1.5	10	7.5	58.9	1.3	10	9.5	74.6	1.1	15	15	176.6	1.0	22.5	22.5	397.4	1.0
17.5	5	68.7	3.5	25	5	98.1	5.0	11.5	7.5	67.7	1.5	10	7.5	58.9	1.3	10	9.5	74.6	1.1	15	15	176.6	1.0	22.5	22.5	397.4	1.0
17.5	5	68.7	3.5	25	5	98.1	5.0	11.5	7.5	67.7	1.5	10	7.5	58.9	1.3	10	9.5	74.6	1.1	15	15	176.6	1.0	22.5	22.5	397.4	1.0
17.5	5	68.7	3.5	25	5	98.1	5.0	11.5	7.5	67.7	1.5	10	7.5	58.9	1.3	10	9.5	74.6	1.1	15	15	176.6	1.0	22.5	22.5	397.4	1.0
17.5	5	68.7	3.5	25	5	98.1	5.0	11.5	7.5	67.7	1.5	10	7.5	58.9	1.3	10	9.5	74.6	1.1	15	15	176.6	1.0	22.5	22.5	397.4	1.0
17.5	5	68.7	3.5	25	5	98.1	5.0	11.5	7.5	67.7	1.5	10	7.5	58.9	1.3	10	9.5	74.6	1.1	15	15	176.6	1.0	22.5	22.5	397.4	1.0

Table J. Osmotic increase in the *C. albicans* NCPF 3153 cell size upon C1 treatment. Results from this table were used for preparing Fig 5 K in the main body of the paper.

Candida albicans											
**Control cells 30 min in medium**			**Control cells 30 min in water**			**C1-treated cells 30 min in medium**			**C1- treated cells 30 min in water**		
**Diameter A [µm]**	**Diameter B [µm]**	**Cell size [µm^2]**	**Diameter A [µm]**	**Diameter B [µm]**	**Cell size [µm^2]**	**Diameter A [µm]**	**Diameter B [µm]**	**Cell size [µm^2]**	**Diameter A [µm]**	**Diameter B [µm]**	**Cell size [µm^2]**
4	4	12.6	3.5	4	11.0	10	10	78.5	15	16	188.4
4	4	12.6	3.5	4	11.0	10	10	78.5	15	16	188.4
4	4	12.6	3.5	4	11.0	10	10	78.5	15	16	188.4
4	4	12.6	3.5	4	11.0	10	10	78.5	15	16	188.4
4	4	12.6	3.5	4	11.0	10	10	78.5	15	16	188.4
4	4	12.6	3.5	4	11.0	10	10	78.5	15	16	188.4
4	4	12.6	3.5	4	11.0	10	10	78.5	15	16	188.4
4	4	12.6	3.5	4	11.0	10	10	78.5	15	16	188.4
4	4	12.6	3.5	4	11.0	10	10	78.5	15	16	188.4
4	4	12.6	3.5	4	11.0	10	10	78.5	15	16	188.4
4	4	12.6	3.5	4	11.0	10	10	78.5	15	16	188.4
4	4	12.6	3.5	4	11.0	10	10	78.5	15	16	188.4
4	4	12.6	3.5	4	11.0	10	10	78.5	15	16	188.4
4	4	12.6	3.5	4	11.0	10	10	78.5	15	16	188.4
4	4	12.6	3.5	4	11.0	10	10	78.5	15	16	188.4
4	4	12.6	3.5	4	11.0	10	10	78.5	15	16	188.4
4	4	12.6	3.5	4	11.0	10	10	78.5	15	16	188.4
4	4	12.6	3.5	4	11.0	10	10	78.5	15	16	188.4
4	4	12.6	3.5	4	11.0	10	10	78.5	15	16	188.4
4	4	12.6	3.5	4	11.0	12	12	113.0	20	20	314.0
3.5	4	11.0	3.5	4	11.0	12	12	113.0	20	20	314.0
3.5	4	11.0	4.5	4	14.1	12	12	113.0	20	20	314.0
3.5	4	11.0	4.5	4	14.1	12	12	113.0	20	20	314.0

3.5	4	11.0	4.5	4	14.1	12	12	113.0	20	20	314.0
3.5	4	11.0	4.5	4	14.1	12	12	113.0	20	20	314.0
3.5	4	11.0	4.5	4	14.1	12	12	113.0	20	20	314.0
3.5	4	11.0	4.5	4	14.1	12	12	113.0	20	20	314.0
3.5	4	11.0	4.5	4	14.1	12	12	113.0	20	20	314.0
3.5	4	11.0	4.5	4	14.1	12	12	113.0	20	20	314.0
3.5	4	11.0	4.5	4	14.1	12	12	113.0	20	20	314.0
3.5	4	11.0	4.5	4	14.1	12	12	113.0	20	20	314.0
3.5	4	11.0	4.5	4	14.1	12	12	113.0	20	20	314.0
3.5	4	11.0	4.5	4	14.1	12	12	113.0	20	20	314.0
3.5	4	11.0	4.5	4	14.1	12	12	113.0	26	26	530.7
3.5	4	11.0	4.5	4	14.1	12	12	113.0	26	26	530.7
3.5	4	11.0	4.5	4	14.1	12	12	113.0	26	26	530.7
3.5	4	11.0	4.5	4	14.1	12	12	113.0	26	26	530.7
3.5	4	11.0	4.5	4	14.1	12	12	113.0	26	26	530.7
3.5	4	11.0	4.5	4	14.1	12	12	113.0	26	26	530.7
3.5	4	11.0	4.5	4	14.1	12	12	113.0	26	26	530.7
3.5	4	11.0	4.5	4	14.1	12	12	113.0	26	26	530.7
3.5	4	11.0	4.5	4	14.1	12	12	113.0	26	26	530.7
3.5	4	11.0	4.5	4	14.1	12	12	113.0	26	26	530.7
3.5	4	11.0	4.5	4	14.1	12	12	113.0	26	26	530.7
3.5	4	11.0	4.5	4	14.1	12	12	113.0	26	26	530.7
3.5	4	11.0	4.5	4	14.1	9	8.5	60.1	26	26	530.7
3.5	4	11.0	4.5	4	14.1	9	8.5	60.1	26	26	530.7
3	3	7.1	5	5.5	21.6	9	8.5	60.1	26	26	530.7
3	3	7.1	5	5.5	21.6	9	8.5	60.1	26	26	530.7
3	3	7.1	5	5.5	21.6	9	8.5	60.1	26	26	530.7
3	3	7.1	5	5.5	21.6	9	8.5	60.1	26	26	530.7
3	3	7.1	5	5.5	21.6	9	8.5	60.1	26	26	530.7
3	3	7.1	5	5.5	21.6	9	8.5	60.1	26	26	530.7
3	3	7.1	5	5.5	21.6	9	8.5	60.1	30	29	683.0
3	3	7.1	5	5.5	21.6	9	8.5	60.1	30	29	683.0
3	3	7.1	5	5.5	21.6	9	8.5	60.1	30	29	683.0
3	3	7.1	5	5.5	21.6	9	8.5	60.1	30	29	683.0
3	3	7.1	5	5.5	21.6	9	8.5	60.1	30	29	683.0
3	3	7.1	5	5.5	21.6	9	8.5	60.1	30	29	683.0

3	3	7.1	5	5.5	21.6	9	9	63.6	30	29	683.0
3	3	7.1	5	5.5	21.6	9	9	63.6	30	29	683.0
3	3	7.1	5	5.5	21.6	9	9	63.6	30	29	683.0
3	3	7.1	5	5.5	21.6	9	9	63.6	30	29	683.0
3	3	7.1	5	5.5	21.6	9	9	63.6	30	29	683.0
3	3	7.1	5	5.5	21.6	9	9	63.6	30	29	683.0
3	3	7.1	5	5.5	21.6	9	9	63.6	30	29	683.0
3	3	7.1	5	5.5	21.6	9	9	63.6	30	29	683.0
3	3	7.1	5	5.5	21.6	9	9	63.6	30	29	683.0
3	3	7.1	5	5.5	21.6	9	9	63.6	30	29	683.0
3	3.5	8.2	5	5.5	21.6	9	9	63.6	30	29	683.0
3	3.5	8.2	5	5.5	21.6	9	9	63.6	30	29	683.0
3	3.5	8.2	5	5.5	21.6	9	9	63.6	30	29	683.0
3	3.5	8.2	5	5.5	21.6	9	9	63.6	30	29	683.0
3	3.5	8.2	5	5.5	21.6	9	9	63.6	30	29	683.0
3	3.5	8.2	5	5.5	21.6	9	9	63.6	30	29	683.0
3	3.5	8.2	5	5.5	21.6	12.5	12.5	122.7	30	29	683.0
3	3.5	8.2	5	5.5	21.6	12.5	12.5	122.7	30	29	683.0
3	3.5	8.2	5	5.5	21.6	12.5	12.5	122.7	30	29	683.0
3	3.5	8.2	5	5.5	21.6	12.5	12.5	122.7	30	29	683.0
3	3.5	8.2	4.5	5.5	19.4	12.5	12.5	122.7	30	29	683.0
3	3.5	8.2	4.5	5.5	19.4	12.5	12.5	122.7	31	30	730.1
3	3.5	8.2	4.5	5.5	19.4	12.5	12.5	122.7	31	30	730.1
3	3.5	8.2	4.5	5.5	19.4	12.5	12.5	122.7	31	30	730.1
3	3.5	8.2	4.5	5.5	19.4	12.5	12.5	122.7	31	30	730.1
3	3.5	8.2	4.5	5.5	19.4	12.5	12.5	122.7	31	30	730.1
3	3.5	8.2	4.5	5.5	19.4	12.5	12.5	122.7	31	30	730.1
3	3.5	8.2	4.5	5.5	19.4	12.5	12.5	122.7	31	30	730.1
4	4.5	14.1	4.5	5.5	19.4	12.5	12.5	122.7	31	30	730.1
4	4.5	14.1	4.5	5.5	19.4	12.5	12.5	122.7	31	30	730.1
4	4.5	14.1	4.5	5.5	19.4	12.5	12.5	122.7	31	30	730.1
4	4.5	14.1	4.5	5.5	19.4	12	12.5	117.8	31	30	730.1
4	4.5	14.1	4.5	5.5	19.4	12	12.5	117.8	17	17.5	233.5
4	4.5	14.1	4.5	5.5	19.4	12	12.5	117.8	17	17.5	233.5
4	4.5	14.1	4.5	5.5	19.4	12	12.5	117.8	17	17.5	233.5
4	4.5	14.1	4.5	5.5	19.4	12	12.5	117.8	17	17.5	233.5

4	4.5	14.1	4.5	5.5	19.4	12	12.5	117.8	17	17.5	233.5
4	4.5	14.1	4.5	5.5	19.4	12	12.5	117.8	17	17.5	233.5
4	4.5	14.1	4.5	5.5	19.4	12	12.5	117.8	17	17.5	233.5
4	4.5	14.1	4.5	5.5	19.4	12	12.5	117.8	17	17.5	233.5
4	4.5	14.1	4.5	5.5	19.4	12	12.5	117.8	17	17.5	233.5
4	4.5	14.1	4.5	5	17.7	2.5	2.5	4.9	17	17.5	233.5
4	4.5	14.1	4.5	5	17.7	2.5	2.5	4.9	17	17.5	233.5
4	4.5	14.1	4.5	5	17.7	2.5	2.5	4.9	17	17.5	233.5
4	4.5	14.1	4.5	5	17.7	2.5	2.5	4.9	17	17.5	233.5
4	4.5	14.1	4.5	5	17.7	2.5	2.5	4.9	17	17.5	233.5
4	4.5	14.1	4.5	5	17.7	2.5	2.5	4.9	9	9	63.6
4	4.5	14.1	4.5	5	17.7	2.5	2.5	4.9	9	9	63.6
4	4.5	14.1	4.5	5	17.7	2.5	2.5	4.9	9	9	63.6
4	4.5	14.1	4.5	5	17.7	2.5	2.5	4.9	9	9	63.6
4	4.5	14.1	4.5	5	17.7	2.5	2.5	4.9	9	9	63.6
4	4.5	14.1	4.5	5	17.7	2.5	2.5	4.9	9	9	63.6
4	4.5	14.1	4.5	5	17.7	2.5	2.5	4.9	9	9	63.6
4.5	4.5	15.9	4.5	5	17.7	2.5	2.5	4.9	9	9	63.6
4.5	4.5	15.9	4.5	5	17.7	2.5	2.5	4.9	9	9	63.6
4.5	4.5	15.9	4.5	5	17.7	2.5	2.5	4.9	9	9	63.6
4.5	4.5	15.9	4.5	5	17.7	2.5	2.5	4.9	9	9	63.6
4.5	4.5	15.9	3.5	4.5	12.4	2.5	2.5	4.9	9	9	63.6
4.5	4.5	15.9	3.5	4.5	12.4	2.5	2.5	4.9	9	9	63.6
4.5	4.5	15.9	3.5	4.5	12.4	2.5	2.5	4.9	9	9	63.6
4.5	4.5	15.9	3.5	4.5	12.4	2.5	2.5	4.9	9	9	63.6
4.5	4.5	15.9	3.5	4.5	12.4	2.5	2.5	4.9	9	9	63.6
4.5	4.5	15.9	3.5	4.5	12.4	2.5	2.5	4.9	9	9	63.6
4.5	4.5	15.9	3.5	4.5	12.4	2.5	2.5	4.9	9	9	63.6
4.5	4.5	15.9	3.5	4.5	12.4	2.5	2.5	4.9	9	9	63.6
4.5	4.5	15.9	3.5	4.5	12.4	2.5	2.5	4.9	9	9	63.6
4.5	4.5	15.9	3.5	4.5	12.4	2.5	2.5	4.9	9	9	63.6
4.5	4.5	15.9	3.5	4.5	12.4	2.5	2.5	4.9	6	6.5	30.6
4.5	4.5	15.9	3.5	4.5	12.4	2.5	2.5	4.9	6	6.5	30.6
4.5	4.5	15.9	3.5	4.5	12.4	2.5	2.5	4.9	6	6.5	30.6
4.5	4	14.1	3.5	4.5	12.4	2.5	2.5	4.9	6	6.5	30.6
4.5	4	14.1	3.5	4.5	12.4	2.5	2.5	4.9	6	6.5	30.6

4.5	4	14.1	3.5	4.5	12.4	3	2.5	5.9	6	6.5	30.6
4.5	4	14.1	3.5	4.5	12.4	3	2.5	5.9	6	6.5	30.6
4.5	4	14.1	3.5	4.5	12.4	3	2.5	5.9	6	6.5	30.6
4.5	4	14.1	3.5	4.5	12.4	3	2.5	5.9	6	6.5	30.6
4.5	4	14.1	3.5	4.5	12.4	3	2.5	5.9	6	6.5	30.6
4.5	4	14.1	3.5	4.5	12.4	3	2.5	5.9	6	6.5	30.6
4.5	4	14.1	3.5	4.5	12.4	3	2.5	5.9	6	6.5	30.6
4.5	4	14.1	3.5	4.5	12.4	3	2.5	5.9	6	6.5	30.6
4.5	4	14.1	6.5	6.5	33.2	3	2.5	5.9	6	6.5	30.6
4.5	4	14.1	6.5	6.5	33.2	3	2.5	5.9	6	6.5	30.6
4.5	4	14.1	6.5	6.5	33.2	3	2.5	5.9	6	6.5	30.6
4.5	4	14.1	6.5	6.5	33.2	3	2.5	5.9	6	6.5	30.6
4.5	4	14.1	6.5	6.5	33.2	3	2.5	5.9	6	6.5	30.6
4.5	4	14.1	6.5	6.5	33.2	3	2.5	5.9	6	6.5	30.6
4.5	4	14.1	6.5	6.5	33.2	3	2.5	5.9	6	6.5	30.6
4.5	4	14.1	6.5	6.5	33.2	3	2.5	5.9	6	6.5	30.6
4.5	4	14.1	6.5	6.5	33.2	3	2.5	5.9	6	6.5	30.6
4	4	12.6	6.5	6.5	33.2	3	2.5	5.9	7	7.5	41.2
4	4	12.6	6.5	6.5	33.2	3	2.5	5.9	7	7.5	41.2
4	4	12.6	6.5	6.5	33.2	3	2.5	5.9	7	7.5	41.2
4	4	12.6	6.5	6.5	33.2	3	2.5	5.9	7	7.5	41.2
4	4	12.6	6.5	6.5	33.2	2	2	3.1	7	7.5	41.2
4	4	12.6	6.5	6.5	33.2	2	2	3.1	7	7.5	41.2
4	4	12.6	6.5	6.5	33.2	2	2	3.1	7	7.5	41.2
4	4	12.6	6.5	6.5	33.2	2	2	3.1	7	7.5	41.2
4	4	12.6	6.5	6.5	33.2	2	2	3.1	7	7.5	41.2
4	4	12.6	6.5	6.5	33.2	2	2	3.1	7	7.5	41.2
4	4	12.6	6.5	6.5	33.2	2	2	3.1	7	7.5	41.2
4	4	12.6	6.5	6.5	33.2	2	2	3.1	7	7.5	41.2
4	4	12.6	6.5	6.5	33.2	2	2	3.1	7	7.5	41.2
4	4	12.6	6.5	6.5	33.2	2	2	3.1	7	7.5	41.2
4	4	12.6	6.5	6.5	33.2	2	2	3.1	7	7.5	41.2
4	4	12.6	6	6	28.3	2	2	3.1	7	7.5	41.2
4	4	12.6	6	6	28.3	2	2	3.1	7	7.5	41.2
4	4	12.6	6	6	28.3	2	2	3.1	7	7.5	41.2
4	4	12.6	6	6	28.3	2	2	3.1	7	7.5	41.2

4	4	12.6	6	6	28.3	2	2	3.1	7	7.5	41.2
4	4	12.6	6	6	28.3	2	2	3.1	7	7.5	41.2
4	4	12.6	6	6	28.3	2	2	3.1	7	7.5	41.2
4	3.5	11.0	6	6	28.3	2	2	3.1	7	7.5	41.2
4	3.5	11.0	6	6	28.3	2	2	3.1	7	7.5	41.2
4	3.5	11.0	6	6	28.3	2	14.5	22.8	14	14	153.9
4	3.5	11.0	6	6	28.3	2	14.5	22.8	14	14	153.9
4	3.5	11.0	6	6	28.3	2	14.5	22.8	14	14	153.9
4	3.5	11.0	6	6	28.3	2	14.5	22.8	14	14	153.9
4	3.5	11.0	6	6	28.3	2	14.5	22.8	14	14	153.9
4	3.5	11.0	6	6	28.3	2	14.5	22.8	14	14	153.9
4	3.5	11.0	6	6	28.3	2	14.5	22.8	14	14	153.9
4	3.5	11.0	6	6	28.3	2	14.5	22.8	14	14	153.9
4	3.5	11.0	6	6	28.3	15	14.5	170.7	14	14	153.9
4	3.5	11.0	6	6	28.3	15	14.5	170.7	14	14	153.9
4	3.5	11.0	6	6	28.3	15	14.5	170.7	14	14	153.9
4	3.5	11.0	6	6	28.3	15	14.5	170.7	14	14	153.9
4	3.5	11.0	6	6	28.3	15	14.5	170.7	14	14	153.9
4	3.5	11.0	7	6.5	35.7	14.5	14.5	165.0	14	14	153.9
4	3.5	11.0	7	6.5	35.7	14.5	14.5	165.0	14	14	153.9
4	3.5	11.0	7	6.5	35.7	14.5	14.5	165.0	14	14	153.9
4	3.5	11.0	7	6.5	35.7	14.5	14.5	165.0	14	14	153.9
4	3.5	11.0	7	6.5	35.7	14.5	14.5	165.0	14	14	153.9
4	3.5	11.0	7	6.5	35.7	14.5	14.5	165.0	16	16	201.0
4	3.5	11.0	7	6.5	35.7	14.5	14.5	165.0	16	16	201.0
4	3.5	11.0	7	6.5	35.7	14.5	14.5	165.0	16	16	201.0
4	3.5	11.0	7	6.5	35.7	14.5	14.5	165.0	16	16	201.0
4	3.5	11.0	7	6.5	35.7	14.5	14.5	165.0	16	16	201.0
4	3.5	11.0	7	6.5	35.7	14.5	14.5	165.0	16	16	201.0
4	3.5	11.0	7	6.5	35.7	14.5	14.5	165.0	16	16	201.0
4	3.5	11.0	7	6.5	35.7	14.5	14.5	165.0	16	16	201.0
4	3.5	11.0	7	6.5	35.7	14.5	14.5	165.0	16	16	201.0
4	3.5	11.0	7	6.5	35.7	14.5	14.5	165.0	16	16	201.0

Table K. Osmotic increase in the *C. parapsilosis* ATCC 22019 cell size upon C1 treatment Results from this table were used for preparing Fig 6 I in the main body of the paper.

Candida parapsilosis															
**Control cells 30 min in medium**				**Control cells 30 min in water**				**C1-treated cells 30 min in medium**				**C1- treated cells 30 min in water**			
**Diameter A [µm]**	**Diameter B [µm]**	**Cell size [µm²]**	**A/B**	**Diameter A [µm]**	**Diameter B [µm]**	**Cell size [µm²]**	**A/B**	**Diameter A [µm]**	**Diameter B [µm]**	**Cell size [µm²]**	**A/B**	**Diameter A [µm]**	**Diameter B [µm]**	**Cell size [µm²]**	**A/B**
20	5	78.5	4.0	20	4.5	70.7	4.4	5	5	19.6	1.0	17.5	15	206.1	1.2
20	5	78.5	4.0	20	4.5	70.7	4.4	5	5	19.6	1.0	17.5	15	206.1	1.2
20	5	78.5	4.0	20	4.5	70.7	4.4	5	5	19.6	1.0	17.5	15	206.1	1.2
20	5	78.5	4.0	20	4.5	70.7	4.4	5	5	19.6	1.0	17.5	15	206.1	1.2
20	5	78.5	4.0	20	4.5	70.7	4.4	5	5	19.6	1.0	17.5	15	206.1	1.2
20	5	78.5	4.0	20	4.5	70.7	4.4	5	5	19.6	1.0	17.5	15	206.1	1.2
20	5	78.5	4.0	20	4.5	70.7	4.4	5	5	19.6	1.0	17.5	15	206.1	1.2
20	5	78.5	4.0	20	4.5	70.7	4.4	5	5	19.6	1.0	17.5	15	206.1	1.2
20	5	78.5	4.0	20	4.5	70.7	4.4	5	5	19.6	1.0	17.5	15	206.1	1.2
20	5	78.5	4.0	20	4.5	70.7	4.4	5	5	19.6	1.0	17.5	15	206.1	1.2
20	5	78.5	4.0	20	4.5	70.7	4.4	5	5	19.6	1.0	17.5	15	206.1	1.2
20	5	78.5	4.0	20	4.5	70.7	4.4	5	5	19.6	1.0	17.5	15	206.1	1.2
20	5	78.5	4.0	20	4.5	70.7	4.4	5	5	19.6	1.0	17.5	15	206.1	1.2
20	5	78.5	4.0	20	4.5	70.7	4.4	5	5	19.6	1.0	17.5	15	206.1	1.2
20	5	78.5	4.0	20	4.5	70.7	4.4	5	5	19.6	1.0	17.5	15	206.1	1.2
20	5	78.5	4.0	20	4.5	70.7	4.4	5	5	19.6	1.0	17.5	15	206.1	1.2
20	5	78.5	4.0	20	4.5	70.7	4.4	5	5	19.6	1.0	17.5	15	206.1	1.2
20	5	78.5	4.0	20	4.5	70.7	4.4	5	5	19.6	1.0	17.5	15	206.1	1.2
20	5	78.5	4.0	20	4.5	70.7	4.4	5	5	19.6	1.0	17.5	15	206.1	1.2
20	5	78.5	4.0	20	4.5	70.7	4.4	5	5	19.6	1.0	17.5	15	206.1	1.2
20	5	78.5	4.0	20	4.5	70.7	4.4	5	5	19.6	1.0	17.5	15	206.1	1.2
20	5	78.5	4.0	20	4.5	70.7	4.4	5	5	19.6	1.0	17.5	15	206.1	1.2

20	5	78.5	4.0	20	4.5	70.7	4.4	5	5	19.6	1.0	17.5	15	206.1	1.2
20	5	78.5	4.0	20	4.5	70.7	4.4	5	5	19.6	1.0	17.5	15	206.1	1.2
20	5	78.5	4.0	17.5	5.5	75.6	3.2	5	5	19.6	1.0	17.5	15	206.1	1.2
20	5	78.5	4.0	17.5	5.5	75.6	3.2	5	5	19.6	1.0	17.5	15	206.1	1.2
20	5	78.5	4.0	17.5	5.5	75.6	3.2	20	20	314.0	1.0	17.5	15	206.1	1.2
30	4	94.2	7.5	17.5	5.5	75.6	3.2	20	20	314.0	1.0	17.5	15	206.1	1.2
30	4	94.2	7.5	17.5	5.5	75.6	3.2	20	20	314.0	1.0	17.5	15	206.1	1.2
30	4	94.2	7.5	17.5	5.5	75.6	3.2	20	20	314.0	1.0	22.5	21.5	379.7	1.0
30	4	94.2	7.5	17.5	5.5	75.6	3.2	20	20	314.0	1.0	22.5	21.5	379.7	1.0
30	4	94.2	7.5	17.5	5.5	75.6	3.2	20	20	314.0	1.0	22.5	21.5	379.7	1.0
30	4	94.2	7.5	17.5	5.5	75.6	3.2	20	20	314.0	1.0	22.5	21.5	379.7	1.0
30	4	94.2	7.5	17.5	5.5	75.6	3.2	20	20	314.0	1.0	22.5	21.5	379.7	1.0
30	4	94.2	7.5	17.5	5.5	75.6	3.2	20	20	314.0	1.0	22.5	21.5	379.7	1.0
30	4	94.2	7.5	17.5	5.5	75.6	3.2	20	20	314.0	1.0	22.5	21.5	379.7	1.0
30	4	94.2	7.5	17.5	5.5	75.6	3.2	20	20	314.0	1.0	22.5	21.5	379.7	1.0
30	4	94.2	7.5	17.5	5.5	75.6	3.2	20	20	314.0	1.0	22.5	21.5	379.7	1.0
30	4	94.2	7.5	17.5	5.5	75.6	3.2	20	20	314.0	1.0	22.5	21.5	379.7	1.0
30	4	94.2	7.5	17.5	5.5	75.6	3.2	20	20	314.0	1.0	22.5	21.5	379.7	1.0
30	4	94.2	7.5	17.5	5.5	75.6	3.2	20	20	314.0	1.0	22.5	21.5	379.7	1.0
30	4	94.2	7.5	17.5	5.5	75.6	3.2	20	20	314.0	1.0	22.5	21.5	379.7	1.0
30	4	94.2	7.5	12.5	6	58.9	2.1	20	20	314.0	1.0	22.5	21.5	379.7	1.0
30	4	94.2	7.5	12.5	6	58.9	2.1	20	20	314.0	1.0	22.5	21.5	379.7	1.0
30	4	94.2	7.5	12.5	6	58.9	2.1	20	20	314.0	1.0	22.5	21.5	379.7	1.0
30	4	94.2	7.5	12.5	6	58.9	2.1	20	20	314.0	1.0	22.5	21.5	379.7	1.0
12.5	4	39.3	3.1	12.5	6	58.9	2.1	20	20	314.0	1.0	22.5	21.5	379.7	1.0
12.5	4	39.3	3.1	12.5	6	58.9	2.1	20	20	314.0	1.0	22.5	21.5	379.7	1.0
12.5	4	39.3	3.1	12.5	6	58.9	2.1	20	20	314.0	1.0	25	25	490.6	1.0
12.5	4	39.3	3.1	12.5	6	58.9	2.1	20	20	314.0	1.0	25	25	490.6	1.0
12.5	4	39.3	3.1	12.5	6	58.9	2.1	20	20	314.0	1.0	25	25	490.6	1.0
12.5	4	39.3	3.1	12.5	6	58.9	2.1	20	20	314.0	1.0	25	25	490.6	1.0
12.5	4	39.3	3.1	12.5	6	58.9	2.1	20	20	314.0	1.0	25	25	490.6	1.0
12.5	4	39.3	3.1	12.5	6	58.9	2.1	20	20	314.0	1.0	25	25	490.6	1.0
12.5	4	39.3	3.1	12.5	6	58.9	2.1	20	20	314.0	1.0	25	25	490.6	1.0
12.5	4	39.3	3.1	12.5	6	58.9	2.1	20	20	314.0	1.0	25	25	490.6	1.0
12.5	4	39.3	3.1	12.5	6	58.9	2.1	20	20	314.0	1.0	25	25	490.6	1.0
12.5	4	39.3	3.1	12.5	6	58.9	2.1	20	20	314.0	1.0	25	25	490.6	1.0

12.5	4	39.3	3.1	12.5	6	58.9	2.1	20	20	314.0	1.0	25	25	490.6	1.0
12.5	4	39.3	3.1	12.5	6	58.9	2.1	20	20	314.0	1.0	25	25	490.6	1.0
12.5	4	39.3	3.1	12.5	6	58.9	2.1	20	20	314.0	1.0	25	25	490.6	1.0
12.5	4	39.3	3.1	12.5	6	58.9	2.1	20	20	314.0	1.0	25	25	490.6	1.0
12.5	4	39.3	3.1	12.5	6	58.9	2.1	20	20	314.0	1.0	25	25	490.6	1.0
12.5	4	39.3	3.1	12.5	6	58.9	2.1	20	20	314.0	1.0	25	25	490.6	1.0
12.5	4	39.3	3.1	12.5	6	58.9	2.1	17.5	17.5	240.4	1.0	25	25	490.6	1.0
25	3.5	68.7	7.1	12.5	6	58.9	2.1	17.5	17.5	240.4	1.0	21.5	21	354.4	1.0
25	3.5	68.7	7.1	30	5.5	129.5	5.5	17.5	17.5	240.4	1.0	21.5	21	354.4	1.0
25	3.5	68.7	7.1	30	5.5	129.5	5.5	17.5	17.5	240.4	1.0	21.5	21	354.4	1.0
25	3.5	68.7	7.1	30	5.5	129.5	5.5	17.5	17.5	240.4	1.0	21.5	21	354.4	1.0
25	3.5	68.7	7.1	30	5.5	129.5	5.5	17.5	17.5	240.4	1.0	21.5	21	354.4	1.0
25	3.5	68.7	7.1	30	5.5	129.5	5.5	17.5	17.5	240.4	1.0	21.5	21	354.4	1.0
25	3.5	68.7	7.1	30	5.5	129.5	5.5	17.5	17.5	240.4	1.0	21.5	21	354.4	1.0
25	3.5	68.7	7.1	30	5.5	129.5	5.5	17.5	17.5	240.4	1.0	21.5	21	354.4	1.0
25	3.5	68.7	7.1	30	5.5	129.5	5.5	17.5	17.5	240.4	1.0	21.5	21	354.4	1.0
25	3.5	68.7	7.1	30	5.5	129.5	5.5	17.5	17.5	240.4	1.0	21.5	21	354.4	1.0
25	3.5	68.7	7.1	30	5.5	129.5	5.5	17.5	17.5	240.4	1.0	21.5	21	354.4	1.0
25	3.5	68.7	7.1	30	5.5	129.5	5.5	17.5	17.5	240.4	1.0	21.5	21	354.4	1.0
25	3.5	68.7	7.1	30	5.5	129.5	5.5	17.5	17.5	240.4	1.0	21.5	21	354.4	1.0
25	3.5	68.7	7.1	30	5.5	129.5	5.5	17.5	17.5	240.4	1.0	21.5	21	354.4	1.0
22.5	5	88.3	4.5	30	5.5	129.5	5.5	17.5	17.5	240.4	1.0	21.5	21	354.4	1.0
22.5	5	88.3	4.5	30	5.5	129.5	5.5	17.5	17.5	240.4	1.0	21.5	21	354.4	1.0
22.5	5	88.3	4.5	30	5.5	129.5	5.5	17.5	17.5	240.4	1.0	21.5	21	354.4	1.0
22.5	5	88.3	4.5	30	5.5	129.5	5.5	17.5	17.5	240.4	1.0	21.5	21	354.4	1.0
22.5	5	88.3	4.5	30	5.5	129.5	5.5	17.5	17.5	240.4	1.0	21.5	21	354.4	1.0
22.5	5	88.3	4.5	30	5.5	129.5	5.5	17.5	17.5	240.4	1.0	30	28	659.4	1.1
22.5	5	88.3	4.5	30	5.5	129.5	5.5	17.5	17.5	240.4	1.0	30	28	659.4	1.1
22.5	5	88.3	4.5	30	5.5	129.5	5.5	17.5	17.5	240.4	1.0	30	28	659.4	1.1
22.5	5	88.3	4.5	30	5.5	129.5	5.5	17.5	17.5	240.4	1.0	30	28	659.4	1.1
22.5	5	88.3	4.5	30	5.5	129.5	5.5	17.5	17.5	240.4	1.0	30	28	659.4	1.1
22.5	5	88.3	4.5	25	5	98.1	5.0	17.5	17.5	240.4	1.0	30	28	659.4	1.1
22.5	5	88.3	4.5	25	5	98.1	5.0	17.5	17.5	240.4	1.0	30	28	659.4	1.1
22.5	5	88.3	4.5	25	5	98.1	5.0	17.5	17.5	240.4	1.0	30	28	659.4	1.1
22.5	5	88.3	4.5	25	5	98.1	5.0	17.5	17.5	240.4	1.0	30	28	659.4	1.1
22.5	5	88.3	4.5	25	5	98.1	5.0	17.5	17.5	240.4	1.0	30	28	659.4	1.1

15	2.5	29.4	6.0	25	5	98.1	5.0	17.5	17.5	240.4	1.0	30	28	659.4	1.1
15	2.5	29.4	6.0	25	5	98.1	5.0	17.5	17.5	240.4	1.0	30	28	659.4	1.1
15	2.5	29.4	6.0	25	5	98.1	5.0	17.5	17.5	240.4	1.0	30	28	659.4	1.1
15	2.5	29.4	6.0	25	5	98.1	5.0	17.5	17.5	240.4	1.0	30	28	659.4	1.1
15	2.5	29.4	6.0	25	5	98.1	5.0	17.5	17.5	240.4	1.0	30	28	659.4	1.1
15	2.5	29.4	6.0	25	5	98.1	5.0	17.5	17.5	240.4	1.0	30	28	659.4	1.1
15	2.5	29.4	6.0	25	5	98.1	5.0	17.5	17.5	240.4	1.0	30	28	659.4	1.1
15	2.5	29.4	6.0	25	5	98.1	5.0	17.5	17.5	240.4	1.0	30	28	659.4	1.1
15	2.5	29.4	6.0	25	5	98.1	5.0	17.5	17.5	240.4	1.0	30	28	659.4	1.1
15	2.5	29.4	6.0	25	5	98.1	5.0	13.5	13	137.8	1.0	30	28	659.4	1.1
15	2.5	29.4	6.0	25	5	98.1	5.0	13.5	13	137.8	1.0	30	28	659.4	1.1
15	2.5	29.4	6.0	25	5	98.1	5.0	13.5	13	137.8	1.0	30	28	659.4	1.1
15	2.5	29.4	6.0	25	5	98.1	5.0	13.5	13	137.8	1.0	22.5	22	388.6	1.0
15	2.5	29.4	6.0	25	5	98.1	5.0	13.5	13	137.8	1.0	22.5	22	388.6	1.0
15	2.5	29.4	6.0	25	5	98.1	5.0	13.5	13	137.8	1.0	22.5	22	388.6	1.0
15	2.5	29.4	6.0	25	5	98.1	5.0	13.5	13	137.8	1.0	22.5	22	388.6	1.0
15	2.5	29.4	6.0	25	5	98.1	5.0	13.5	13	137.8	1.0	22.5	22	388.6	1.0
15	2.5	29.4	6.0	22.5	4.5	79.5	5.0	13.5	13	137.8	1.0	22.5	22	388.6	1.0
15	2.5	29.4	6.0	22.5	4.5	79.5	5.0	13.5	13	137.8	1.0	22.5	22	388.6	1.0
15	2.5	29.4	6.0	22.5	4.5	79.5	5.0	13.5	13	137.8	1.0	22.5	22	388.6	1.0
15	2.5	29.4	6.0	22.5	4.5	79.5	5.0	13.5	13	137.8	1.0	22.5	22	388.6	1.0
15	2.5	29.4	6.0	22.5	4.5	79.5	5.0	13.5	13	137.8	1.0	22.5	22	388.6	1.0
15	2.5	29.4	6.0	22.5	4.5	79.5	5.0	13.5	13	137.8	1.0	22.5	22	388.6	1.0
15	2.5	29.4	6.0	22.5	4.5	79.5	5.0	13.5	13	137.8	1.0	22.5	22	388.6	1.0
15	2.5	29.4	6.0	22.5	4.5	79.5	5.0	13.5	13	137.8	1.0	22.5	22	388.6	1.0
15	2.5	29.4	6.0	22.5	4.5	79.5	5.0	13.5	13	137.8	1.0	22.5	22	388.6	1.0
15	2.5	29.4	6.0	22.5	4.5	79.5	5.0	13.5	13	137.8	1.0	22.5	22	388.6	1.0
15	2.5	29.4	6.0	22.5	4.5	79.5	5.0	13.5	13	137.8	1.0	22.5	22	388.6	1.0
20	3	47.1	6.7	22.5	4.5	79.5	5.0	13.5	13	137.8	1.0	22.5	22	388.6	1.0
20	3	47.1	6.7	22.5	4.5	79.5	5.0	13.5	13	137.8	1.0	22.5	22	388.6	1.0
20	3	47.1	6.7	22.5	4.5	79.5	5.0	13.5	13	137.8	1.0	22.5	22	388.6	1.0
20	3	47.1	6.7	22.5	4.5	79.5	5.0	13.5	13	137.8	1.0	22.5	22	388.6	1.0
20	3	47.1	6.7	22.5	4.5	79.5	5.0	15	15	176.6	1.0	22.5	22	388.6	1.0
20	3	47.1	6.7	22.5	4.5	79.5	5.0	15	15	176.6	1.0	18.5	17	246.9	1.1
20	3	47.1	6.7	22.5	4.5	79.5	5.0	15	15	176.6	1.0	18.5	17	246.9	1.1
20	3	47.1	6.7	22.5	4.5	79.5	5.0	15	15	176.6	1.0	18.5	17	246.9	1.1

20	3	47.1	6.7	22.5	4.5	79.5	5.0	15	15	176.6	1.0	18.5	17	246.9	1.1
20	3	47.1	6.7	22.5	4.5	79.5	5.0	15	15	176.6	1.0	18.5	17	246.9	1.1
20	3	47.1	6.7	22.5	4.5	79.5	5.0	15	15	176.6	1.0	18.5	17	246.9	1.1
20	3	47.1	6.7	22.5	4.5	79.5	5.0	15	15	176.6	1.0	18.5	17	246.9	1.1
20	3	47.1	6.7	15	2.5	29.4	6.0	15	15	176.6	1.0	18.5	17	246.9	1.1
20	3	47.1	6.7	15	2.5	29.4	6.0	15	15	176.6	1.0	18.5	17	246.9	1.1
20	3	47.1	6.7	15	2.5	29.4	6.0	15	15	176.6	1.0	18.5	17	246.9	1.1
20	3	47.1	6.7	15	2.5	29.4	6.0	15	15	176.6	1.0	18.5	17	246.9	1.1
20	3	47.1	6.7	15	2.5	29.4	6.0	15	15	176.6	1.0	18.5	17	246.9	1.1
20	3	47.1	6.7	15	2.5	29.4	6.0	15	15	176.6	1.0	18.5	17	246.9	1.1
20	3	47.1	6.7	15	2.5	29.4	6.0	15	15	176.6	1.0	18.5	17	246.9	1.1
20	3	47.1	6.7	15	2.5	29.4	6.0	15	15	176.6	1.0	18.5	17	246.9	1.1
20	3	47.1	6.7	15	2.5	29.4	6.0	15	15	176.6	1.0	18.5	17	246.9	1.1
20	3	47.1	6.7	15	2.5	29.4	6.0	15	15	176.6	1.0	18.5	17	246.9	1.1
20	3	47.1	6.7	15	2.5	29.4	6.0	15	15	176.6	1.0	18.5	17	246.9	1.1
20	3	47.1	6.7	15	2.5	29.4	6.0	15	15	176.6	1.0	18.5	17	246.9	1.1
20	3	47.1	6.7	15	2.5	29.4	6.0	18	17.5	247.3	1.0	18.5	17	246.9	1.1
20	3	47.1	6.7	15	2.5	29.4	6.0	18	17.5	247.3	1.0	18.5	17	246.9	1.1
20	3	47.1	6.7	15	2.5	29.4	6.0	18	17.5	247.3	1.0	18.5	17	246.9	1.1
20	3	47.1	6.7	15	2.5	29.4	6.0	18	17.5	247.3	1.0	18.5	17	246.9	1.1
20	3	47.1	6.7	15	2.5	29.4	6.0	18	17.5	247.3	1.0	18.5	17	246.9	1.1
20	3	47.1	6.7	15	2.5	29.4	6.0	18	17.5	247.3	1.0	12.5	12.5	122.7	1.0
20	3	47.1	6.7	15	2.5	29.4	6.0	18	17.5	247.3	1.0	12.5	12.5	122.7	1.0
30	4	94.2	7.5	15	2.5	29.4	6.0	18	17.5	247.3	1.0	12.5	12.5	122.7	1.0
30	4	94.2	7.5	15	2.5	29.4	6.0	18	17.5	247.3	1.0	12.5	12.5	122.7	1.0
30	4	94.2	7.5	15	2.5	29.4	6.0	18	17.5	247.3	1.0	12.5	12.5	122.7	1.0
30	4	94.2	7.5	20	5	78.5	4.0	18	17.5	247.3	1.0	12.5	12.5	122.7	1.0
30	4	94.2	7.5	20	5	78.5	4.0	18	17.5	247.3	1.0	12.5	12.5	122.7	1.0
30	4	94.2	7.5	20	5	78.5	4.0	18	17.5	247.3	1.0	12.5	12.5	122.7	1.0
30	4	94.2	7.5	20	5	78.5	4.0	18	17.5	247.3	1.0	12.5	12.5	122.7	1.0
30	4	94.2	7.5	20	5	78.5	4.0	18	17.5	247.3	1.0	12.5	12.5	122.7	1.0
30	4	94.2	7.5	20	5	78.5	4.0	18	17.5	247.3	1.0	12.5	12.5	122.7	1.0
30	4	94.2	7.5	20	5	78.5	4.0	18	17.5	247.3	1.0	12.5	12.5	122.7	1.0
30	4	94.2	7.5	20	5	78.5	4.0	18	17.5	247.3	1.0	12.5	12.5	122.7	1.0
30	4	94.2	7.5	20	5	78.5	4.0	18	17.5	247.3	1.0	12.5	12.5	122.7	1.0
30	4	94.2	7.5	20	5	78.5	4.0	18	17.5	247.3	1.0	12.5	12.5	122.7	1.0

30	4	94.2	7.5	20	5	78.5	4.0	18	17.5	247.3	1.0	12.5	12.5	122.7	1.0
30	4	94.2	7.5	20	5	78.5	4.0	18	17.5	247.3	1.0	12.5	12.5	122.7	1.0
30	4	94.2	7.5	20	5	78.5	4.0	18	17.5	247.3	1.0	12.5	12.5	122.7	1.0
30	4	94.2	7.5	20	5	78.5	4.0	18	17.5	247.3	1.0	12.5	12.5	122.7	1.0
30	4	94.2	7.5	20	5	78.5	4.0	18	17.5	247.3	1.0	12.5	12.5	122.7	1.0
30	4	94.2	7.5	20	5	78.5	4.0	18	17.5	247.3	1.0	12.5	12.5	122.7	1.0
30	4	94.2	7.5	20	5	78.5	4.0	18	17.5	247.3	1.0	12.5	12.5	122.7	1.0
30	4	94.2	7.5	20	5	78.5	4.0	10	2.5	19.6	4.0	12.5	12.5	122.7	1.0
30	4	94.2	7.5	20	5	78.5	4.0	10	2.5	19.6	4.0	12.5	12.5	122.7	1.0
30	4	94.2	7.5	20	5	78.5	4.0	10	2.5	19.6	4.0	20	20	314.0	1.0
30	4	94.2	7.5	20	5	78.5	4.0	10	2.5	19.6	4.0	20	20	314.0	1.0
30	4	94.2	7.5	17.5	6	82.4	2.9	10	2.5	19.6	4.0	20	20	314.0	1.0
30	4	94.2	7.5	17.5	6	82.4	2.9	10	2.5	19.6	4.0	20	20	314.0	1.0
30	4	94.2	7.5	17.5	6	82.4	2.9	10	2.5	19.6	4.0	20	20	314.0	1.0
30	4	94.2	7.5	17.5	6	82.4	2.9	10	2.5	19.6	4.0	20	20	314.0	1.0
30	4	94.2	7.5	17.5	6	82.4	2.9	10	2.5	19.6	4.0	20	20	314.0	1.0
30	4	94.2	7.5	17.5	6	82.4	2.9	10	2.5	19.6	4.0	20	20	314.0	1.0
30	4	94.2	7.5	17.5	6	82.4	2.9	10	2.5	19.6	4.0	20	20	314.0	1.0
25	5	98.1	5.0	17.5	6	82.4	2.9	10	2.5	19.6	4.0	20	20	314.0	1.0
25	5	98.1	5.0	17.5	6	82.4	2.9	10	2.5	19.6	4.0	20	20	314.0	1.0
25	5	98.1	5.0	17.5	6	82.4	2.9	15	15	176.6	1.0	20	20	314.0	1.0
25	5	98.1	5.0	17.5	6	82.4	2.9	15	15	176.6	1.0	20	20	314.0	1.0
25	5	98.1	5.0	17.5	6	82.4	2.9	15	15	176.6	1.0	25.5	25	500.4	1.0
25	5	98.1	5.0	17.5	6	82.4	2.9	15	15	176.6	1.0	25.5	25	500.4	1.0
25	5	98.1	5.0	17.5	6	82.4	2.9	15	15	176.6	1.0	25.5	25	500.4	1.0
25	5	98.1	5.0	20	6	94.2	3.3	15	15	176.6	1.0	25.5	25	500.4	1.0
25	5	98.1	5.0	20	6	94.2	3.3	15	15	176.6	1.0	25.5	25	500.4	1.0
25	5	98.1	5.0	20	6	94.2	3.3	15	15	176.6	1.0	25.5	25	500.4	1.0
25	5	98.1	5.0	20	6	94.2	3.3	15	15	176.6	1.0	25.5	25	500.4	1.0
25	5	98.1	5.0	20	6	94.2	3.3	15	15	176.6	1.0	25.5	25	500.4	1.0
25	5	98.1	5.0	20	6	94.2	3.3	15	15	176.6	1.0	25.5	25	500.4	1.0
25	5	98.1	5.0	20	6	94.2	3.3	15	15	176.6	1.0	25.5	25	500.4	1.0
25	5	98.1	5.0	20	6	94.2	3.3	15	15	176.6	1.0	25.5	25	500.4	1.0
25	5	98.1	4.0	20	6	94.2	3.3	15	15	176.6	1.0	25.5	25	500.4	1.0

Table L. Enhanced sensitivity of C1-treated *C. albicans* NCPF 3153 cells to Calcofluor white, interfering with polymerization of the cell wall components. Results from this table were used for preparing Fig 5L in the main body of the paper.

Candida albicans						
	**Control**	**calcofluor (28 µg/ml)**	**C1 (8 µg/ml)**	**C1 (8 µg/ml) + calcofluor (28 µg/ml)**	**C1 (32 µg/ml)**	**C1 (32 µg/ml) + calcofluor (28 µg/ml)**
Optical density (OD $_{600}$)	1.140	1.154	0.710	0.242	0.434	0.112
	1.177	1.183	0.830	0.334	0.453	0.142
	1.218	1.154	0.830	0.121	0.315	0.142
	1.332	1.232	0.794	0.114	0.436	0.053
	1.243	1.343	0.760	0.311	0.457	0.142
	1.240	1.340	0.834	0.320	0.391	0.102
	1.243	1.254	0.872	0.110	0.345	0.121
	1.110	1.135	0.763	0.361	0.334	0.142
	1.215	1.135	0.842	0.455	0.332	0.113
	1.260	1.230	0.824	0.378	0.312	0.145
	1.212	1.241	0.862	0.354	0.467	0.082
	1.320	1.152	0.782	0.325	0.262	0.125
Mean	1.226	1.213	0.809	0.285	0.378	0.118
Standard deviation	0.064	0.074	0.048	0.114	0.070	0.028

Table M. Enhanced sensitivity of C1-treated *C. parapsilosis* ATCC 22019 cells to Calcofluor white, interfering with polymerization of the cell wall components. Results from this table were used for preparing Fig 6J in the main body of the paper.

Candida parapsilosis						
	**Control**	**calcofluor (28 µg/ml)**	**C1 (4 µg/ml)**	**C1 (4 µg/ml) +calcofluor (28 µg/ml)**	**C1 (16 µg/ml)**	**C1 (16 µg/ml) +calcofluor (28 µg/ml)**
Optical density (OD_{600})	1.210	1.123	0.539	0.252	0.232	0.072
	1.042	1.045	0.528	0.242	0.235	0.031
	1.042	1.042	0.525	0.342	0.283	0.028
	1.091	1.127	0.489	0.253	0.242	0.028
	1.251	1.201	0.523	0.342	0.287	0.021
	1.248	1.12	0.531	0.202	0.182	0.038
	1.250	1.034	0.426	0.321	0.301	0.082
	1.243	1.128	0.528	0.142	0.223	0.039
	1.212	1.213	0.423	0.193	0.232	0.181
	1.142	1.163	0.521	0.125	0.198	0.082
	1.080	1.043	0.511	0.192	0.243	0.072
	1.124	1.172	0.446	0.125	0.332	0.082
mean	1.161	1.118	0.499	0.228	0.249	0.063
standard deviation	0.084	0.064	0.043	0.079	0.044	0.044

Table N. The rate of *C. albicans* NCPF 3153 spheroplast lysis under enzymatic digestion of the cell wall in untreated (control) and C1-treated cells. Results from this table were used for preparing Fig 7 in the main body of the paper.

		Time [min]						
		**0**	**10**	**20**	**30**	**40**	**50**	**60**
OD 600	control	0,684	0,621	0,582	0,522	0,482	0,401	0,291
		0,668	0,631	0,621	0,534	0,492	0,451	0,312
		0,659	0,612	0,605	0,511	0,432	0,398	0,341
		0,662	0,632	0,573	0,581	0,401	0,392	0,372
		0,669	0,641	0,578	0,498	0,502	0,321	0,294
		0,681	0,611	0,571	0,521	0,452	0,382	0,316
	average	0,6705	0,624667	0,588333	0,527833	0,460167	0,390833	0,321
	SD	0,009179	0,010965	0,018382	0,026201	0,035592	0,038146	0,028095
	C1 32 µg/ml	0,693	0,601	0,432	0,215	0,123	0,172	0,012
		0,673	0,562	0,482	0,272	0,183	0,173	0,083
		0,662	0,611	0,491	0,301	0,216	0,162	0,082
		0,638	0,572	0,46	0,287	0,198	0,183	0,052
		0,664	0,591	0,412	0,312	0,271	0,162	0,082
		0,672	0,585	0,482	0,263	0,182	0,142	0,083
	average	0,667	0,587	0,459833	0,275	0,1955	0,165667	0,065667
	SD	0,016391	0,016543	0,028916	0,03147	0,044199	0,012789	0,026462

Table O. Ergosterol level in *C. albicans* NCPF 3153 cells treated with FLC, AmB, and the C1 compound. Results from this table were used for preparing Fig 8 in the main body of the paper.

Control	Fluconazole 4 µg/ml		AmB 0.25 µg/ml		C1 16 µg/ml		C1 32 µg/ml	
% ergosterol /wet weight	% ergosterol /wet weight	% of control	% ergosterol /wet weight	% of control	% ergosterol /wet weight	% of control	% ergosterol /wet weight	% of control
0.0112	0.0057	33.46	0.0178	104.50	0.0171	100.39	0.0182	106.85
0.0215	0.0063	36.99	0.0195	114.48	0.0165	96.87	0.0174	102.15
0.0184	0.0048	28.18	0.0189	110.96	0.0187	109.78	0.0192	112.72

Table P. **Viability of normal human dermal fibroblasts (NHDF) after 96-h *in vitro* culture in the presence of the C1 compound at the concentrations of 4-512 µg/ml measured spectrophotometrically at 590m nm using the MTT assay**. Results from this table were transformed into control percentage, presented in the table R.

		**Concentration of C1 [µg/ml]**								
		**Control**	**4**	**8**	**16**	**32**	**64**	**128**	**256**	**512**
Absorbance at 590 nm	1 repetition	0.794	0.679	0.805	0.798	0.643	0.629	0.585	0.638	0.419
		0.712	0.622	0.724	0.684	0.691	0.716	0.760	0.667	0.498
		0.685	0.628	0.593	0.718	0.649	0.747	0.671	0.680	0.348
		0.736	0.707	0.557	0.605	0.810	0.858	0.737	0.615	0.470
		0.656	0.880	0.746	0.719	0.793	0.724	0.822	0.752	0.421
		0.629	0.680	0.824	0.739	0.809	0.740	0.590	0.617	0.480
		0.728	0.682	0.872	0.732	0.783	0.883	0.868	0.534	0.366
		0.532	0.759	0.879	0.796	0.880	0.823	0.830	0.684	0.301
	2 repetition	0.879	0.873	0.985	0.783	0.918	0.753	0.567	0.645	0.543
		0.822	0.673	0.691	0.783	0.842	0.732	0.774	0.754	0.459
		0.628	0.852	0.661	0.678	0.790	0.743	0.643	0.745	0.654
		0.707	0.741	0.732	0.690	0.683	0.678	0.634	0.654	0.444
		0.880	0.687	0.725	0.736	0.782	0.745	0.608	0.513	0.473
		0.880	0.687	0.701	0.743	0.845	0.803	0.623	0.643	0.535
		0.682	0.651	0.693	0.672	0.875	0.668	0.595	0.412	0.394
		0.759	0.726	0.980	0.513	0.739	0.662	0.754	0.743	0.678
	3 repetition	0.654	0.691	0.758	0.745	0.573	0.654	0.734	0.754	0.498
		0.872	0.634	0.745	0.603	0.589	0.678	0.745	0.643	0.486
		0.643	0.727	0.643	0.645	0.503	0.754	0.654	0.407	0.356
		0.761	0.683	0.992	0.702	0.663	0.822	0.765	0.743	0.387
		0.726	0.662	0.637	0.673	0.873	0.645	0.755	0.783	0.374
		0.546	0.798	0.983	0.674	0.783	0.752	0.745	0.765	0.489
		0.786	0.793	0.683	0.932	0.932	0.745	0.593	0.473	0.593
		0.648	0.534	0.997	0.623	0.843	0.643	0.595	0.783	0.488

Table R. **Percentage viability of normal human dermal fibroblasts (NHDF) in relation to the control cells after 96-h *in vitro* culture in the presence of the C1 compound at the concentrations of 4-512 µg/ml measured spectrophotometrically at 590m nm using the MTT assay**. Results from this table were used for preparing Fig 10 in the main body of the paper.

		**Concentration of C1 [µg/ml]**							
		**4**	**8**	**16**	**32**	**64**	**128**	**256**	**512**
Percentage viability of treated cells in relation to the control cells	1 repetition	93.90	111.31	110.32	89.17	86.94	80.91	88.22	58.02
		86.09	100.11	94.56	95.53	99.00	105.10	92.21	68.92
		86.90	82.05	99.36	89.70	103.33	92.79	94.02	48.08
		97.77	77.03	83.65	112.03	118.66	101.92	85.03	64.98
		121.77	103.24	99.45	109.71	100.11	113.75	104.00	58.26
		94.11	113.93	102.23	111.95	102.31	81.67	85.38	66.33
		94.32	120.57	101.26	108.27	122.19	120.07	73.80	50.68
		104.98	121.56	110.08	121.77	113.78	114.81	94.59	41.66
	2 repetition	120.75	136.24	108.30	126.97	104.15	78.42	89.21	75.10
		93.08	95.57	108.30	116.46	101.24	107.05	104.33	63.49
		117.84	91.42	93.78	109.27	102.77	88.93	103.04	90.51
		102.49	101.24	95.44	94.47	93.78	87.73	90.46	61.41
		95.02	100.28	101.80	108.16	103.04	84.09	70.95	65.42
		95.02	96.96	102.77	116.87	111.07	86.17	88.93	74.00
		90.04	95.85	92.95	121.02	92.39	82.30	56.98	54.50
		100.41	135.55	70.95	102.21	91.56	104.29	102.77	93.78
	3 repetition	95.57	104.84	103.04	79.25	90.46	101.52	104.29	68.88
		87.69	103.04	83.40	81.47	93.78	103.04	88.93	67.22
		100.55	88.93	89.21	69.57	104.29	90.46	56.29	49.24
		94.47	137.21	97.10	91.70	113.69	105.81	102.77	53.53
		91.56	88.11	93.08	120.75	89.21	104.43	108.30	51.73
		110.37	135.96	93.22	108.30	104.01	103.04	105.81	67.63
		109.68	94.47	128.91	128.91	103.04	82.02	65.42	82.02
		73.86	137.90	86.17	116.60	88.93	82.30	108.30	67,50
Average		98.26	107.22	97.89	105.42	101.41	95.94	90.17	64.29
Standard deviation		11,20	18.21	11.25	15.48	9.35	12.19	15.23	12.59
